# FtsZ-Ring Regulation and Cell Division Are Mediated by Essential EzrA and Accessory Proteins ZapA and ZapJ in *Streptococcus pneumoniae*

**DOI:** 10.3389/fmicb.2021.780864

**Published:** 2021-12-02

**Authors:** Amilcar J. Perez, Jesus Bazan Villicana, Ho-Ching T. Tsui, Madeline L. Danforth, Mattia Benedet, Orietta Massidda, Malcolm E. Winkler

**Affiliations:** ^1^Department of Biology, Indiana University Bloomington, Bloomington, IN, United States; ^2^Department of Cellular, Computational and Integrative Biology (CIBIO), University of Trento, Trento, Italy

**Keywords:** EzrA function, FtsZ-ring formation, MapZ(LocZ), SepF, ZapA, ZapJ (Spd_1350), localization of peptidoglycan synthesis

## Abstract

The bacterial FtsZ-ring initiates division by recruiting a large repertoire of proteins (the divisome; Z-ring) needed for septation and separation of cells. Although FtsZ is essential and its role as the main orchestrator of cell division is conserved in most eubacteria, the regulators of Z-ring presence and positioning are not universal. This study characterizes factors that regulate divisome presence and placement in the ovoid-shaped pathogen, *Streptococcus pneumoniae* (*Spn*), focusing on FtsZ, EzrA, SepF, ZapA, and ZapJ, which is reported here as a partner of ZapA. Epi-fluorescence microscopy (EFm) and high-resolution microscopy experiments showed that FtsZ and EzrA co-localize during the entire *Spn* cell cycle, whereas ZapA and ZapJ are late-arriving divisome proteins. Depletion and conditional mutants demonstrate that EzrA is essential in *Spn* and required for normal cell growth, size, shape homeostasis, and chromosome segregation. Moreover, EzrA(*Spn*) is required for midcell placement of FtsZ-rings and PG synthesis. Notably, overexpression of EzrA leads to the appearance of extra Z-rings in *Spn*. Together, these observations support a role for EzrA as a positive regulator of FtsZ-ring formation in *Spn.* Conversely, FtsZ is required for EzrA recruitment to equatorial rings and for the organization of PG synthesis. In contrast to EzrA depletion, which causes a bacteriostatic phenotype in *Spn*, depletion of FtsZ results in enlarged spherical cells that are subject to LytA-dependent autolysis. Co-immunoprecipitation and bacterial two-hybrid assays show that EzrA(*Spn*) is in complexes with FtsZ, Z-ring regulators (FtsA, SepF, ZapA, MapZ), division proteins (FtsK, StkP), and proteins that mediate peptidoglycan synthesis (GpsB, aPBP1a), consistent with a role for EzrA at the interface of cell division and PG synthesis. In contrast to the essentiality of FtsZ and EzrA, ZapA and SepF have accessory roles in regulating pneumococcal physiology. We further show that ZapA interacts with a non-ZapB homolog, named here as ZapJ, which is conserved in *Streptococcus* species. The absence of the accessory proteins, ZapA, ZapJ, and SepF, exacerbates growth defects when EzrA is depleted or MapZ is deleted. Taken together, these results provide new information about the spatially and temporally distinct proteins that regulate FtsZ-ring organization and cell division in *Spn*.

## Introduction

Bacterial cell division initiates by polymerization of the highly conserved and essential tubulin-like protein FtsZ into a dynamic Z-ring composed of FtsZ treadmilling filaments (Bi and Lutkenhaus, 1991; Lutkenhaus et al., 2012; Haeusser and Margolin, 2016; Bisson-Filho et al., 2017; Yang et al., 2017; Perez et al., 2019). The dynamic Z-ring recruits a large number of proteins into the divisome needed for septal peptidoglycan (PG) synthesis, septation, and separation of cells (Den Blaauwen et al., 2003; Aarsman et al., 2005; Gamba et al., 2009; Trip and Scheffers, 2015). Many studies of FtsZ-ring regulation and cell division have been performed on model rod-shaped bacteria, including *Escherichia coli* (*Eco*; Gram-negative), *Bacillus subtilis* (*Bsu*; Gram-positive), and *Caulobacter crescentus* (*Ccr*; Gram-negative) (Thanbichler and Shapiro, 2006; Coltharp et al., 2016, Yu et al., 2021). In these bacteria, the position of the Z-ring is dictated largely by negative regulatory systems (Min and nucleoid occlusion in *Bsu* and *Eco* and MipZ in *Ccr*); yet, a many other bacteria with different cell shapes do not follow the paradigms from these model rod-shaped bacteria (reviewed by Monahan et al., 2014). *Streptococcus pneumoniae* (*Spn*; pneumococcus) is a low-GC Gram-positive, ovoid-shaped commensal bacterium that can act as a drug-resistant, opportunistic pathogen and serious threat to human health (Weiser et al., 2018). *Spn* and other species of *Streptococci* lack Min and nucleoid occlusion systems and form Z-rings over the nucleoid at early stages of division (Land et al., 2013). The mechanisms leading to midcell Z-ring placement and regulation in cell division and septal PG synthesis have only recently begun to be understood in ovoid-shaped bacteria like *Spn* (Briggs et al., 2021), despite their potential to reveal vulnerabilities for the discovery of new antibiotics and vaccines.

The ovoid shape of *Spn* is maintained by a thick PG layer that provides protection against osmotic stress-induced cell lysis (Vollmer et al., 2019). Morphogenesis of the cell wall into an ovoid shape is achieved through the activities and coordination of two PG synthesis systems that occur at the midcell plane, where the FtsZ-ring assembles (Supplementary Figure 1A). Septal peptidoglycan synthesis (sPG) is carried out in part by the bPBP2x:FtsW PG synthase that inserts PG at the leading edge of the closing septal annulus (Perez et al., 2021). Peripheral PG (pPG) synthesis is mediated by the bPBP2b:RodA synthase pair that catalyzes cellular elongation pushing outward from the midcell ring (Supplementary Figure 1A, middle). In pneumococcus, the sPG and pPG syntheses occur nearly simultaneously throughout the cell cycle (Wheeler et al., 2011; Perez et al., 2021; Trouve et al., 2021). Unlike rod-shaped *Bsu* and *Eco*, *Spn* lacks MreB actin-like proteins to organize lateral cell-wall synthesis (Vollmer et al., 2019). In *Spn*, FtsZ and its closely associated proteins initially organize the sPG and pPG synthesis machines in a single ring at the equators of newly divided cells (Supplementary Figure 1A, top) (Massidda et al., 2013; Briggs et al., 2021; Perez et al., 2021). As septation and elongation begin, the Z-ring moves as an inner ring at the leading edge of the closing septal annulus, while pPG synthesis occurs in a separate outer ring lacking FtsZ at the base of the septal annulus (Supplementary Figure 1A, middle) (Perez et al., 2021). Thus, FtsZ-ring regulation initially organizes both cell division and elongation in *Spn*.

In ovoid-shaped bacteria, such as *Spn*, MapZ (LocZ) positively regulates FtsZ-ring placement at the equators of daughter cells (Fleurie et al., 2014a; Holeckova et al., 2015). MapZ-rings bound to PG split from the initial midcell Z-ring of newly divided cells and are moved, presumably by pPG synthesis, toward the future equators of daughter cells (Supplementary Figure 1A, middle (Fleurie et al., 2014a; Holeckova et al., 2015). Recent dynamic studies demonstrate that the MapZ-ring forms a stable structure in *Streptococcus* species (Li et al., 2018; Perez et al., 2019). In *Spn*, the MapZ-ring guides nascent treadmilling FtsZ filaments and bundles continuously from the earliest stage of division over an approximate ≈9-min period out to the future equatorial rings (Perez et al., 2019). With time, the amount of FtsZ associated with MapZ-rings increases, while the amount of septal FtsZ decreases and disappears before septation is complete (Tsui et al., 2014; Perez et al., 2019). In contrast, *Streptococcus mutans* (*Smu*) MapZ seems to move toward future equators in the absence of nascent FtsZ filaments and bundles (Li et al., 2018). Once MapZ has reached the new equators, FtsZ filaments/bundles rapidly stream from the septum to the equatorial MapZ-ring (Li et al., 2018).

Along with MapZ, chromosome segregation positively regulates Z-ring placement in *Spn*, as indicated by misplaced Z-rings in Δ*mapZ* or Δ*smc* mutants (van Raaphorst et al., 2017). However, Δ*mapZ* and Δ*smc* are not synthetically lethal (van Raaphorst et al., 2017). Other Z-ring-associated proteins or regulators in *Spn* have recently been studied, including FtsA, SepF, RocS, and CcrZ (Mura et al., 2016; Zheng et al., 2017; Mercy et al., 2019; Gallay et al., 2021), but besides FtsA, none has been shown to be essential. Therefore, it has remained an unanswered question whether other essential components are involved in Z-ring assembly and PG synthesis in *Spn*. In this work, we elucidate the relationship of the key Z-ring regulators EzrA and ZapA that were first identified in *Bsu*.

EzrA is conserved in low-GC, Gram-positive bacteria (Levin et al., 1999). The 2D-protein topology of EzrA consists of an N-terminus transmembrane domain followed by cytoplasmic C-terminus, whose crystal structure consists of spectrin-like repeats (Haeusser et al., 2007; Cleverley et al., 2014; Land et al., 2014). Cellular and *in vitro* studies suggested that a primary function of EzrA(*Bsu*) is to inhibit aberrant FtsZ assembly and division at cell poles, thereby acting as a negative Z-ring regulator (Chung et al., 2007; Land et al., 2014). Yet, contrary to acting as a negative regulator, EzrA(*Bsu*) is among the first group of proteins to localize at the midcell, simultaneously with FtsZ, FtsA, and ZapA, but prior to GpsB, Pbp2b, FtsL, DivIVB, and DivIVA (Gamba et al., 2009). EzrA(*Bsu*) midcell localization is dependent on FtsZ as well as the EzrA(*Bsu)*-QNR motif that is conserved in all EzrA homolog proteins (Haeusser et al., 2007). At the midcell, EzrA(*Bsu)*, ZapA, and SepF function to condense treadmilling FtsZ-filaments into an FtsZ-ring that can then act to promote cell division and sPG synthesis in *Bsu* (Squyres et al., 2021). The presence of EzrA(*Bsu*) decreases FtsZ subunit lifetimes and FtsZ filament lengths without affecting FtsZ-treadmilling speeds (Squyres et al., 2021). In addition to regulating Z-ring dynamics, fluorescent microscopy and bacterial two-hybrid (B2H) assays suggest that EzrA(*Bsu*) acts in concert with other cell division proteins, including GpsB(*Bsu*), to shuttle the major class A penicillin-binding protein (aPBP1) from the elongation to the division machinery in *Bsu* (Claessen et al., 2008). Interactions between GpsB(*Bsu*) and EzrA(*Bsu*) have been shown by B2H assays (Claessen et al., 2008; Pompeo et al., 2015).

Additional studies of EzrA functions have been performed in coccus-shaped *Staphylococcus aureus* (*Sau*) and ovococcus-shaped *Streptococcus mutans* (*Smu*). Biochemical studies showed that the N-terminal domain of EzrA(*Sau*) interacts with the C-terminal tail of FtsZ(*Sau*) (Son and Lee, 2013). Physiological studies by two different groups contest the essentiality of EzrA*(Sau)* (Jorge et al., 2011; Steele et al., 2011). One study concluded from depletion experiments that EzrA*(Sau)* is required for *Sau* cell growth (Steele et al., 2011). B2H assays in this study further indicated that EzrA(*Sau*) interacts with itself and potentially interacts directly with numerous division and PG synthesis proteins, including FtsZ, FtsA, FtsL, FtsW, DivIB, DivIC, PBP1, PBP2, PBP3, SepF, GpsB, and RodA (Steele et al., 2011). By contrast, a second study concluded that EzrA(*Sau*) is not essential, based on multiple depletion approaches of EzrA(*Sau*) in different *Sau* genetic backgrounds; rather, EzrA(*Sau*) is important for cell size homeostasis (Jorge et al., 2011). The second study also showed by B2H assays that EzrA(*Sau*) interacts with itself, PBP1, and PBP2. In *Smu*, a species distantly related to *Spn* (Richards et al., 2014), EzrA(*Smu*) is not essential, and deletion of EzrA(*Smu*) results in an increased doubling time (≈1.7-fold), shorter and wider cells, and irregular localization of FtsZ compared to wild-type (WT) cells (Xiang et al., 2019). However, a comprehensive study of EzrA(*Smu*) in cell division and PG synthesis has not been reported.

EzrA(*Spn*) was classified as essential by transposon-sequencing (Tn-Seq) screens (van Opijnen et al., 2009), high-throughput gene disruption assays (Thanassi et al., 2002), and CRISPRi experiments (Liu et al., 2017). The spectrin-repeat structure *of* EzrA(*Bsu*) (Cleverley et al., 2014) is conserved in EzrA(*Spn*) based on modeling with the Phyre2 program (Supplementary Figure 1D) (Kelley et al., 2015). Coiled-coil analysis demonstrates that EzrA(*Spn*) has four coiled-coil repeats in addition to the transmembrane domain (Supplementary Figure 1C), similar to EzrA(*Bsu*) (Land et al., 2014). The topology of EzrA(*Spn*) has an extracellular N-terminus attached to a transmembrane domain (amino acids 5-27) followed by the large intracellular spectrin-like domain in the C-terminus (amino acids 28-575; Supplementary Figure 1E).

Bacterial two-hybrid and surface plasmon resonance experiments showed that EzrA(*Spn*) interacts with GpsB, DivIVA, and FtsZ (Fleurie et al., 2014b; Rued et al., 2017). Co-immunoprecipitation (Co-IP) experiments further demonstrated that EzrA(*Spn*) is in a complex with FtsZ, GpsB, and StkP in *Spn* cells, and it was postulated that EzrA(*Spn*) acts as a bridge connecting FtsZ to other cell division proteins (Rued et al., 2017). TIRF microscopy (TIRFm) of EzrA(*Spn*)-GFP expressed from its native chromosomal locus showed that EzrA(*Spn*) and FtsA move circumferentially at ≈30 nm/s with treadmilling FtsZ filaments/bundles in nascent rings containing MapZ that move toward the future equators of dividing daughter cells (Perez et al., 2019). Strikingly, in Δ*mapZ* mutants, EzrA(*Spn*), presumably associated with FtsZ filaments/bundles, streams from division septa to equators and other positions in daughter cells, indicating a failsafe mechanism for Z-ring placement when MapZ is absent (Perez et al., 2019). In addition, at a semi-permissive temperature (37°C), the temperature-sensitive *ezrA*(T506I) mutant forms cells with larger diameters in which the regular spacing of nodes of PG synthesis are disrupted (Perez et al., 2021). Other roles of EzrA(*Spn*) in Z-ring placement, growth, morphology, and viability have not yet been described.

Unlike Gram-positive EzrA, ZapA is well conserved in both Gram-positive and Gram-negative bacteria, where its role in Z-ring bundling and ordering has been established (Caldas et al., 2019). While ZapA, SepF, and EzrA are not individually essential in *Bsu*, combined mutations result in synthetic-lethal phenotypes, including *ezrA* and *sepF* (Duman et al., 2013), *ftsA* and *sepF* (Ishikawa et al., 2006), and *zapA* and *ezrA* (Gueiros-Filho and Losick, 2002). Besides regulating Z-ring dynamics in *E. coli*, ZapA is part of a multilayered network of proteins that connect and coordinate the Z-ring to the chromosome *via* ZapB and MatP (Buss et al., 2015). In contrast, nothing has been reported about ZapA(*Spn*) function or whether ZapA(*Spn*) has a partner subunit, since ZapB homologs are absent in *Spn.*

In this paper, we fill in some of these gaps about EzrA(*Spn*) and ZapA(*Spn*) functions and interactions. We report the association of FtsZ and EzrA during the entire *Spn* cell cycle. We also characterize the essential intertwined roles of FtsZ and EzrA in *Spn* cell division and organizing PG synthesis. We show that depletion of EzrA(*Spn*) is required for midcell Z-ring divisome assembly, whereas overexpression of EzrA(*Spn*) leads to the appearance of extra Z-rings in cells, opposite to the phenotypes observed in *Bsu* (Haeusser et al., 2004). Our combined results are consistent with a role for EzrA(*Spn*) as a positive, rather than a negative, regulator of FtsZ-ring formation in *Spn.* In addition, Co-IP and B2H experiments show that EzrA(*Spn*) is found in complexes with numerous proteins, including FtsZ, Z-ring regulators, division proteins, and PG synthesis proteins. This versatility of EzrA(*Spn*) in forming multicomponent complexes, possibly through direct interactions, is consistent with EzrA(*Spn*) acting to link and modulate cell division and PG synthesis. Finally, we discovered the interaction partner (ZapJ) of ZapA(*Spn*) and show that non-essential ZapA, ZapJ, and SepF act as accessory proteins to essential EzrA, possibly by forming a spatially separated network of positive Z-ring regulators.

## Materials and Methods

Complete descriptions of the following materials and methods used in this study are contained in *Supplementary Material* based on the references indicated: EzrA(*Spn*) structure modeling (Kelley et al., 2015; PyMol); construction of bacterial strains and growth conditions (Supplementary Tables 1, 2; Lanie et al., 2007; Tsui et al., 2010, 2014; Land et al., 2013); growth of merodiploid strains and Zn-dependent depletion (Jacobsen et al., 2011; Tsui et al., 2014, 2016); cell fixation and adherence to coverslips for fluorescence microscopy (Fm) (Tsui et al., 2016); characterization of antibodies for immunofluorescence microscopy (IFM) (Supplementary Table 5; Land et al., 2013; Tsui et al., 2014, 2016); analysis of 2D-epifluorescence microscopy (EFm) images (Land et al., 2013; Tsui et al., 2014; Perez et al., 2019); 3D-SIM IFM (Land et al., 2013; Tsui et al., 2014); TIRF microscopy (TIRFm) (Grimm et al., 2015; Perez et al., 2019); 3D-SIM of FDAA-labeled cells expressing EzrA(*Spn*)-sfGFP (Perez et al., 2021); measurements of cell dimensions by phase-contrast microscopy (PCm); quantitative Western blotting (Lara et al., 2005; Wayne et al., 2010; Land and Winkler, 2011; Beilharz et al., 2012; Cleverley et al., 2019); Live/Dead staining of *ezrA* and other mutants (Wayne et al., 2012; Sham et al., 2013); DAPI staining for nucleiod content (Perez et al., 2019); FDAA pulse-chase labeling in depletion experiments (Boersma et al., 2015; Perez et al., 2021); co-immunoprecipitation (Co-IP) assays (Rued et al., 2017); bacterial two-hybrid (B2H) assays (Supplementary Tables 6, 7); Karimova et al., 2005; Rued et al., 2017; Cleverley et al., 2019); and mass spectrometry (MS) to identify ZapJ (Spd_1350) (Sham et al., 2011; Zheng et al., 2017).

## Results

### Localization of EzrA and FtsZ Is Highly Correlated During the Entire *Spn* Cell Cycle

**FIGURE 1:**
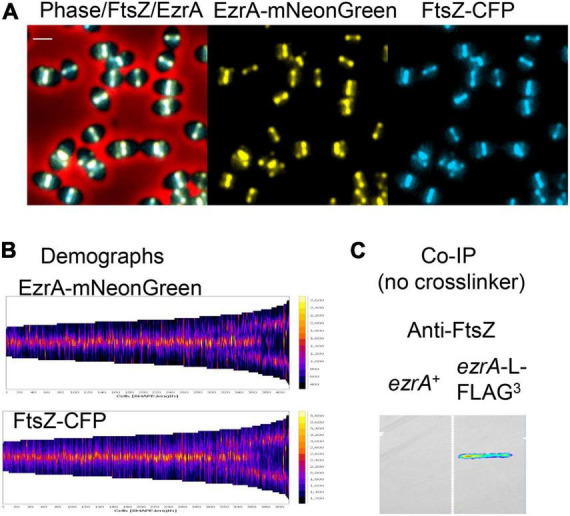
Highly correlated localization patterns of FtsZ and EzrA during the *Spn* cell cycle. Cells from at least two independent biological replicates were collected at OD_620_ ≈ 0.1–0.2 and prepared for microscopy or co-IP as described in section “Materials and Methods.” **(A)** Representative field of live cells expressing EzrA-mNeonGreen and FtsZ-CFP (IU14153). Scale bar is 1.0 μm. **(B)** Demographs showing FtsZ and EzrA localization from shorter pre-divisional cells (left side) to longer post-divisional cells (right side). **(C)** Membrane probed with anti-FtsZ showing EzrA-FLAG^3^ (IU5456) can co-IP FtsZ from non-crosslinked *Spn* cells relative to untagged EzrA^+^ (IU10447).

The strong association of EzrA(*Spn*) and FtsZ(*Spn*) was demonstrated from co-IP experiments showing that EzrA as bait can pull down FtsZ in *Spn* extracts without the cross-linking procedure (Figure 1C). To determine the spatiotemporal relationship of EzrA relative to FtsZ during the *Spn* cell cycle, we performed EFm on live cells expressing fluorescent-protein fusions of FtsZ and EzrA and IFM on fixed cells expressing epitope-tagged FtsZ and EzrA, as described in section “Materials and Methods.” Unless otherwise noted, all strains in this study did not show detectable growth or cell shape defects when fusion proteins were expressed as the only copy in the cell from native promoters (Supplementary Table 1 and data not shown). Linkers in fusion constructs are omitted in the text for simplicity, but are listed in Supplementary Tables 1, 2.

In live cells of strain IU14153, C-terminal fusion proteins EzrA-mNeonGreen and FtsZ-CFP showed complete overlap by EFm (Figure 1A). Demographs generated with MicrobeJ (Ducret et al., 2016) of fields of these unsynchronized cells at different stages of division confirmed overlap of EzrA and FtsZ and indicated simultaneous movement of the proteins from septa to developing equators of daughter cells, including the stage at which three bands are present (Figure 1B). Likewise, 2D-IFM images of fixed cells expressing epitope-tagged EzrA-HA and FtsZ-Myc (strains IU7223 or IU9713) indicated overlap at all stages of cell division (Supplementary Figure 2A, left). Cells were retrospectively binned into four stages, and fluorescence signals of EzrA-HA, FtsZ-Myc, and DNA nucleoids (DAPI) were averaged as described previously (Land et al., 2013; Supplementary Figure 2B). This quantitative analysis confirmed overlap of EzrA and FtsZ throughout the cell cycle (Supplementary Figures 2B,C). Averaged images at each division stage indicated that the EzrA-ring is slightly larger than the FtsZ-ring, similar to the slightly larger FtsA-ring in relation to the FtsZ-ring (Mura et al., 2016). This conclusion was confirmed by Student t-test analysis of the distributions of band diameters in each cell (Supplementary Figure 2C) (see Tsui et al., 2014). This slight difference in diameters coincides with location of the C-terminus of EzrA closer to the membrane than FtsZ (Supplementary Figures 1C,D), whose apparent distance is likely enhanced by the length of the fluorescently labeled antibodies used in IFM.

High-resolution 3D-SIM IFM images (Supplementary Figure 3) confirmed and extended results from 2D-EFm. For each stage of division, > 20 cells were examined by 3D-SIM. Stage 1 cells show highly correlated spatial organization of EzrA and FtsZ, with the diameter of EzrA slightly larger than that of FtsZ in rotated images (Supplementary Figure 3). Most EzrA and FtsZ are visible at the septa of all Stage 2 cells examined, and in ≈35% of cells, faint rings containing EzrA and FtsZ are detected in nascent rings moving toward the equatorial regions of daughter cells (arrow, Supplementary Figure 3, Stage 2) (Perez et al., 2019). Later in division, EzrA and FtsZ locate in a characteristic three-ring pattern at the septum and the equators of daughter cells over the separating nucleoid (Supplementary Figure 3, Stages 2.5 and 3). In Stage 4 cells, most EzrA and FtsZ locate to the new equatorial rings of the daughter cells, sometimes leaving a small dot of each protein at the closing septum (Supplementary Figures 2B, 3).

We extended these analyses to further characterize FtsZ and EzrA localization in a Δ*mapZ* mutant. Certain FtsZ-GFP fusions are partially functional in Δ*mapZ* mutants, resulting in more severe morphological defects than in strains containing Δ*mapZ* alone (Perez et al., 2019). We did not observe these compounded defects in a Δ*mapZ* mutant expressing epitope-tagged EzrA-HA and FtsZ-Myc by PCm or growth curve analysis (Supplementary Figure 2A, right and data not shown). Previously, we showed that in the absence of MapZ, EzrA moves to daughter cells by a delayed and aberrant streaming mechanism that was rarely (1%) seen in WT cells (Perez et al., 2019). This secondary, failsafe mechanism is responsible for placement of Z-rings near the equators of most, but not all, Δ*mapZ* daughter cells (Supplementary Figure 2A, right). EzrA and FtsZ co-localized during delayed streaming in late-divisional Δ*mapZ* cells, as observed by 2D-EFm (Supplementary Figures 2A,B, stage 4) and at high resolution by 3D-SIM (Supplementary Figure 4). Together, these results confirm that a tight association between EzrA and FtsZ filaments/bundles is maintained during this alternate failsafe streaming mechanism.

Previously, we reported that EzrA or FtsA moves circumferentially at the same velocity as treadmilling FtsZ filaments/bundles in nascent rings or early equatorial rings during the *Spn* cell division (Perez et al., 2019). By contrast, the density of molecules was too great in septal rings or fully developed equatorial rings to determine dynamic patterns by TIRFm. We further evaluated how tightly associated dynamic patterns of EzrA are with FtsZ or FtsA in developing equatorial or nascent rings. In these experiments, we performed dual-color TIRFm where we tracked native locus-expressed FtsZ-sfGFP or GFP-FtsA alongside EzrA-HaloTag (EzrA-HT) labeled to saturation with the HaloTag JF549 ligand (Grimm et al., 2015; Perez et al., 2019). Kymographs along equatorial and nascent-ring planes indicate that for the most part, the movement of FtsZ filaments/bundles or FtsA in FtsZ filaments/bundles mirrored that of EzrA in time and space, indicating a strong dynamic association (Supplementary Figure 5). In addition, we observed some events consistent with unassociated EzrA, as indicated by lack of FtsZ or FtsA and vertical EzrA traces (magenta lines, Supplementary Figure 5). Together, we conclude that EzrA is tightly associated with circumferentially moving FtsZ filaments/bundles that also contain FtsA, although some EzrA is not associated with moving FtsZ filaments/bundles.

### EzrA and PG Synthesis Show Different Localization Patterns in *Spn*

Several reports suggest that EzrA is associated with PBPs and PG synthesis in *Bsu* and *Sau* (Claessen et al., 2008; Jorge et al., 2011; Steele et al., 2011). In *Spn*, PG synthesis enzymes and activity localize late in division from septa to new equatorial FtsZ-rings in daughter cells (Tsui et al., 2010; Land et al., 2013; Perez et al., 2019). Given the strong association between FtsZ and EzrA described above, we postulated that EzrA would precede PG synthesis to the new equatorial rings of daughter cells. To localize EzrA relative to PG synthesis, we carried out short pulse (2.5 min) labeling with a fluorescent D-amino acid (FDAA) in a strain expressing EzrA-sfGFP from the *ezrA* native locus (strain IU10254) as described in *Materials and Methods* (Supplementary Figure 6). FDAAs are incorporated into the PG in regions of active PBP transpeptidase activity (Kuru et al., 2012; Tsui et al., 2014; Boersma et al., 2015). Cells were binned by division stage, and average fluorescence intensities were quantitated as described above for EFm images (Supplementary Figure 2). Statistical analysis of labeling widths shows that the FDAA labeling was greater than the EzrA width at all division stages (Supplementary Figure 6B). In early stage-1 and -2 cells, PG synthesis and EzrA are confined to division septa. In later stage-3 and -4 cells, EzrA shows the same pattern reported previously for FtsZ (Land et al., 2013). PG synthesis remains confined to the septum, while a substantial amount of EzrA has moved to the equators of daughter cells (Supplementary Figure 6A). By stage 4, little EzrA remains at the septum compared to small region of PG synthesis.

3D-SIM images of vertically oriented cells confirm the separation of the FDAA labeling and EzrA in early-stage 1 and 2 cells (Supplementary Figure 6C). In stage 1 cells, the separation is small and consistent with the fact that PG synthesis occurs outside of cells, whereas the sfGFP-fused C-terminus of EzrA is inside the cell near the membrane (Supplementary Figures 1D, 6C). The increased separation of labeling in stage 2 cells is consistent with the recent report that the contracting FtsZ-ring tracks with an inner ring of sPG synthesis at the leading edge of the septal annulus, while an outer concentric ring of pPG elongation synthesis remains at the outer edge of the septal disk (Perez et al., 2021). We conclude that EzrA is tracking in a pattern reported before for FtsZ and FtsA that is different from that of PG synthesis.

### EzrA Is Essential in *Spn*

To directly test the essentiality of EzrA(*Spn*), we performed assays for recovery of colonies following transformation with amplicons containing null mutations, as described in section “Materials and Methods.” We transformed an amplicon containing a null Δ*ezrA*<>*aad9* allele, where the *ezrA* ORF is replaced by an ORF imparting spectinomycin resistance, into an unencapsulated derivative of strain D39W (IU1945; D39 Δ*cps*) and compared the number of transformants with a positive-control amplicon (Δ*purR*<>*aad9*) and a negative-control amplicon (Δ*ftsZ*::*aad9*) (Table 1). Following 24 h of incubation, the Δ*purR* control showed many transformants, while Δ*ezrA* or Δ*ftsZ* showed none (Table 1, column 1). After 48 h of incubation, a limited number of small, variable-sized transformant colonies, indicative of potential suppressor mutations, were observed for Δ*ezrA*, but not Δ*ftsZ* (Table 1). These potential Δ*ezrA* transformants showed similar cell growth as the EzrA depletion strains described next; however, they were unstable and could not be recovered after storage and were not further characterized here.

**TABLE 1:** Relative transformation of Δ*ezrA* or Δ*ftsZ* amplicon into *Spn* D39 Δ*cps*^a,b^.

	Number of colonies in recipient strain after 16-20 h incubation				
Amplicon	D39 Δ*cps*	D39 Δ*cps bgaA*’::P_Zn_-*ezrA*^+^		D39 Δ*cps bgaA*’::P_Zn_-*ftsZ*^+^	
		+Zn	-Zn	+Zn	-Zn
1. Δ*ezrA*<>*aad9*	0^d^	> 300	< 15^c, d^	0	0
2. Δ*ftsZ*::*aad*9	0	0	0	> 300	0
3. Δ*purR*::*aad*9	> 300	>300	> 300	NT	NT

*^a^D39 Δcps (IU1945), D39 Δcps bgaA’::P_Zn_-ezrA^+^ (IU8795), D39 Δcps bgaA’::P_Zn_-ftsZ^+^ (IU8122) were grown and transformed as described in section “Materials and Methods.” Transformation reactions requiring ZnCl_2_ were grown (for 1 h before addition of ΔezrA or ΔftsZ amplicon), transformed, and plated in the presence of 0.5 mM ZnCl_2_ and 0.05 mM MnSO_4_ for transformation with ΔezrA<>aad9 amplicons or 0.3 mM ZnCl_2_ and 0.03 mM MnSO_4_ for transformation with ΔftsZ::aad9 amplicon. Numbers of colonies indicated were obtained from 1 ml of transformation mix. Data are representative of three biological replicates with similar results.*

*^b^NT, not tested.*

*^c^After 24 h of incubation, < 15 colonies were obtained which were variable in size, dull, and underneath agar. These colonies remained very tiny upon streaking. They were unstable and could not be recovered after storage.*

*^d^< 50 colonies were obtained after 48 h of incubation. They were unstable and could not be recovered after storage.*

EzrA(*Bsu*) is conditionally essential in the presence of certain antibiotics (Gamba et al., 2015). Consequently, we repeated the transformation assay with an amplicon containing a Δ*ezrA*::P_c_-*erm* deletion–insertion null mutation into the D39 Δ*cps Spn* strain, selecting for erythromycin resistance. We obtained no Δ*ezrA*::P_c_-*erm* transformants after 24 of incubation (data not shown), similar to the results for the Δ*ezrA*<>*aad9* amplicon (Table 1). Finally, we repeated the transformation assay for the Δ*ezrA*<>*aad9* amplicon into laboratory strain R6 (strain EL59), which has acquired numerous mutations compared to its D39W progenitor strain (Lanie et al., 2007). Mutations in the R6 strain genetic background suppress the essentiality of numerous genes involved in PG synthesis, including Δ*mreC*, Δ*gpsB*, Δ*mltG*, and Δ*stkP* (Land and Winkler, 2011; Rued et al., 2017; Straume et al., 2017). However, transformation of the Δ*ezrA*<>*aad9* amplicon into strain R6 gave no colonies after 20 h of incubation (data not shown), similar to the D39 Δ*cps* strain (Table 1). Attempts to delete *ezrA* in the commonly used Rx1 background were also unsuccessful (Massidda et al., 2013). Together, these results show that EzrA(*Spn*) is essential and not conditionally lethal.

To determine phenotypes caused by EzrA*(Spn)* depletion, we constructed a Δ*ezrA*//P_Zn_-*ezrA*^+^ merodiploid strain (IU8799) by moving a Δ*ezrA*<>*aad9* deletion into a *ezrA*^+^//P_Zn_-*ezrA*^+^ strain (IU8795) containing an ectopic copy of *ezrA*^+^ under control of a Zn^2+^-inducible promoter in the *bgaA* gene (see Supplementary Table 1). Consistent with EzrA(*Spn*) essentiality, transformation of the *ezrA*^+^//P_Zn_-*ezrA*^+^ merodiploid with the Δ*ezrA*<>*aad9* amplicon only resulted in many colonies when Zn (0.5 mM ZnCl_2_ and 0.05 mM MnSO_4_) was added to selection plates (Table 1, middle columns). Without added Zn, only a few putative suppressor mutants were detected. Likewise, transformation of a comparable *ftsZ*^+^//P_Zn_-*ftsZ*^+^ merodiploid with a Δ*ftsZ*::*aad9* amplicon only resulted in colonies when Zn was added (Table 1, right columns).

### EzrA Is Required for Normal Cell Growth, Size, and Shape Homeostasis in *Spn*

**FIGURE 2:**
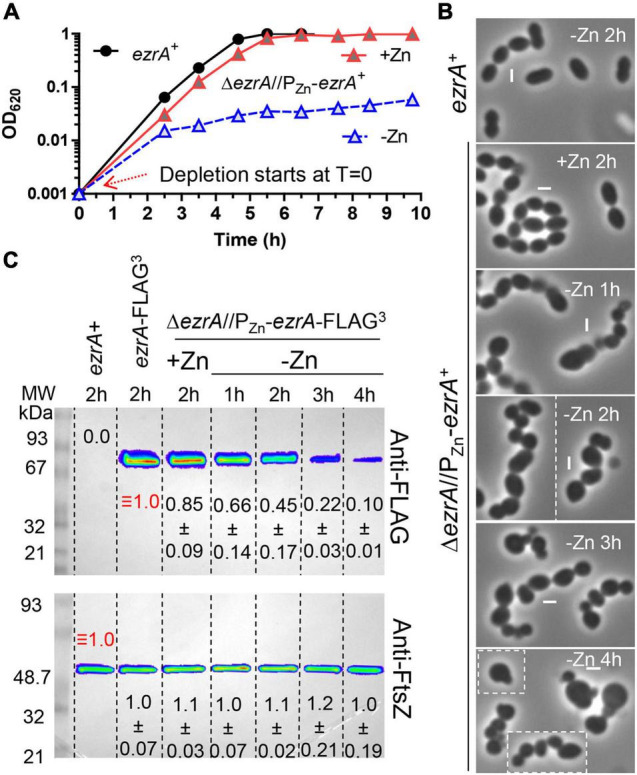
Depletion of EzrA results in severe growth, size, and shape defects. EzrA depletion in untagged-strain, IU8799 (Δ*ezrA*//*bgaA*::P_Zn_-*ezrA*^+^) in **(A)** and **(B)** or tagged-strain, IU9572 (Δ*ezrA*//*bgaA*::P_Zn_-*ezrA*-FLAG^3^) in **(C)**. **(A)** Representative growth curves after resuspension in BHI broth containing or lacking ZnCl_2_ show EzrA-depletion results in bacteriostatic-like growth. IU8799 and IU1945 (WT parent) were grown exponentially in BHI broth supplemented with 0.5 mM ZnCl_2_ and 0.05 mM MnSO_4_ to OD_620_ ≈ 0.1, spun, and resuspended to OD_620_ ≈ 0.001 for this growth curve. **(B)** Representative PCm images of wild-type (IU1945) or EzrA depletion strain (IU8799) after resuspension in BHI broth supplemented with, or lacking supplemented ZnCl_2_ for 1, 2, 3, or 4 h. Resuspension volumes were adjusted in BHI broth supplemented with or without 0.5 mM ZnCl_2_ and 0.05 mM MnSO_4_ to OD_620_ ≈ 0.36 (for *T* = 1 h time-point) or 0.012 (for *T* = 2, 3, and 4 h time-points). Experiments were performed three times with similar results. See Supplementary Figure 8 for quantitation. **(C)** Representative western blots of gels containing extracts of IU1945 (wild-type), IU5456 (*ezrA*-FLAG^3^), or IU9572 (Δ*ezrA*//P_Zn_-*ezrA*-FLAG^3^). Western blotting using anti-FLAG antibody or anti-FtsZ are described in section “Materials and Methods” and Supplementary Materials. Protein amounts of ZnCl_2_-induced and ZnCl_2_-depleted strains relative to IU5456 (*ezrA*-FLAG^3^ native) for EzrA-FLAG^3^ levels or IU1945 (untagged parent isolate) for FtsZ, levels were quantified and are from at least 3 independent biological replicates experiments (± standard deviations).

To characterize EzrA depletion phenotypes, we performed growth curve analyses and PCm on cells depleted for EzrA in BHI broth, as described in section “Materials and Methods.” Growth rates and yields of an EzrA merodiploid (Δ*ezrA*//P_Zn_-*ezrA*^+^) strain were dependent on the concentration Zn (ZnCl_2_ + 1/10 MnSO_4_) in cultures, where Mn^2+^ was added to prevent Zn^2+^ toxicity (see section “Materials and Methods”; Figure 2A and Supplementary Figure 7A; Jacobsen et al., 2011). Depletion growth curves depended on OD_620_ at which Zn was omitted from cultures containing 0.5 mM Zn (Supplementary Figure 7B), consistent with the requirement of EzrA for growth. Although cultures containing 0.3–0.5 mM Zn grew, similarly (Supplementary Figure 7A), only 0.5 mM fully complemented cell shape and was used in depletion experiments started at OD_620_ ≈0.01–0.05 (Supplementary Figures 7B,C). Finally, a merodiploid strain ectopically expressing EzrA fused to a FLAG^3^ epitope tag (Δ*ezrA*//P_Zn_-*ezrA*-FLAG^3^) grew, similarly, to the Δ*ezrA*//P_Zn_-*ezrA*^+^ strain in 0.5 mM Zn and after Zn was removed, after which OD_620_ stopped increasing in ≈3 h (Supplementary Figure 7C).

After depletion of EzrA or EzrA-L-FLAG^3^ for 1 h or longer, cell shapes became distorted and non-uniform (Figure 2B). With time, the median aspect ratio of EzrA-depleted cells decreased compared to the non-depleted control cells, and cells appeared as irregular spheroids with variable relative volumes (Figure 2B and Supplementary Figure 8). Quantitative Western blotting showed that ectopic EzrA-L-FLAG^3^ in the presence of 0.5 mM Zn was expressed at 85% of the cellular amount of EzrA-L-FLAG^3^ expressed from the native chromosomal locus (Figure 2C). Depletion of EzrA-L-FLAG^3^ for 1 to 4 h reduced the relative cellular amount to 66% and to 10%, respectively (Figure 2C). At 3 h of depletion, when cultures stopped growing (Figure 2A and Supplementary Figure 7C), the relative amount of EzrA-L-FLAG^3^ was ≈22% of that in cells expressing EzrA-L-FLAG^3^ from the native locus (Figure 2C). Distorted cells depleted of EzrA did not appear to lyse, and cell debris was not observed by PCm. Viability staining with the LIVE/DEAD *Bac*Light procedure, as described in *Materials and Methods*, showed that > 90% of non-growing, distorted cells lacking EzrA were stained as viable for 3, 4, and 7 h after depletion (Supplementary Table 3). Consistent with this conclusion, CFUs of Δ*ezrA*//P_Zn_-*ezrA* cells depleted of EzrA for 4 or 8 h without Zn were recovered on plates containing Zn with no decrease or increase in CFU/mL throughout the depletion time course (data not shown). Thus, EzrA is required for *Spn* cell size and shape homeostasis, although prolonged depletion of EzrA under these culture conditions is bacteriostatic and not bactericidal.

Finally, we determined the effects of the EzrA(ΔTM) and EzrA(ΔQNR motif) or EzrA(QND motif) mutant variants, which mediate membrane anchoring and medial localization, respectively, in *Bsu* (Supplementary Figures 1C, 9A) (Land et al., 2014). We expressed EzrA(ΔTM) (deletion of amino acids 2-26; strain IU11123), EzrA(ΔQNR) (deletion of amino acids 510-516 (deletion of amino acids 510-516; strain 10909), and EzrA(QND motif) (*ezrA*(R515D); strain IU10901) from the native locus of a merodiploid strain ectopically expressing *ezrA*^+^ from the P_Zn_ promoter (Supplementary Table 1). Depletion of EzrA^+^ showed that *ezrA*(ΔTM) and *ezrA*(ΔQNR) caused the same growth defects as Δ*ezrA* (Supplementary Figure 9B), whereas *ezrA*(QND) strains grew like WT (data not shown). sfGFP fused to the C-terminus of EzrA(QND), EzrA(ΔQNR), or EzrA(ΔTM) in comparable EzrA^+^ merodiploid strains showed that EzrA(QND)-sfGFP and EzrA(ΔQNR)-sfGFP were expressed at nearly the same level as EzrA^+^-sfGFP upon EzrA^+^ depletion, whereas EzrA(ΔTM)-sfGFP was expressed at only 30% of the WT level (Supplementary Figure 9C). Moreover, the ectopic expression of EzrA(ΔTM)-FLAG^3^ at the WT level did not complement a Δ*ezrA* mutation, confirming that the TM domain is required for EzrA function (data not shown). EzrA(ΔQNR)-sfGFP and EzrA(ΔTM)-sfGFP were highly mislocalized upon depletion of EzrA^+^ in merodiploid strains (Supplementary Figure 9D). Remaining midcell rings containing EzrA(ΔQNR)-sfGFP or EzrA(ΔTM)-sfGFP may reflect dimer formation with residual EzrA^+^ after depletion. In contrast, EzrA(QND motif)-sfGFP largely localized normally to midcell and equatorial bands in irregularly shaped cells (Supplementary Figure 9D). In addition, the *ezrA*(QND)//P_Zn_-*ezrA*^+^ mutant was temperature sensitive for growth at 42°C in the absence of Zn (data not shown). We conclude that the TM domain of EzrA is required for membrane localization and possibly protein stability, while the QNR motif localizes EzrA to the septal and equatorial rings of *Spn* cells. While not necessary for ring localization, amino acid R515 in the QNR motif of EzrA(*Spn*) is required for full function.

### EzrA Is Required for Normal Chromosome Segregation in *Spn*

Depletion of EzrA often results in larger cells attached to smaller cells (Figure 2B), reminiscent of minicells containing guillotined DNA or no DNA produced by division site placement mutants (Wu and Errington, 2004; Bernhardt and de Boer, 2005). To test this idea, we stained the DNA of cells from WT, EzrA-complemented, or EzrA-depleted cultures with DAPI as described in *Materials and Methods.* We counted nucleated and anucleate cells that were at pre-divisional (stage 1) or post-divisional (stage 4) stages (*n* = 400 per condition) based on PCm (Supplementary Figure 10). Depletion of EzrA resulted in a relatively high number (3.25% of cells detected by PCm) of cells lacking nucleoids compared to WT (none) or EzrA-complemented strains (<0.25%) (arrows, Supplementary Figure 10; Supplementary Table 4). By comparison, cells lacking MapZ, which plays a role in positioning FtsZ-rings at the equators of daughter cells (Fleurie et al., 2014a; Holeckova et al., 2015; Perez et al., 2019), displayed only about 0.5% anucleate cells, which was greater than WT but far fewer than in EzrA-depleted cells (Supplementary Table 4). These results indicate that EzrA plays an important role in chromosome segregation, likely by modulating Z-ring placement and organization, analogous to Z-ring regulators in rod-shaped bacteria.

### EzrA Is Required for the Midcell Placement of FtsZ-Rings in *Spn*

**FIGURE 3:**
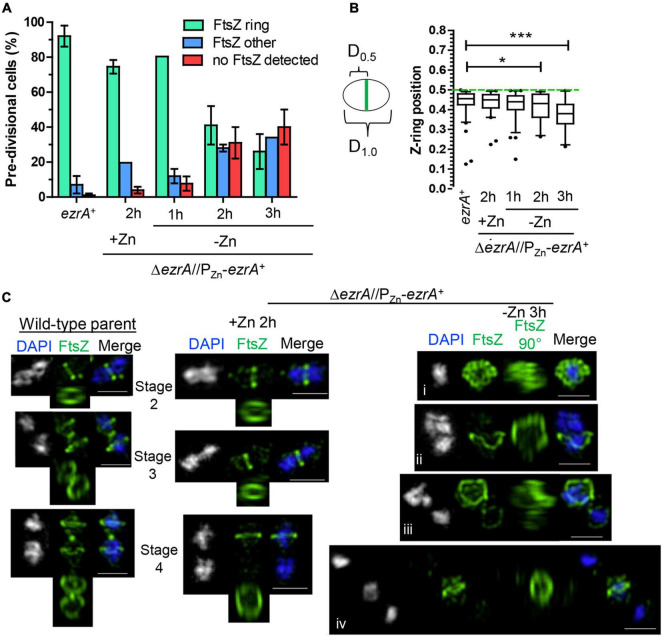
EzrA depletion results in cells lacking Z-rings or aberrations in Z-ring positions. Wild-type (IU1945; *ezrA*^+^) and *ezrA* depletion strain (IU8799+Zn; Δ*ezrA*//*bgaA*::P_Zn_-*ezrA*^+^) were grown exponentially (+Zn; 0.5 mM ZnCl_2_ and 0.05 mM MnSO_4_), and IU8799 was depleted of EzrA by shifting cells to BHI broth lacking ZnCl_2_ and MnSO_4_ as described in *Materials and Methods.* EzrA depleted cells were obtained at appropriate time points, prepared for IFM, and analyzed. Native FtsZ proteins were detected *via* anti-FtsZ antibodies as described in Supplementary Table 5. **(A)** Pre-divisional cells (stage 1) or post-divisional cells (daughters of stage 4) were binned into three different categories based on FtsZ localization events from 2D-EFm images. The bar is the mean percentage ± the SD for two separate IFM fields of one biological replicate. The different color bars represent cells that contained FtsZ-rings (green), FtsZ other (blue), or no FtsZ detected (red). The experiment was performed two independent times with similar results. **(B)** Box-and-whisker plots (whiskers, 5 and 95 percentile) of the FtsZ-ring position relative to whole cell length in cells which contained rings in **(A)**. The smaller distance of the Z-ring to the cell pole was measured and divided by the whole cell length to determine Z-ring position. Green dotted line indicates *D* = 0.5, a precisely midcell measured Z-ring. Data are from one biological replicate for a subset of cells that contained rings in **(A)**. (*ezrA*^+^, *n* = 86 cells; +Zn 2 h, *n* = 65 cells; -Zn 1 h, *n* = 72 cells; -Zn 2 h, *n* = 35 cells; -Zn 3 h, *n* = 24 cells). *p*-values obtained with Mann–Whitney two-tailed unpaired t-test are for comparisons between *ezrA*^+^ and other conditions. * and *** indicate *p* < 0.05 and < 0.001, respectively. **(C)** 3D-SIM examples of FtsZ-labeled cells shows EzrA-depletion leads to major aberrances in FtsZ-ring presence, FtsZ-ring placement, and FtsZ-structure. More than 20 cells were analyzed per condition (wild-type, Δ*ezrA*//P_Zn_-*ezrA*^+^ +Zn, Δ*ezrA*//P_Zn_-*ezrA*^+^ -Zn 3 h). Left panels show wild-type cells. Middle panel shows EzrA complemented cells. Further right panels show different aberrances in FtsZ structures during EzrA depletion, (i) FtsZ diffuse, (ii) FtsZ aberrant twisted rings, (iii) aberrant stage 4 like cells, (iv) two outer cells without labeling containing intense DAPI staining while the middle cell contains semi-diffuse DAPI and normal Z-ring.

EzrA regulates FtsZ-ring number and position in *Bsu* cells. In the absence of EzrA, *Bsu* cells contain two extra parallel Z-rings per cell length (Levin et al., 1999). To determine the role of EzrA in regulating the presence and position of FtsZ-rings in *Spn*, we performed 2D-IFM using the anti-FtsZ(*Spn*) antibody in EzrA-depleted cells (Lara et al., 2005). The majority of pre-divisional (stage 1) cells of WT, EzrA-complemented (+Zn), and EzrA-depleted (1 h) cells contained normally placed FtsZ-rings at midcell (see Figures 1A, 3A). In contrast, after 2 h of EzrA depletion, the majority of cells lacked identifiable Z-rings (Figure 3A). Cells classified as “FtsZ other” did not contain obvious Z-rings but did show the presence of FtsZ-labeling, and cells classified as “no FtsZ detected” lacked FtsZ labeling and appeared opaque by PCm, which may indicate an inability to be permeabilized and take up antibodies (data not shown). Nevertheless, comparison of cells with the FtsZ signal indicates that at 2 or 3 h after EzrA depletion, less than half of pre-divisional cells have Z-ring structures, while 90% of WT and 70% of EzrA-complemented cells contain Z-rings (Figure 3A), indicating that EzrA is necessary for FtsZ-ring formation in *Spn.* 3D-SIM IFM confirmed this conclusion at higher resolution than 2D-IFM. Among the patterns of FtsZ labeling in cells depleted for EzrA for 3 h, we observed diffuse localization and aberrant, twisted Z-ring structures, as well as cells lacking labeling patterns (Figure 3C).

To determine if EzrA influences positioning of FtsZ-rings in *Spn*, we determined the relative position of Z-rings in cells from Figure 3A. We measured the distance of Z-rings to the nearest cell pole normalized to cell length, where 0.5 indicates exact midcell placement. Fifty percent of WT and EzrA-complemented (+Zn) cells had FtsZ-rings positioned between 0.45 and 0.5 (Figure 3B). Cells depleted of EzrA for 1 or 2 h maintained Z-rings near this midcell range. By 3 h of EzrA depletion, the median (0.38) and distributions started to trend downward. Z-rings were significantly out of the midcell region at 3 h, indicating that the presence of EzrA also influences placement of Z-rings in *Spn.*

**FIGURE 4:**
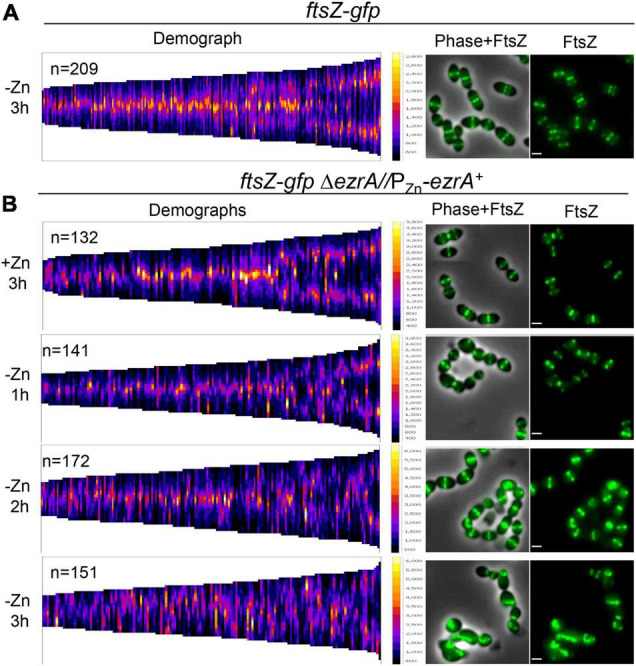
Severe defects in FtsZ-GFP localization upon EzrA depletion in live cells. FtsZ-GFP was localized in live wild-type cells [*ftsZ-gfp*; IU8845 in **(A)**] or live EzrA depletion strain [*ftsZ-gfp*Δ*ezrA//*P_Zn_*-ezrA^+^;* IU8908 in **(B)**] grown and visualized in BHI broth at OD_620_≈0.05–0.15 using EFm. EzrA depletion strain was complemented (+Zn; 0.5 mM ZnCl_2_ and 0.05 mM MnSO_4_). Alternatively, EzrA-depleted cells were visualized at 1, 2, or 3 h, upon removal of supplemented Zn/Mn from the BHI broth. Demographs assembled in MicrobeJ (Ducret et al., 2016) showing FtsZ-GFP intensity (left panels) and representative fields (right panels) are shown. Data are from two independent biological replicates in which (*n*) number of cells were analyzed.

We confirmed these conclusions by tracking FtsZ-GFP expressed from the native chromosomal locus in live cells complemented or depleted for EzrA (Figure 4 and Supplementary Figure 11). Defects in midcell localization and correct plane placement of FtsZ-GFP were apparent by 1 h of EzrA depletion (Figure 4B and Supplementary Figure 11B). By 3 h of EzrA depletion, polar placement of FtsZ-GFP band structures or condensed foci were observed (Figure 4B and Supplementary Figure 11C). Demographs of FtsZ-GFP in a population of EzrA-depleted cells revealed major delocalization of FtsZ-GFP as a function of relative cell length (Figure 4B). Localization of FtsZ-GFP to equatorial rings became aberrant at 2 h of EzrA depletion, while FtsZ-GFP localization in the whole population became aberrant at 3 h (Supplementary Figure 11B). We also observed that the culture density of strains expressing FtsZ-GFP began to drop after about 4 h of EzrA depletion, in contrast to strains expressing FtsZ^+^, indicating an aberration caused by the FtsZ-GFP fusion upon extended EzrA depletion (Supplementary Figure 11A). We conclude from these combined IFM and GFP imaging experiments that EzrA is required for the presence and localization of FtsZ in *Spn*, in contrast to *Bsu* where the absence of EzrA leads to the formation of additional FtsZ-rings (Levin et al., 1999).

### EzrA Is Required for the Midcell Peptidoglycan Synthesis in *Spn*

**FIGURE 5:**
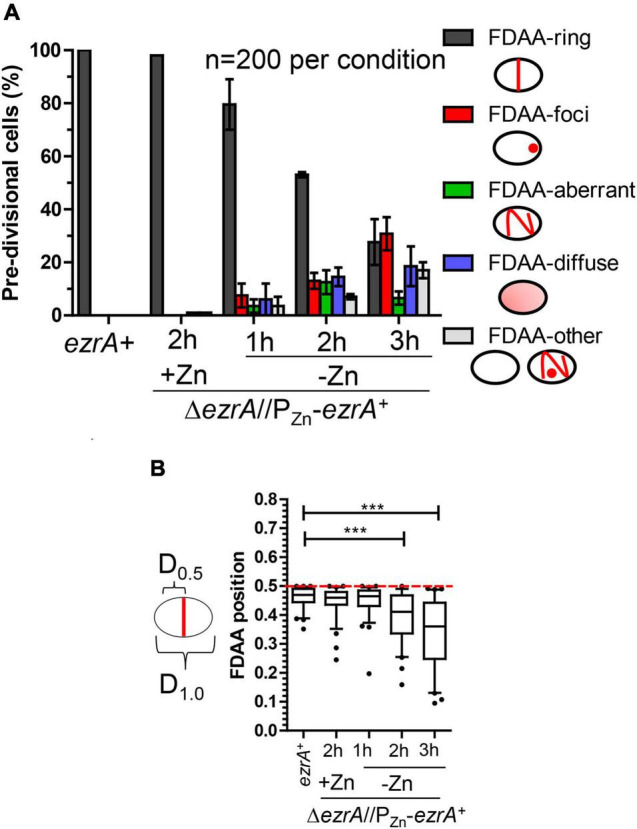
Quantification of 2D-FDAA labeling patterns of EzrA depleted pre-divisional cells which were pulse-chase labeled. Strains used IU1945 (wild-type cells) and IU8799 (Δ*ezrA//*P_Zn_*-ezrA^+^*). Cells were grown and labeled as described in Supplementary Figure 12A. **(A)** Categorical bar graph showing different labeling characteristics with the short-pulse FDAA (TADA). Average values are represented by the colored bars, and error bars are for a SD of two different biological replicates in which 100 cells were counted per experiment. **(B)** Box-and-whisker plots (whiskers, 5 and 95 percentile) showing the position of FDAA-rings relative to whole cell length were determined in cells which contained FDAA-rings in **(A)**. Red dotted line indicates *D* = 0.5, a precisely midcell measured Z-ring. *p*-values obtained with Mann–Whitney two-tailed unpaired *t*-test are for comparisons between *ezrA*^+^ and other conditions. *** indicate *p* < 0.001.

In pre-divisional and early divisional *Spn* cells, PG synthesis occurs at a midcell ring initially organized by FtsZ and FtsA (Supplementary Figure 1) (see Briggs et al., 2021). As septation proceeds, FtsZ continues to organize the inner ring of sPG synthesis that closes the septum, while pPG remains in an outer ring that lacks FtsZ (Briggs et al., 2021; Perez et al., 2021). Given that FtsZ organizes PG synthesis at different stages of division in *Spn*, we predicted aberrant PG synthesis patterns upon depletion of EzrA that mirrored those of FtsZ described above (Figures 3, 4, and Supplementary Figure 11). To detect regions of active PBP TP activity, we labeled cells sequentially with two colors of FDAAs (Supplementary Figure 12A). Cells were labeled with a “long pulse” of HADA FDAA at the start of EzrA depletion or continued synthesis in merodiploid strains, as described in *Materials and Methods*. After 1, 2, or 3 h, cells were washed and a second TADA FDAA was added for a short pulse (5–18 min depending on strains or conditions, Supplementary Figure 12A), indicating regions of new PG synthesis. Cells observed by PCm and EFm showed patterns of TADA labeling (Figure 5A and Supplementary Figures 12A–C) similar to those of FtsZ with increasing EzrA depletion (Figure 3A). At 1 h of EzrA depletion, most TADA labeling was at midcell rings of pre-divisional and post-divisional cells, but by 2 h and beyond of EzrA depletion, TADA labeling was increasingly seen in polar foci and other aberrant and diffuse patterns (Figure 5A). 3D-SIM images captured details of these aberrant PG synthesis patterns, notably rings or puncta of TADA labeling at distal poles, diffuse TADA labeling, and aberrantly placed planes of TADA labeling in EzrA-depleted cells (Supplementary Figures 13B,C). As expected from these results, the placement of medial TADA-rings, when present, showed the same trend as FtsZ-rings in being located away from the exact midcell the longer EzrA was depleted (Figures 3B, 5B). Together, these results indicate that PG synthesis tracks with the mislocalization of FtsZ that occurs when EzrA is depleted, possibly contributing to the diverse, aberrant cell shapes that occur.

### Overexpression of EzrA Leads to Extra Z-Rings in *Spn*

**FIGURE 6:**
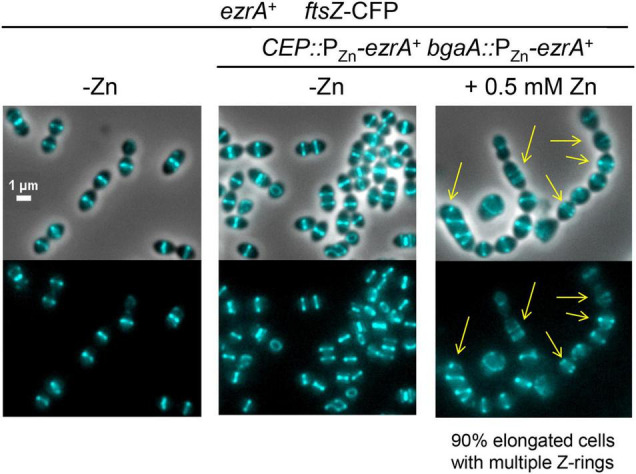
Overexpression of EzrA leads to extra Z-rings in cells expressing FtsZ-CFP in BHI. FtsZ-CFP was localized in otherwise wild-type cells (IU13406) or *ezrA* merodiploid strain (IU13700; *ftsZ-cfp ezrA*^+^//CEP::P_Zn_-*ezrA*^+^//*bgaA*::P_Zn_-*ezrA*^+^) in BHI 37°C 5% CO_2_. Cells were grown from OD_620_≈0.003 without supplemented ZnCl_2_ (- Zn) or supplemented with ZnCl_2_ (0.5 mM ZnCl_2_ and 0.05 MnSO_4_) to overexpress *ezrA*^+^. Yellow arrows point to cells with extra Z-rings. Images were obtained after 4 h of growth in the presence of ZnCl_2_/MnSO_4_. Ninety percent of the cell population of EzrA-overexpressed cells were elongated and contained multiple Z-rings.

The essentiality of EzrA(*Spn*) and the dependence of FtsZ-ring formation on EzrA (Figures 3, 4) suggest that EzrA(*Spn*) is a positive regulator of FtsZ-ring formation, unlike EzrA(*Bsu*) which is a negative regulator (Levin et al., 1999). To test this hypothesis, we overexpressed EzrA to see if extra FtsZ-rings appear in *Spn.* To this end, we introduced copies of P_Zn_-*ezrA*^+^ into two different ectopic sites in an *Spn* strain containing *ftsZ*-CFP at the native chromosomal locus (strain IU13700). In the BHI medium without added Zn, FtsZ-CFP localized normally in the *ftsZ*-CFP or *ezrA*^+^ merodiploid strain (-Zn, Figure 6), whereas overexpression of EzrA resulted in 90% of cells showing elongated, wider chains of cells containing multiple FtsZ-CFP rings (arrows, Figure 6). We corroborated this conclusion using a different construct grown in the C+Y medium. FtsZ-sfGFP was expressed at the native locus in a merodiploid strain containing an ectopic copy of P_Zn_-*ezrA*^+^ (strain IU14224). The *ftsZ-sfgfp* control strain with or without Zn and the *ftsZ-sfgfp//*P_Zn_-*ezrA*^+^ merodiploid strain without Zn showed the expected pattern of FtsZ-rings (Supplementary Figure 14). Overexpression of EzrA by the addition of 0.25 or 0.5 mM Zn again caused formation of chains of elongated, wider cells containing multiple FtsZ-sfGFP rings (arrows, Supplementary Figure 14). Note that in the C+Y medium, the addition of 0.5 mM Zn and 0.05 mM Mn led to cell debris, indicative of lysis in both *ezrA*^+^ and EzrA-overexpression cells. However, multiple FtsZ-sfGFP rings were only present in EzrA-overexpression cells and not in WT *ezrA*^+^ cells. These combined experiments confirm that the appearance of multiple FtsZ-rings upon EzrA overexpression is not tag, construct, or medium dependent. We conclude that overexpression of EzrA in *Spn* does indeed leads to increased formation of parallel FtsZ-rings, consistent with EzrA acting as a positive regulator of FtsZ-ring formation in *Spn* cells.

### FtsZ Depletion Results in Enlarged, Dead Spherical Cells That Are Subject to LytA-Dependent Autolysis

**FIGURE 7:**
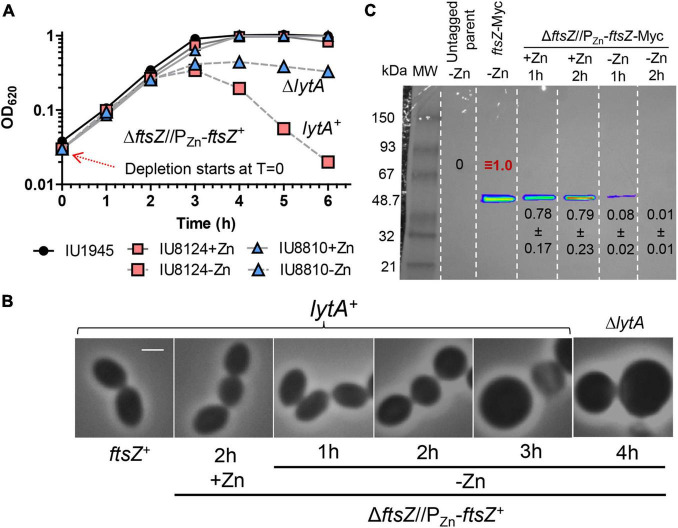
Depletion of FtsZ results in enlarged spherical cell morphology followed by LytA induced lysis. Strains IU1945 (wild-type parent), IU8124 (Δ*ftsZ*//P_Zn_-*ftsZ*^+^), IU8237 (Δ*ftsZ*//P_Zn_-*ftsZ*-Myc), and IU8810 (Δ*ftsZ*//P_Zn_-*ftsZ* Δ*lytA*) were grown exponentially in BHI broth supplemented with 0.3 mM ZnCl_2_ and 0.03 mM MnSO_4_ to OD_620_ ≈ 0.1, spun, and resuspended in BHI broth supplemented with or without 0.3 mM ZnCl_2_ and 0.03 mM MnSO_4_. **(A)** Representative growth curves after resuspension in BHI broth at OD_620_ ≈ 0.012 containing or lacking ZnCl_2_. Red arrow indicates when depletion of ZnCl_2_ started. **(B)** Representative images of wild-type (IU1945) or FtsZ depletion strains (IU8124; *lytA*^+^ and IU8810; Δ*lytA*) after resuspension in BHI broth supplemented with, or lacking ZnCl_2_ for 1, 2, 3, and 4 h. Resuspension OD_620_ ≈ 0.36 (for *T* = 1 h time point) or 0.012 (for *T* = 2, 3 or 4 h time point). The experiment was performed at least two separate times with similar results. Scale bar = 1 μm. See Supplementary Figure 15B for cell size measurements and statistical analysis. **(C)** Representative Western blot membranes containing extracts of IU1945 (untagged parent), IU7223 (*ftsZ*-Myc), or IU8237 (Δ*ftsZ*//P_Zn_-*ftsZ*-Myc) mutant strains. Western blotting using the anti-Myc antibody is described in *Materials and Methods*. Protein amounts of ZnCl_2_-induced and ZnCl_2_-depleted strains relative to IU7223 (*ftsZ*-Myc expressed from the native promoter) were determined and quantified as detailed in section “Materials and Methods” and are from either two or three independent biological replicate experiments (± SD).

To study the relationship between FtsZ and EzrA in *Spn*, we further characterized the phenotypes caused by FtsZ depletion under our culture conditions. We constructed a strain expressing FtsZ-Myc from the chromosomal locus (IU7223) and a Δ*ftsZ* merodiploid strain expressing FtsZ^+^ or FtsZ-Myc from an ectopic site (IU8124 or IU8237, respectively). Induction of FtsZ^+^ or FtsZ-Myc with 0.3 mM Zn fully complemented the Δ*ftsZ* mutation in the merodiploid strains for growth and cell morphology (Figures 7A,B, and Supplementary Figures 15A,B). Western blotting indicated that ectopic FtsZ-Myc was expressed at 80% in the merodiploid compared to FtsZ-Myc expressed from the chromosome (Figure 7C). Depletion of FtsZ^+^ or FtsZ-Myc resulted in a rapid cessation of growth, rounding of cells to spheroids, enlargement of relative cell volumes (≈ 4 ×), and a decrease in OD_620_ indicative of cell autolysis (Figures 7A,B and Supplementary Figures 15A,B). These phenotypes are similar to the aberrant, heterogeneous, exploding cells reported previously for CRIPSRi- or IPTG-regulated depletion of FtsZ in strain D39V (Liu et al., 2017; Gallay et al., 2021). Western blotting indicated that FtsZ-Myc cellular amount was reduced rapidly to < 10% within 1 h of depletion (Figure 7C). Titration with 0.135 mM Zn showed the relative expression of FtsZ-Myc at 3 h of growth to be at ≈23%, which was sufficient to allow continued growth without lysis (data not shown). Autolysis, but not cell shape defect upon FtsZ depletion, was abrogated by a Δ*lytA* mutation, indicating that autolysis of spheroidal lacking FtsZ was mediated by induction of the LytA amidase activity (Figure 7A). Nevertheless, CFU counting showed that the viability of the spheroid FtsZ-depleted Δ*lytA* cells decreased at a similar rate as in the *lytA*^+^ cells that were lysing (Supplementary Figure 15A). In FtsZ-depleted *lytA*^+^ and Δ*lytA* cells, CFU/mL counts at 7 h of depletion dropped to ≈1% to 3% compared to CFU counts at 1 to 3 h of depletion. We concluded that, as expected, FtsZ depletion in *Spn* is lethal and results in LytA-induced autolysis and loss of viability that is independent of LytA autolysis.

### EzrA Recruitment to Equatorial Rings and Organized Peptidoglycan Synthesis Require FtsZ

**FIGURE 8:**
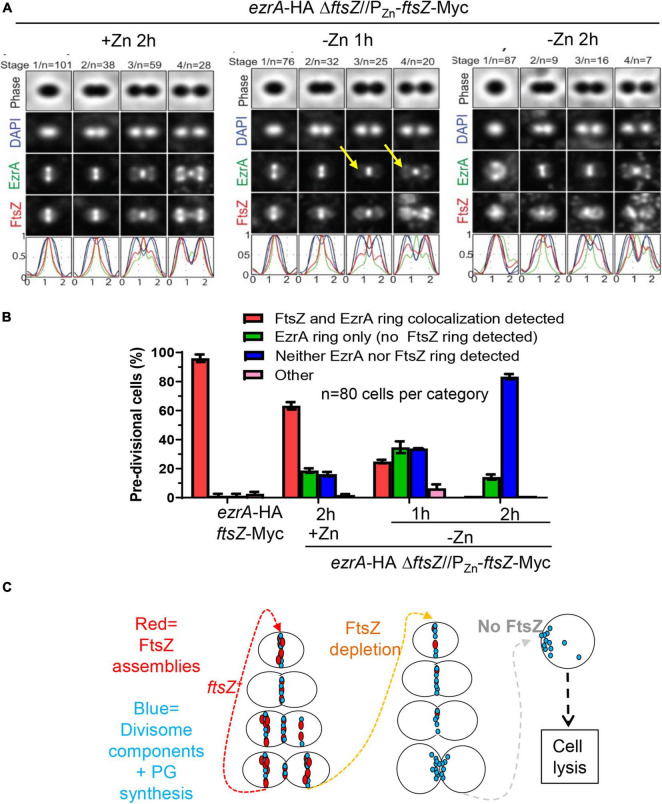
Recruitment of EzrA-rings to equators of future dividing cells is dependent on FtsZ. Wild-type parent strain IU7223 (*ftsZ*-Myc *ezrA*-HA) and FtsZ depletion strain IU8237 (*ezrA-*HA Δ*ftsZ*//*bgaA*::P_Zn_-*ftsZ*-Myc) were grown exponentially, and IU8237 was depleted of FtsZ-Myc by shifting cells to BHI broth not supplemented with additional ZnCl_2_ and MnSO_4_ as described in section “Materials and Methods.” Cells were obtained at appropriate time intervals and prepared for IFM as described in *Materials and Methods.* Data are from two independent biological replicates. **(A)** Averaged images with fluorescence intensity traces showing FtsZ-Myc and EzrA-HA localization during FtsZ-Myc depletion in IU8237 (*ezrA*-HA Δ*ftsZ*//P_Zn_-*ftsZ*-Myc). Cells were binned into division stages 1-4, and images from the indicated number of cells (*n*) from at least two independent biological replicates were averaged using IMA-GUI program as described in section “Materials and Methods.” (i) Row 1, cell shapes determined from phase-contrast images; row 2, nucleoid locations from DAPI labeling; row 3, EzrA-HA locations from IFM; row 4, FtsZ-Myc locations from IFM; row 5, normalized mean fluorescence intensity distributions along the horizontal cell axis for each channel (black, phase image; blue, DNA; green, EzrA; red, FtsZ). Arrows indicate equatorial ring where EzrA is recruited in +Zn condition but less in -Zn conditions. **(B)** Bar graph quantifying FtsZ-Myc and EzrA-HA-ring co-occurrence during FtsZ-Myc depletion. Bars are the averages of two separate biological replicates while error bars are the SD. **(C)** Schematic for the orchestration of divisome components (turquoise) by FtsZ (red). FtsZ is required to assemble division sites. In the absence of FtsZ, pre-formed divisome rings can still exist but recruitment of the divisome to equators of future dividing daughter cells ceases to exist. Diffuse unorganized PG synthesis at old division sites still occurs, which leads to enlargement of cells, cell death, and eventual cell lysis.

We determined the organization of EzrA upon FtsZ depletion in a doubly epitope-tagged merodiploid strain expressing EzrA-HA from the native chromosomal locus and FtsZ-Myc ectopically (strain IU8237). We averaged 2D-EFm IFM images of dually labeled cells at different stages of division (Figure 8A). After 1 h of FtsZ depletion, FtsZ localization started to become disorganized, while EzrA-rings remained at midcell septa but failed to appear at the equators of daughter cells in stage 3 and 4 divisional cells (arrows, Figure 8A), compared to WT cells (in strain IU7223; see Supplementary Figure 2B). After 2 h of FtsZ depletion, a band of EzrA remained at septa and only weakly appeared at equators compared to EzrA-complemented (+Zn) cells, while FtsZ became severely disorganized (Figure 8A and Supplementary Figure 16). 3D-SIM images confirmed these conclusions (Supplementary Figure 17). Although some cells were able to complete a cycle of division and chromosome segregation, EzrA remained at septa in rings or diffuse areas and was not recruited to the equators of daughter cells (bottom, Supplementary Figure 17). Enlarged, spheroidal cells depleted of FtsZ contained diffuse regions of nucleoid staining, consistent with defects in chromosome organization and segregation (Supplementary Figure 17). This lack of EzrA-rings at future division sites supports the conclusions that FtsZ is required for initial recruitment of EzrA to equatorial rings of daughter cells.

Although EzrA was absent from equatorial rings during FtsZ depletion, many pre-divisional cells still contained EzrA-rings but lacked FtsZ labeling, which was obscured by the image averaging used in Figure 8A. To circumvent this issue, we counted co-localization events of EzrA-rings and FtsZ-rings during FtsZ depletion in pre-divisional cells (Figure 8B). WT cells displayed Z-ring and EzrA-ring colocalization in nearly all cells, whereas FtsZ-complemented strains (+Zn) showed ≈60% EzrA and FtsZ co-localization in pre-divisional cells (Figure 8B), consistent with the slightly lower expression of FtsZ detected in Western blots (Figure 7C). Depletion of FtsZ for 1 or 2 h showed a large, increasing drop of EzrA-ring and FtsZ-ring colocalization (Figure 8B). Concomitantly, after severe depletion of FtsZ for 2 h, a majority (≈80%) of cells lacked both EzrA- and FtsZ-rings (Figure 8B). In addition, we examined the localization pattern of FtsA upon FtsZ depletion since FtsA, similar to EzrA, co-localizes with FtsZ at all stages of cell division (Mura et al., 2016). Co-localization experiments of FLAG-FtsA upon FtsZ-Myc depletion led to a similar result (Supplementary Figure 18). Together, these data indicate that once assembled, EzrA- and FtsA-ring structures can persist in the absence of FtsZ-rings, but assembly of new EzrA and FtsA-rings requires the presence of FtsZ (Figure 8C).

Finally, a similar conclusion was reached about the organization of PG synthesis upon FtsZ depletion. Merodiploid Δ*ftsZ*//P_Zn_-*ftsZ*^+^ cells labeled with one color of FDAA for a long pulse to indicate regions of “old” PG were washed and labeled with a short pulse of a second color of FDAA to indicate regions of “new” PG synthesis (Supplementary Figure 19A) and examined by 2D-EFm (Supplementary Figures 19B, 20) or by 3D-SIM (Supplementary Figure 19C). After 1 h of FtsZ depletion, new PG synthesis was still occurring in rings at midcell regions. By 2 h of FtsZ depletion, organized FDAA-rings of new PG synthesis were largely absent (Supplementary Figure 19B), and only aberrant, diffuse FDAA-labeling patterns at old division sites and elsewhere were present in FtsZ-depleted in cells (Supplementary Figures 19C, 20). We conclude that FtsZ is required for organizing PG synthesis at septa and equators of dividing *Spn* cells, but PG synthesis continues in a diffusive manner in the absence of this organization.

### EzrA Interaction Profiles Suggest That EzrA Functions at the Interface Between Z-Ring Regulation and Peptidoglycan Synthesis and Cell Division in *Spn*

**FIGURE 9:**
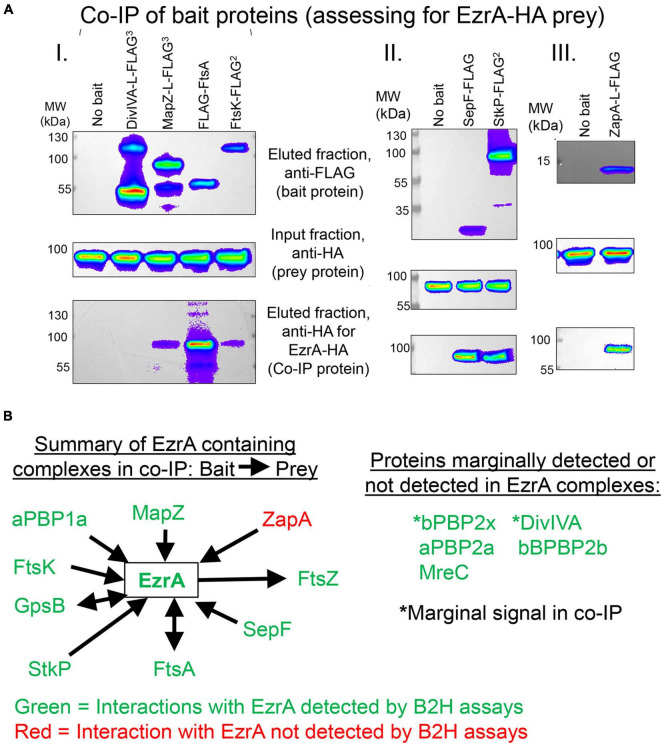
EzrA is in complex with Z-ring regulators and cell-cycle regulators. Associations of EzrA with FtsZ-ring regulators (MapZ, FtsA, SepF, ZapA) and cell-cycle regulators (DivIVA, FtsK, StkP) in *Spn* were tested by co-IP in **(A)** and **(B)** or B2H in **(B)**. Experiments are representative from two independent biological replicates. **(A)** Top panels, labeling of transferred membranes with anti-FLAG in the eluted fractions shows the presence of FLAG-tagged bait protein. Middle panels, labeling with anti-HA in the input fraction shows the presence of EzrA-HA. Bottom panels, labeling with anti-HA in the eluted fraction shows if EzrA-HA was associated with the bait protein. Strains were subjected to Co-IP and Western blotting as described in section “Materials and Methods.” Complete genotypes are listed in Supplementary Table 1. (I) “No bait” (IU9713), DivIVA-FLAG^3^ (IU11414), MapZ-FLAG^3^ (IU11430), FLAG-FtsA (IU11476), FtsK-FLAG^2^ (IU11664. (II) “No bait” (IU6810), SepF-FLAG (IU12076), or StkP-FLAG^2^ (IU12077). (III) “No bait” (IU11939) and ZapA-FLAG (IU11840). **(B)** Summary of EzrA co-IP results and pairwise B2H interaction assays. See Tables 2, 3 for quantitation and tabulation of co-IP results. See Supplementary Figure 21 for B2H results.

Previously, it was shown that pneumococcal EzrA interacts with FtsZ in B2H experiments and in biochemical assays using surface plasmon resonance detection (Fleurie et al., 2014b; Rued et al., 2017). We further determined complexes that contain EzrA in *Spn* cells by co-IP (Figures 1C, 9) and potential direct interactors of EzrA(*Spn*) by B2H assays (Supplementary Figure 21). For Co-IP assays, chromosomal expressed EzrA-L-FLAG^3^ in extracts of cells was the bait that bound magnetic FLAG-tag beads, and an extract from cells expressing untagged EzrA was used as the negative control (Figure 1C). Even without cross linking, EzrA pulled down FtsZ, indicative of a complex containing FtsZ and EzrA. B2H assays confirmed direct binding between EzrA and FtsZ in both B2H constructs (Supplementary Figure 21; Fleurie et al., 2014b; Rued et al., 2017).

EzrA was reported to interact with several cell division proteins in other bacteria, especially in B2H assays (Claessen et al., 2008; Jorge et al., 2011; Steele et al., 2011; Pompeo et al., 2015). To identify complexes that contain EzrA, we performed co-IP experiments on extracts of cross-linked *Spn* cells, as described in section “Materials and Methods.” All strains used in these experiments expressed untagged EzrA (negative control) or epitope-tagged EzrA-FLAG^3^ (bait) (Table 2) or other FLAG-tagged bait proteins (Table 3) in combination with prey proteins that were epitope tagged with -HA or -Myc (Figure 9; Supplementary Figures 22–24).

**TABLE 2:** Co-immunoprecipitation of complexes containing EzrA-FLAG^3^ from cross-linked *Spn* cells*^a^*.

Prey	Mean ratio^b^	Detected prey in complex?^c^	Strains used^d^
**Z-ring regulators**			
FtsZ	22.5 ± 10.9	Yes	IU6933/IU11602
FtsA	25.4 ± 9.5	Yes	IU6933/IU11602
ZapA-HA	1.2 ± 0.02	No	IU10267/IU11322
**Penicillin-binding proteins (PBPs)**			
aPBP2a-HA^4^	1.04 ± 0.12	No	IU7797/IU11610
aPBP2b-HA	1.1 ± 0.04	No	IU6933/IU11602
**Cell-cycle regulation**			
MreC	1.0 ± 0.1	No	IU7797/IU11610

*^a^Co-IP experiments were performed as described in section “Materials and Methods.” Prey proteins were detected with anti-HA, anti-FtsZ, Anti-FtsA, or anti-MreC.*

*^b^The mean ratio is determined by dividing Western blot ROI signals of epitope tagged prey in strain expressing ezrA^+^ by the ROI signal of epitope tagged prey in strain expressing ezrA-FLAG^3^ ([ROI prey-HA]_ezrA_^+^/[ROI prey-HA]_ezrA–FLAG_^3^). ROI values are determined as described previously in Materials and Methods. ±, SEM from two independent biological replicates.*

*^c^A positive interaction was determined based on the mean ratio value greater than two.*

*^d^Complete genotypes are listed in Supplementary Table 1. Strain numbers are listed in the following order; the strain listed first expresses the epitope-tagged prey and untagged EzrA (i.e., IU10302; ftsZ-Myc ezrA^+^), while the second strain listed contains the epitope tagged prey and EzrA-FLAG^3^ as the bait (i.e., IU11340; ftsZ-Myc ezrA-FLAG^3^).*

**TABLE 3:** Co-immunoprecipitation of FLAG-tagged divisome proteins from cross-linked *Spn* cells^a^.

Bait Used	Prey	Mean ratio^b^	Detected prey in complex?^c^	Strains used^d^
FLAG-FtsA	FtsZ-Myc	18.1 ± 10.8	Yes	IU9713/IU11476
	EzrA-HA	223 ± 49	Yes	
DivIVA-FLAG^3^	FtsZ-Myc	1.5 ± 0.5	Marginal	IU9713/IU11414
	EzrA-HA	1.8 ± 0.5	Marginal	
MapZ-FLAG^3^	FtsZ-Myc	1.6 ± 0.5	Marginal	IU9713/IU11430
	EzrA-HA	6.4 ± 0.5	Yes	
FtsK-FLAG^2^	FtsZ-Myc	1.5 ± 0.5	Marginal	IU9713/IU11664
	EzrA-HA	4.3 ± 0.7	Yes	
ZapA-FLAG	EzrA-HA	8.2 ± 1.5	Yes	IU11939/IU11840
	FtsZ	14.2 ± 3.4	Yes	
	FtsA	2.2 ± 0.1	Yes	
aPBP1a-FLAG^3^	EzrA-HA	3.6 ± 1.9	Yes	IU6810/IU12069
	FtsZ	1.3 ± 0.2	No	
	FtsA	1.2 ± 0.2	No	
	MreC	4.6 ± 2.5	Yes	
SepF-FLAG	EzrA-HA	15.9 ± 6.2	Yes	IU6810/IU12076
	FtsZ	1.1 ± 0.2	No	
	FtsA	13.3 ± 2	Yes	
StkP-FLAG^2^	EzrA-HA	13.9 ± 2.3	Yes	IU6810/IU12077
	FtsZ	1.2 ± 0.1	No	
	FtsA	1.7 ± 0.3	Marginal	
bPBP2x-FLAG^3^	EzrA-HA	1.8 ± 0.6	Marginal	IU6810/IU11880
	FtsZ	1.1 ± 0.0	No	
	FtsA	1.7 ± 0.2	Marginal	
FtsZ-FLAG	ZapA-HA	5.9 ± 3.4	Yes	IU10267/IU11322
	FtsA	5.7 ± 1.0	Yes	
	FtsZ	173 ± 79	Yes	

*^a^Co-IP experiments were performed as described in section “Materials and Methods.”*

*^b^The mean ratio is determined by dividing Western blot ROI signals of epitope tagged prey in strain expressing bait^+^ by the ROI signal of epitope tagged prey in strain expressing bait-FLAG^3^ ([ROI prey-HA]_bait_^+^/[ROI prey-HA]_bait–FLAG_^3^). ROI values are determined as described in Materials and Methods. ±, SEM from two independent biological replicates.*

*^c^A positive association was determined based on the mean ratio value greater than 2.0. A mean value between 1.5 and 2.0 was labeled “marginally detected” association because prey proteins were indeed detected in the bait complex, although not “high” relative to prey protein in non-bait complex.*

*^d^Complete genotypes are listed in Supplementary Table 1. Strain numbers are listed in the following order: the strain listed first expresses the epitope-tagged prey and untagged/no bait FtsA (i.e., IU9713; ftsZ-Myc ezrA-HA ftsA^+^), while the second strain listed contains the epitope tagged prey and a FLAG tagged bait protein (i.e., IU11476 ftsZ-Myc ezrA-HA FLAG-ftsA). All strains used here showed wild-type cell morphology and growth characteristics, with the exception of IU11430 that showed some slight cell size variability with rounder cells.*

We tested whether EzrA(*Spn*) can be detected in complexes at some stage of cell division with FtsZ-ring regulators, including FtsA, SepF, and ZapA, as was determined previously by co-IP for EzrA in *Bsu* (Ishikawa et al., 2006). We also tested if EzrA(*Spn*) is in a complex with MapZ, which guides formation of equatorial Z-rings (Fleurie et al., 2014a; Holeckova et al., 2015; Perez et al., 2019). Consistent with a role for EzrA in regulating Z-ring dynamics, EzrA(*Spn*) was detected in complexes with FtsZ and the other inferred Z-ring regulators, FtsA, SepF, ZapA, and MapZ (Figure 9A and Tables 2, 3). Strong co-IP signals were detected for at least one bait:prey combination with EzrA and each of these proteins. For EzrA and ZapA, complex formation was detected for ZapA(bait):EzrA(prey) (Table 3), but not for EzrA(bait):ZapA(prey) (Table 2), perhaps indicating a detection limit of our co-IP assay. Interactions between EzrA and FtsZ, FtsA, SepF, and MapZ were corroborated by B2H assays (Supplementary Figure 21), indicating that EzrA directly interacts with these proteins at some stage of the cell cycle (Figure 9B). In contrast, we did not detect direct interactions in B2H assays between EzrA(*Spn*) and ZapA or its partner ZapJ, which is discussed below (Supplementary Figure 21). This negative result could reflect the inability of ZapA(*Spn*) and ZapJ to interact in the absence of each other in *Eco* or cross-binding of ZapA(*Spn*) to ZapAB(*Eco*) or FtsZ(*Eco*) in B2H assays.

We also probed for EzrA(*Spn*) interactions with PBPs and cell-cycle regulators. In *Bsu* and *Sau*, EzrA interacts with PBPs based on B2H assays (Claessen et al., 2008; Jorge et al., 2011; Steele et al., 2011). In *Spn*, we detected an unambiguous co-IP signal for complexes containing EzrA and aPBP1a (Supplementary Figures 23A,B; Table 3). In contrast, we detected a marginal co-IP signal for complexes containing EzrA and bPBP2x (Supplementary Figure 22C; Table 3) and no detectable complex between EzrA and aPBP2a or bPBP2b (Supplementary Figure 22C; Table 2). Consistent with the co-IP data, B2H assays detected interactions between EzrA and aPBP1a or bPBP2x and a lack of detection with bPBP2b, although an interaction with aPBP2a was detected using B2H (Supplementary Figure 21). We conclude that complexes containing EzrA(*Spn*) also contain certain PBPs (Figure 9B).

We further tested whether EzrA can be detected in complexes with the cell cycle and PG-synthesis regulators DivIVA (morphogenetic determinant Fadda et al., 2007; Fleurie et al., 2014b), StkP (serine/threonine kinase Novakova et al., 2010; Beilharz et al., 2012; Fleurie et al., 2014b), GpsB (regulator of PBP activity Cleverley et al., 2016, 2019; Rued et al., 2017), and FtsK (chromosome partitioning and dimer resolution Le Bourgeois et al., 2007; Burton and Dubnau, 2010; Massidda et al., 2013; Pinho et al., 2013). Previous B2H and surface plasmon resonance assays suggested that EzrA(*Spn*) interacts with GpsB, StkP, and DivIVA (Fleurie et al., 2014b). Previously, we reported complexes containing EzrA and GpsB in *Spn* cells (Rued et al., 2017). We detected strong co-IP signals for complexes in *Spn* cells containing EzrA and StkP or FtsK (Figure 9A and Table 3). In contrast, the signal for DivIVA(prey):EzrA(bait) complexes was marginal (Figure 9A and Table 3), as was the signal for DivIVA(bait):FtsZ(prey) complexes (Supplementary Figure 24A; Table 3), possibly indicating a detection limit of the co-IP assay. Interactions between EzrA and GpsB, StkP, or FtsK were corroborated by B2H assays (Supplementary Figure 21). We also tested for complex the formation between EzrA and MreC (PG elongasome/pPG synthesis regulator) by co-IP and B2H assays. We were unable to detect a complex containing EzrA and MreC in *Spn* by co-IP (Table 2), but we did detect an interaction by B2H assays (Supplementary Figure 21), possibly indicating a transient interaction in cells.

Finally, we used the B2H assay to test for interactions between EzrA(*Spn*) and several proteins not tested in co-IP assays. These assays indicate possible direct interactions between EzrA and MacP (positive regulator of PBP2a) (Fenton et al., 2018), RodA (SEDS GTase in pPG synthesis) (Meeske et al., 2016), MreD and RodZ (PG elongasome/pPG regulators) (Massidda et al., 2013; Briggs et al., 2021), MpgA (formerly MltG; glycosidase in pPG synthesis) (Taguchi et al., 2021), and FtsQ/L (divisome assembly proteins) (Noirclerc-Savoye et al., 2005; Briggs et al., 2021). Taken together, these results show that EzrA(*Spn*) forms complexes and interacts with many key proteins that mediate Z-ring regulation, cell division, and PG synthesis (Figure 9B and Supplementary Figure 21), consistent with its extended spectrin-like repeated structure (Supplementary Figure 1D) and the diverse interactions reported for EzrA in other bacteria. These multiple EzrA interactions support a model in which EzrA is not only required as a positive regulator of Z-ring assembly in *Spn* (above; Figure 6), but functions as a regulator that helps link FtsZ/FtsA filaments/bundles to PG synthesis and cell-cycle checkpoints.

### ZapA Is a Late-Arriving Protein at the Equators of Daughter Cells in Contrast to EzrA

EzrA(*Spn*) is in complexes with several proteins that regulate FtsZ-ring formation, including FtsA, SepF, and ZapA (Figure 9A and Tables 2, 3). FtsA and SepF have been characterized previously in *Spn* (Mura et al., 2016; Perez et al., 2019). FtsA is essential and always co-localizes with FtsZ in *Spn*, whereas Δ*sepF* deletion mutants form elongated, widened cells lacking septal constrictions, suggesting that SepF polymers mediate FtsZ-ring closure during septation, possibly by acting as a curved clamp at the leading edge of the closing septum (Wenzel et al., 2021). Despite these phenotypes, Δ*sepF* mutants grow, similarly, to WT in BHI broth, and the cell morphology defects of Δ*sepF* mutants are largely reversed by FtsA overexpression (Mura et al., 2016). In contrast to SepF, nothing has been reported about ZapA function in *Spn* or other ovococcal bacteria.

**FIGURE 10:**
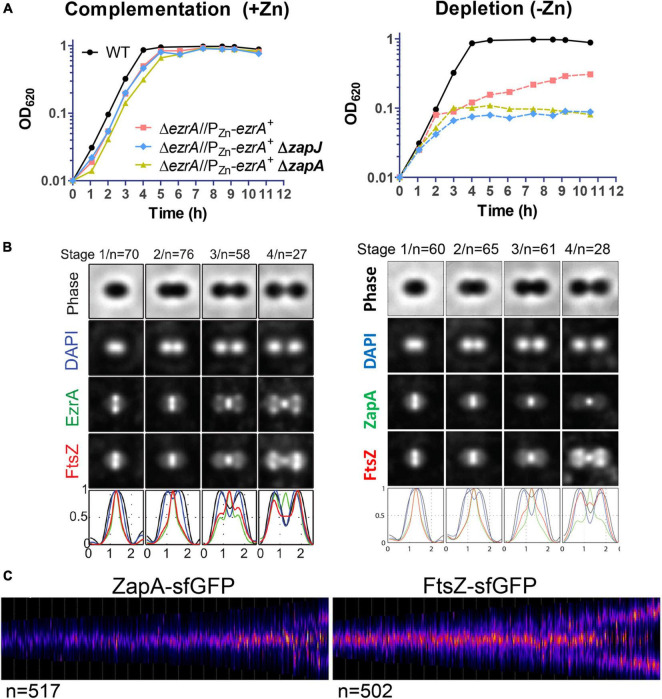
Localization of ZapA relative to FtsZ and combined defects of Δ*zapA* or Δ*zapJ* with EzrA depletion in *Spn*. **(A)** Graph demonstrating the growth curve upon EzrA complementation (left graph) or EzrA depletion (right graph) in otherwise WT, Δ*zapA*, or Δ*zapJ* mutant backgrounds. Wild-type EzrA was induced by supplementation of 0.5 mM ZnCl_2_ and 0.05 mM MnSO_4_ or depleted by growth in the absence of supplemented Zn/Mn. Strains used were IU1945, IU8799, IU10839, and IU15029. **(B)** Averaged images with fluorescence intensity traces showing FtsZ and EzrA localization from IFM performed on IU8681 (left) or IU10752 (right). Cells were binned into division stages 1-4, and images from the indicated number of cells (*n*) from at least two independent biological replicates were averaged using the IMA-GUI program as described in section “Materials and Methods” and previously (Tsui et al., 2014). (i) Row 1, cell shapes determined from phase-contrast images; row 2, nucleoid locations from DAPI labeling; row 3, EzrA (left panel) or ZapA (right panel) locations from IFM; row 4, FtsZ locations from IFM; row 5, normalized mean fluorescence intensity distributions along the horizontal cell axis for each channel (black, phase image; blue, DNA; green, EzrA or ZapA; red, FtsZ). Cells from at least two independent biological replicates were collected at OD_620_ ≈0.1–0.2 and prepared for each set of cells as described in section “Materials and Methods.” **(C)** Demographs of ZapA-sfGFP localization from strain IU10065 compared to FtsZ-sfGFP localization from strain IU9985 obtained using snapshot 2D-EFm of live cells. The indicated number of cells (*n*) from at least two independent biological replicates were analyzed using MicrobeJ and as described in section “Materials and Methods” and previously (Perez et al., 2019).

Co-immunoprecipitation experiments detected ZapA in complex with FtsZ, FtsA, and EzrA in *Spn* cells (Supplementary Figure 25; Table 3), so we compared the spatiotemporal location of ZapA with that of FtsZ. Δ*zapA* mutants did not show any overt growth or cell morphology defects compared to WT in BHI broth or C+Y media (Supplementary Figure 27B; data not shown). We constructed a strain expressing FtsZ-Myc and ZapA-L-FLAG from their chromosomal loci (strain IU10752) and compared their localization to FtsZ-Myc and EzrA-L-FLAG^3^ (IU8681) by 2D EFm (Figure 10B and Supplementary Figure 26A). Unlike EzrA, which co-localizes with FtsZ and moves to the equatorial rings of daughter cells in stages 3 and 4 of the division cycle, ZapA remains at the midcell septal ring until late in cell division (Figure 10B). Moreover, quantitative comparisons of ring widths of C-terminal-tagged EzrA- EzrA-L-FLAG^3^ or ZapA-L-FLAG with FtsZ-Myc indicated that EzrA-rings are slightly larger than FtsZ-rings (Figure 10B and Supplementary Figure 26A), as discussed above (Supplementary Figure 2). In contrast, ZapA-rings have a considerably smaller width than FtsZ widths (Figure 10B and Supplementary Figure 26A), indicative of separate sublocations of EzrA and ZapA in FtsZ-rings.

Late arrival of ZapA at equators compared to FtsZ was confirmed by 3D-SIM of the FtsZ-Myc and ZapA-L-FLAG epitope-tagged strains (Supplementary Figure 26B) and separately by demograph analysis of 2D-EFm images of live cells expressing ZapA-sfGFP (strain IU10065) or FtsZ-sfGFP (strain IU9985) (Figure 10C). These results indicate that ZapA is not present in the nascent MapZ/FtsZ/FtsA/EzrA-rings that move out from the septum to the equators during *Spn* division (Perez et al., 2019) or in early equatorial rings. Consistent with this conclusion, the velocity of FtsZ treadmilling in nascent and early equatorial rings was the same in a Δ*zapA* mutant as the WT *zapA*^+^ strain (Supplementary Figure 27A). Similar to ZapA, 2D-EFm and 3D-SIM showed that SepF remains at septa throughout most of the division cycle and arrives late at the equators of daughter cells (Supplementary Figures 28A,C; Mura et al., 2016). However, unlike ZapA, the width of the SepF ring at the midcell is very similar to that of FtsZ (Supplementary Figure 28B). Together, these results are consistent with the idea that the pneumococcal FtsZ regulators EzrA, FtsA, SepF, and ZapA form a spatially ordered network, analogous to the one proposed for *Eco* (Buss et al., 2015).

### ZapA Is Required for the Slow-Growth Phenotype of EzrA-Depleted *Spn* Cells

As shown above (Figures 3, 4, 6), EzrA acts as a positive regulator of FtsZ-ring formation in *Spn*. We wanted to examine whether ZapA and SepF contribute to this positive regulation. Δ*zapA* mutants do not exhibit growth or cell morphology defects under the culture conditions tested (Supplementary Figure 27B), so we determined the effects on growth when EzrA is depleted in a Δ*zapA* or Δ*sepF* mutant. As noted above, depletion of EzrA results in bacteriostatic spheroid cells for long periods of time (Figure 2; Supplementary Table 3). Lack of ZapA (Figure 10A) or SepF (Supplementary Figure 28D) prevents prolonged growth upon EzrA depletion, whereas lack of MapZ does not (Supplementary Figure 28D). Depletion of EzrA in the Δ*zapA* or in the Δ*sepF* mutant gave mostly viable, irregularly shaped spheroid cells, typical of EzrA depletion in the WT strain (Figure 2B and Supplementary Figure 29). However, Δ*sepF* cells during depletion of EzrA appear larger than Δ*zapA* cells under EzrA depletion (Supplementary Figure 29), consistent with the increased length and width of Δ*sepF* mutants (Mura et al., 2016). By comparison, Δ*zapA* Δ*sepF* mutants grew, similarly, to Δ*sepF* single mutants, forming elongated and widened cells (data not shown; Mura et al., 2016). Finally, lack of ZapA did not alter the growth stoppage and lysis when FtsZ was depleted (Supplementary Figure 27B), but Δ*zapA* Δ*mapZ* mutants grew slightly slower than a Δ*mapZ* mutant (Supplementary Figure 27C). Together, these results are consistent with ZapA and SepF, playing accessory roles to EzrA in positively regulating FtsZ-ring formation, separate from MapZ, in *Spn*.

### ZapA Forms a Complex With ZapJ (Spd_1350), and Δ*zapJ* Phenocopies Δ*zapA*

In *Eco*, ZapA interacts with a partner protein, called ZapB (Ebersbach et al., 2008; Galli and Gerdes, 2012). *Spn* does not encode an obvious ZapB homolog. NCBI blast search of the gene (*spd_0370*) immediately downstream from *zapA* in the *Spn* D39 genome identified a colicin V superfamily protein as a homologue, but not *zapB*(*Eco*). *spd_0370* was annotated as *zapB* (Slager et al., 2018) in D39V, and as a colicin V superfamily protein (Lanie et al., 2007) in D39W, and is a homologue of *yshB*(*Bsu*), a gene downstream of *zapA*(*Bsu*) (BioCyc). To test experimentally whether Spd_0370 might be involved in Z-ring dynamics in *Spn*, we fused the N- and C-termini of Spd_0370 to GFP and to the FLAG epitope tag and determined its localization in *Spn* cells. 2D-EFm showed that Spd_0370 localized to the membrane but did not localize to the midcell divisome region, despite confirmed expression by Western blotting (data not shown). Given its lack of homology to *Eco* ZapB and its localization, we conclude that *spd_0370* does not encode an FtsZ-ring regulator, despite its co-transcription with *zapA* (Slager et al., 2018).

**FIGURE 11:**
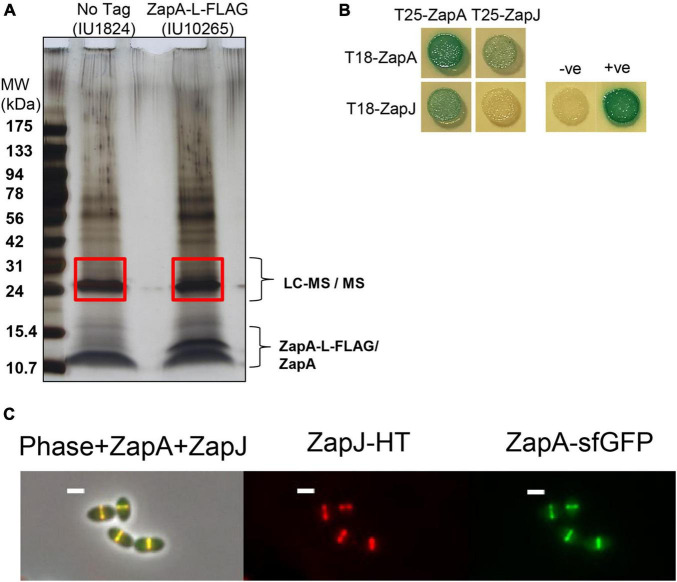
ZapA can co-IP ZapJ (Spd_1350) and these proteins co-localize at midcell rings. Cells were cultured in BHI broth and harvested at OD_620_≈0.1-0.25 for experiments. **(A)** Silver-stained SDS-PAGE gel from Co-IP experiments performed using WT (IU1824, lane 1 non-flagged control) and ZapA-FLAG (IU10265, lane 2) as bait. Red boxes indicate excised bands that were processed for LC-MS/MS (see section “Materials and Methods”). PAGE ruler pre-stained ladder and calibrated MW shown on the left. **(B)** ZapA and ZapJ from *Spn* interact directly, and ZapA self-interacts by B2H assays. T25 or T18 fusions are expressed from low-or high-copy plasmids, respectively. Plasmid pairs pKT25/pUT18C and pKT25-*zip*/pUT18C-*zip* were used as negative (–ve) and positive (+ve) controls. B2H assays were performed as described in *Materials and Methods*. Agar plates were photographed after 40 h at 30°C. B2H assays were performed at least twice with similar results. **(C)** Co-localization of ZapJ-HT and ZapA-sfGFP in the same cells of strain IU15116. Cells were labeled with 500 nM HT-TMR ligand and imaged using conventional microscopy as described previously (Perez et al., 2019). Representative images are shown.

To identify a partner of ZapA(*Spn*), we turned to an unbiased co-IP/MS approach. We formaldehyde-cross-linked *Spn* cells expressing ZapA-FLAG, which was then enriched on magnetic beads, and potential interactors were resolved by SDS-PAGE followed by silver staining as described in section “Materials and Methods” (Figure 11A). A single faint band with a molecular mass of about 24 kDa was present in the extract from cells expressing ZapA-FLAG, but absent from control cells lacking any FLAG-tagged proteins. The region between ≈21 and 31 kDa was excised from both lanes and subjected to peptide analysis by mass spectroscopy. Comparison of peptides from the ZapA-FLAG sample with the untagged ZapA^+^ control revealed that ZapA-FLAG pulled down the protein Spd_1350, which we renamed ZapJ (calculated molecular mass = 23,844 kDa and pI = 9.11). *zapJ* appears to be in a single-gene operon (Slager et al., 2018) and is located upstream of *murC* (Supplementary Figure 30A) that encodes UPD-N-acetylmuramate-alanine ligase, which catalyzes the third reaction in Lipid II precursor synthesis (Supplementary Figure 30A). ZapJ was putatively annotated as a cystathionine γ-synthase (Slager et al., 2018), which is inconsistent with the genetic relationships of Δ*zapJ* mutants that we observed below. B2H assays confirmed a direct interaction between ZapA and ZapJ and a self-interaction of ZapA (Figure 11B).

For every phenotype examined, ZapJ completely phenocopied ZapA. Similar to Δ*zapA* mutants, Δ*zapJ* mutants grew like WT and did not show cell morphology defects under the culture conditions tested (Figure 10A; data not shown). ZapJ fused at its C-terminus to Halotag (HT) or sfGFP localized to the midcell (Figure 11C and Supplementary Figure 30B), and ZapJ co-localized with ZapA in cells at different division stages (Figure 11C). Like ZapA, demograph analysis showed that ZapJ is a late-arriving protein to the equators of daughter cells (Figure 10C and Supplementary Figure 30C). EzrA depletion in a Δ*zapA* or Δ*zapJ* mutant rapidly prevented continued growth (Figure 10A), and the impaired growth of Δ*zapA* or Δ*zapJ* in Δ*mapZ* mutant backgrounds was indistinguishable (Supplementary Figure 27C). Together, these results support the hypothesis that ZapA and ZapJ interact and act as accessory positive regulators of FtsZ-ring formation in *Spn.* Finally, ZapJ is confined to *Streptococci*, which appear to lack a ZapB homolog, and *zapJ* is genetically closely linked to *murC* across *Streptococcus* species (Supplementary Figure 31).

## Discussion

**FIGURE 12:**
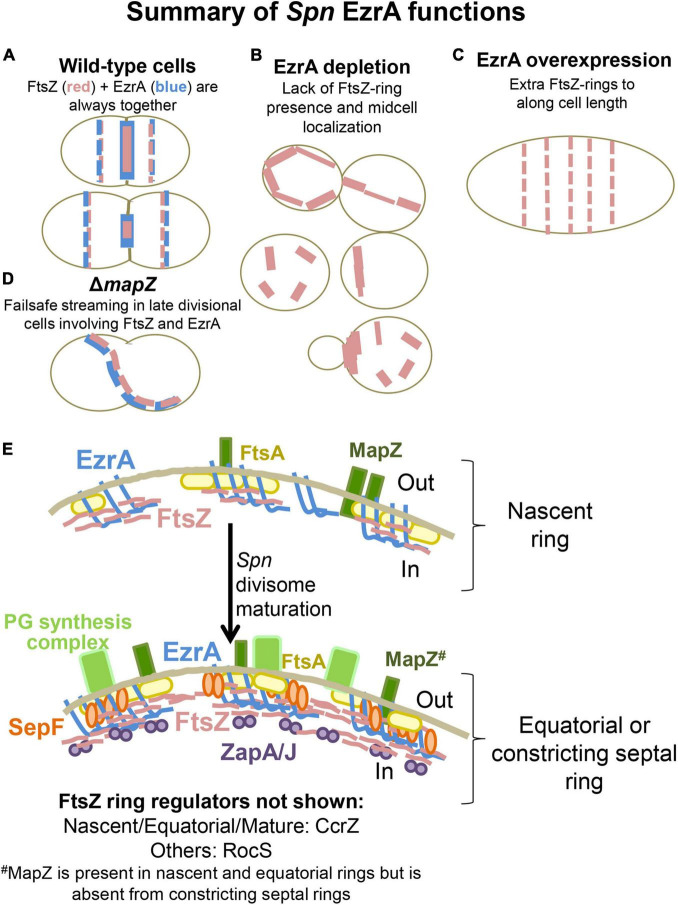
Schematic drawings indicating EzrA as a positive Z-ring regulator in *Spn*, whereas EzrA, SepF, ZapA, and ZapJ form a multi-layered network of proteins that synergistically regulate the FtsZ-ring and cell division in *Spn* during divisome maturation. **(A–D)** Phenotypes of cell shape and FtsZ localizations observed in **(A)** WT, **(B)** EzrA depletion **(C)** EzrA overexpression and **(D)** Δ*mapZ.* **(E)** Summary diagram depicting the spatial localization patterns of FtsZ, EzrA, ZapA/J, SepF, and PG synthesis complexes during divisome maturation in *Spn.* EzrA/FtsZ/FtsA and MapZ form early arriving Z-ring regulators while ZapA/J and SepF are late arriving Z-ring regulators. RocS arrives early but is not divisome associated while CcrZ connects early Z-ring formation with DNA replication. EzrA and FtsA are membrane anchors for FtsZ filaments. SepF localizes with FtsZ, while ZapA, and by extension ZapJ, are on the inside of the Z-ring.

In this paper, we show that EzrA and FtsZ are both essential for the growth, division, ovoid shape, and normal size of pneumococcal cells (Figures 2, 7). *Spn* EzrA and FtsZ always co-localize during the cell cycle, and their localization is interdependent (summarized in Figure 12). The importance of EzrA to the midcell presence and placement of the divisome was corroborated by the complementary approaches of FtsZ localization by IFM (Figure 3) and FtsZ-GFP in live cells by EFm (Figure 4 and Supplementary Figure 11) and localization of regions of PG synthesis by FDAA pulse-chase labeling (Figure 5, and Supplementary Figures 12, 13). Moreover, EzrA depletion resulted in cells with non-medial placement of FtsZ-rings, including minicells lacking DNA (Figures 3B, 12B, Supplementary Figures 10, 11C). Of particular note, overexpression of EzrA led to the formation of extra Z-rings (Figures 6, 12C and Supplementary Figure 14). Finally, we show that a multicomponent network of Z-ring regulators, including EzrA (essential), SepF (non-essential), ZapA (non-essential), and newly identified ZapJ (non-essential), synergistically support cell division in *Spn* (Figures 10, 12E and Supplementary Figure 28). Altogether, this work is consistent with positive roles for EzrA, SepF, ZapA, and ZapJ in promoting Z-ring assembly in *Spn*.

Biochemical and physiological evidence implicates that EzrA is a negative regulator of FtsZ-ring formation in *Bsu* and possibly other bacteria (Levin et al., 1999; Haeusser et al., 2004; Singh et al., 2007; Cleverley et al., 2014). In contrast, the data presented here show that EzrA positively regulates Z-ring formation in *Spn* in the midcell and elsewhere. Depletion of EzrA(*Spn*) leads to cells lacking Z-rings or containing misplaced Z-rings (Figures 3, 4, and Supplementary Figure 11), in contrast to the formation of additional Z-rings in *Bsu* Δ*ezrA* mutants. Consistent with positive regulation, overexpression of EzrA leads to *Spn* cells with multiple extra Z-rings (Figure 6 and Supplementary Figure 14). Other positive regulators of Z-ring formation, including SepF, ZapA, and newly discovered ZapJ, are required for the continued slow growth of cells upon depletion of EzrA(*Spn*) (Figure 10A and Supplementary Figure 28D). Previous work demonstrated that a nascent division ring containing EzrA moves away from the septal ring of WT cells with MapZ, FtsZ, and FtsA toward the equators of daughter cells, presumably driven by pPG synthesis (Perez et al., 2019). By contrast, both SepF and ZapA remain at the septum throughout most of the division cycle and only arrive late at equators (Figures 10B,C and Supplementary Figure 28A; Mura et al., 2016). These results are consistent with EzrA(*Spn*) acting as a positive regulator that corrals FtsZ/FtsA filaments/bundles into new and nascent Z-rings.

Depletion of EzrA(*Spn*) is bacteriostatic for a surprising long period of time (Figure 2A and Supplementary Figure 29), whereas depletion of FtsZ(*Spn*) is catastrophic and leads to drastic decrease in cell viability and the formation of large spherical cells that are rapidly autolysed by the stress-induced LytA PG amidase (Figure 7A and Supplementary Figure 15A; Mellroth et al., 2012; Flores-Kim et al., 2019). FtsZ depletion in *Spn* severely disorganizes the localization of EzrA, FtsA, and PG synthesis from their normal positions in septal and equatorial rings (Figure 8 and Supplementary Figures 16–20). While we have previously shown that early-onset depletion of FtsA leads to more heterogeneous cell shapes (Mura et al., 2016) than FtsZ depletion shown here, later depletion timepoints of FtsA also show enlarged and completely sphere-shaped cells caused by diffuse and disorganized PG synthesis. This terminal FtsA depletion phenotype resembles FtsZ depletion reported here. This disorganization is consistent with the model that the FtsZ/FtsA-ring initially organizes both sPG and pPG synthesis at midcells of pre-divisional *Spn* cells and continues to organize sPG synthesis during septal constriction as division progresses (Briggs et al., 2021; Perez et al., 2021). It also underscores the absence of a separate mechanism for the organization of pPG synthesis, analogous to that mediated in rod-shaped bacteria by MreB, which maintains continued lateral synthesis when FtsZ is depleted, resulting in long, filamentous cells (Beall and Lutkenhaus, 1991; Dai and Lutkenhaus, 1991). Similar disorganization of PG synthesis caused by FtsZ depletion occurs in *Sau* (Pinho and Errington, 2003) and likely occurs in other coccal and ovococcal species that lack MreB.

In Δ*mapZ* mutants, EzrA still co-localizes and streams with FtsZ by a failsafe mechanism to form new Z-rings near the middles of daughter *Spn* cells (Supplementary Figures 4, 12D; Perez et al., 2019). Consequently, Δ*mapZ* mutants show relatively minor defects in Z-ring presence and placement, compared to the severe effects caused EzrA depletion (Figure 3 and Supplementary Figures 2A,B). In this work, we further demonstrated co-localization of EzrA and FtsZ treadmilling dynamics in nascent and equatorial rings of WT cells (Supplementary Figure 5) and during streaming in Δ*mapZ* mutants (Supplementary Figure 3). The TM domain and the conserved C-terminal QNR motif of EzrA(*Spn*) are required for localization and function (Supplementary Figure 9), similar to what was reported previously for EzrA(*Bsu*) (Cleverley et al., 2014; Land et al., 2014). Other mutations near the C-terminal QNR motif of EzrA(*Spn*) result in a temperature-sensitive phenotype (Perez et al., 2021). Together, these results support a critical role for EzrA(*Spn*) in anchoring FtsZ filaments/bundles to membranes, whereas MapZ guides FtsZ/EzrA/FtsA bundles filaments/bundles from the nascent ring to the equators of daughter cells.

Ensemble labeling shows that EzrA(*Spn*) moves dynamically with FtsZ and FtsA in filaments/bundles at a velocity of ≈34 nm/s in nascent and equatorial rings of dividing *Spn* cells (Supplementary Figure 5; Perez et al., 2019). Likewise, EzrA-GFP expressed from the native *ezrA* promoter in *Sau* moves with a similar velocity to FtsZ filaments/bundles (Monteiro et al., 2018). At the same time, single molecules of FtsZ in filaments appear stationary, because of the treadmilling mechanism (Bisson-Filho et al., 2017; Yang et al., 2017; Perez et al., 2019). It was recently reported that single molecules of EzrA(*Bsu*) and other FtsZ-binding proteins appear to be immobile at division sites in treadmilling FtsZ filaments/bundles (Squyres et al., 2021). Similarly, some single molecules of EzrA(*Spn*) appear to be immobile at the midcell of *Spn* cells (data not shown). In addition, we observed a population of EzrA that is not strongly associated with FtsZ or FtsA in equatorial and nascent rings (Supplementary Figure 5). These combined results suggest that there are two populations of EzrA at *Spn* division sites: (i) diffusing, FtsZ-unassociated EzrA molecules are not bound to treadmilling FtsZ/FtsA filaments/bundles, but are confined to the division ring plane and (ii) immobile, FtsZ-associated EzrA molecules are transiently bound to and released from treadmilling FtsZ/FtsA filaments/bundles, resulting in collective migration (Baranova et al., 2020). Finally, the processive ensemble motion of FtsZ/EzrA/FtsA filaments/bundles in *Spn* is distinct from the processive single-molecule motions of septal cell-wall synthase PBP2x:FtsW(*Spn*) complexes. Processive septal cell-wall synthase complexes are driven by PG synthesis independent of FtsZ treadmilling (Perez et al., 2019) or from the treadmilling-linked Brownian-ratchet mechanism that moves non-synthesizing bPBP(FtsI) in the septum of *Eco* cells (McCausland et al., 2021; Yang et al., 2021).

We show that EzrA(*Spn*) is in complexes with numerous proteins involved in FtsZ-ring placement (MapZ) and stabilization (FtsZ, FtsA, ZapA, and SepF), cell division and chromosomal segregation (FtsK and possibly DivIVA), PG synthesis (aPBP1a and possibly bPBP2x), and PG regulation (GpsB and StkP) (Figure 9B) at some stage in cell division. EzrA(*Spn*) has the capacity to bind directly to all of these proteins, with the possible exception of ZapA, based on B2H assays (Supplementary Figure 21). Moreover, a new paper reports that EzrA(*Spn*) interacts directly with the CcrZ cell-cycle regulator that also interacts with FtsZ, FtsA, and ZapA and couples cell division with DNA replication through control of DnaA activity (Gallay et al., 2021). Wide binding versatility of EzrA was previously noted in *Sau*, although where and when these diverse proteins bind to the spatially complex EzrA spectrin-like loop structure (Supplementary Figure 1) remains to be determined. The essentiality, different phenotypes caused by EzrA depletion compared to FtsZ depletion, including FDAA labeling efficiency (Supplementary Figure 12), and the inability to isolate stable suppressor mutations of Δ*ezrA* (see section “RESULTS”) are consistent with EzrA orchestrating Z-ring regulation with cell division and PG synthesis in *Spn*.

EzrA remains associated with FtsZ in all locations throughout the cell cycle of *Spn* cells (Figures 1, 12), including the following: in FtsZ/FtsA/EzrA nascent ring planes that move with MapZ outward to the equators of daughter cells (Supplementary Figures 3, 5; Perez et al., 2019); in constricting septa (Figure 1 and Supplementary Figure 2C) until remaining FtsZ migrates to the equators of daughter cells before sPG synthesis is completed (Supplementary Figure 6); and in streaming FtsZ filaments in Δ*mapZ* mutants (Supplementary Figure 4). FtsA also shows very similar localizations and dynamics as FtsZ and EzrA (Mura et al., 2016; Perez et al., 2019). The other FtsZ-positive regulators in *Spn*, SepF, ZapA, and presumably its partner ZapJ arrive later to the FtsZ-rings that form at the equators of daughter cells (Figure 10, Supplementary Figures 28, 30) (Mura et al., 2016). This pattern contrasts sharply with the early arrival of ZapA with FtsZ and FtsA in rod-shaped bacteria (Aarsman et al., 2005; Gamba et al., 2015). Nevertheless, SepF, ZapA, and ZapJ are required to maintain the slow growth phenotype of EzrA-depleted *Spn* cells (Figure 10A and Supplementary Figure 28D), suggesting accessory roles of these proteins to EzrA in maintaining FtsZ-rings in mature equatorial and septal rings. FtsA was shown to be essential, and prolonged FtsA depletion resulted in cell lysis and severe mislocalization of FtsZ. Thus, FtsA is another essential positive Z-ring regulator while also playing a role in sPG and pPG synthesis (Mura et al., 2016). The association of EzrA and FtsZ in nascent rings that lack other accessory FtsZ-positive regulators may underlie why EzrA functions as a positive regulator in *Spn*, instead of a negative regulator as in *Bsu*.

The discovery of ZapJ as a partner for ZapA (Figures 11A, 12) may account for the lack of ZapB homologues in *Streptococcus* species. Similar to ZapA(*Eco*) and ZapB(*Eco*), ZapA(*Spn*) and ZapJ(*Spn*) localize in the midcell and interact directly with each other (Galli and Gerdes, 2010, 2012). Additionally, similar to Δ*zapA*(*Eco*) and Δ*zapB*(*Eco*) (Buss et al., 2013), Δ*zapA*(*Spn*) phenocopied Δ*zapJ*(*Spn*) in every physiological assay shown in this study, further suggesting that ZapA:ZapJ function together as part of the same complex in *Spn* and potentially other *Streptococci*. While ZapJ is conserved in *Streptococci*, ZapB is absent from Gram-positive bacteria and, instead, restricted to the Gammaproteobacteria class (Adams and Errington, 2009; Huang et al., 2013). Within the resolution limits of IFM methods, EzrA, FtsA, and ZapA form rings with different diameters relative to FtsZ in *Spn* (Supplementary Figures 2C, 26; Mura et al., 2016), consistent with a multilayered network of FtsZ stabilizers (Figure 12E), analogous to the that proposed for *Eco* (Buss et al., 2015; Coltharp et al., 2016). Recently discovered CcrZ, which controls DNA replication and clearly interacts directly with FtsZ in *Spn* cells, couples the timing of DNA replication to Z-ring placement in this multicomponent network (Gallay et al., 2021), replacing *Eco* MatP, which is absent in *Streptococcus* species. Aberrant timing of DNA replication at the midcell FtsZ-ring in mutants lacking CcrZ leads to chromosome mis-segregation and anucleate cells (Gallay et al., 2021), similar to what occurs when the FtsZ-ring structure is disrupted by EzrA depletion in *Spn* (Supplementary Figure 10). Future studies are needed to understand the roles of EzrA, SepF, ZapA:ZapJ, and FtsA in mediating septal closure and chromosome segregation in *Spn.*

Finally, *Streptococcus* species are resistant to compound PC190723, which inhibits FtsZ polymerization in *Sau*, *Bsu*, *Eco*, *Bacillus anthracis*, and other bacteria (Haydon et al., 2008). The discovery of ZapJ and further characterization of FtsZ-ring regulation offer the potential for identifying new vulnerabilities for antibiotic discovery against *Spn* and other *Streptococci*, which are becoming increasingly antibiotic resistant (Centers for Disease Control and Prevention, 2019).

## Data Availability Statement

The original contributions presented in the study are included in the article/Supplementary Material, further inquiries can be directed to the corresponding author/s.

## Author Contributions

AP, H-CT, and MW contributed to the conception and design of this study. AP, JV, H-CT, MD, MB, and OM contributed to the acquisition, analysis, and interpretation of data. AP and MW contributed to the writing and editing of the manuscript with input from the other authors. All authors contributed to the article and approved the submitted version.

## Conflict of Interest

The authors declare that the research was conducted in the absence of any commercial or financial relationships that could be construed as a potential conflict of interest.

## Publisher’s Note

All claims expressed in this article are solely those of the authors and do not necessarily represent those of their affiliated organizations, or those of the publisher, the editors and the reviewers. Any product that may be evaluated in this article, or claim that may be made by its manufacturer, is not guaranteed or endorsed by the publisher.
