# Supplementary material for: FtsZ-Ring Regulation and Cell Division Are Mediated by Essential EzrA and Accessory Proteins ZapA and ZapJ in Streptococcus pneumoniae
Source: Front Microbiol. 2021 Dec 2;12:780864. doi: 10.3389/fmicb.2021.780864 (PMC8687745; doi:10.3389/fmicb.2021.780864)
Supplement: Supplementary file 1 [file Data_Sheet_1.PDF]

Supplementary Material

FtsZ-Ring Regulation and Cell Division Are Mediated by Essential EzrA and Accessory Proteins ZapA and ZapJ in *Streptococcus pneumoniae*

Amilcar J. Perez[a], Jesus Bazan Villicana[a], Ho-Ching T. Tsui[a], Madeline L. Danforth[a], Mattia Benedet[b], Orietta Massidda[b], and Malcolm E. Winkler[a#]

[a]Department of Biology, Indiana University Bloomington, Bloomington, IN USA

[b]Department of Cellular, Computational, and Integrative Biology (CIBIO), University of Trento, Italy

[#]Corresponding author:
Malcolm E. Winkler
Department of Biology
Indiana University Bloomington
1001 E 3rd Street
Bloomington, Indiana USA 47405
Phone: 812-856-1318
E-mail: winklerm@indiana.edu

1. SUPPLEMENTARY TEXT: Complete Materials and Methods

2. SUPPLEMENTARY TABLES: Supplementary Tables 1-7

3. SUPPLEMENTARY REFERENCES

4. SUPPLEMENTARY FIGURES AND LEGENDS: Supplementary Figures 1-31

1. SUPPLEMENTARY TEXT

Complete Material and Methods

EzrA Structure Modeling

The amino acid sequence of EzrA(*Spn*) containing only the cytoplasmic portion (amino acids 30-end) were entered into the phyre^2 server under "normal modeling" (Kelley *et al.,* 2015). The alignment of EzrA(*Spn*) with EzrA(*Bsu*) (pdb accession number is 4UXV, "Cytoplasmic domain of bacterial cell division protein EzrA"). The resulting PDB model was visualized and aligned in PyMOL (The PyMOL Molecular Graphics System; Version 1.7.4.3; Schrödinger, LLC 2; www.pymol.org).

Bacterial Strains and Growth Conditions

Bacterial strains used in this study were derived from strain IU1824; unencapsulated derivative of serotype 2 *S. pneumoniae* strain D39 containing an allele conferring Streptomycin resistance (*rpsL1*) or IU1945 (streptomycin sensitive; *rpsL*$^+$)((Lanie *et al.,* 2007); Supplementary Table 1). Strains containing antibiotic resistance markers were constructed by transforming linear DNA amplicons synthesized by overlapping fusion PCR into competent pneumococcal cells as described previously (Tsui *et al.,* 2010). Primers used for the generation of amplicons are listed in Supplementary Table 2. All constructs were confirmed by DNA sequencing of chromosomal regions corresponding to the amplicon region used for transformation. Bacteria were grown in plates containing trypticase soy agar II (modified; Beckton-Dickson) and 5% (v/v) defribrinated sheep blood (TSAII-BA). Plates were incubated at 37°C in an atmosphere of 5% CO_2. For antibiotic selections, TASII-BA plates contained 250 µg kanamycin ml^{-1}, 150 µg spectinomycin ml^{-1}, 0.3 µg erythromycin ml^{-1}, 250 µg streptomycin ml^{-1}, or 0.25 µg tetracycline ml^{-1}. Strains were cultured statically in Becton-Dickinson brain heart infusion (BHI) broth at 37°C in an atmosphere of 5% CO_2, and growth was monitored by OD_{620} as described before (Land *et al.,* 2013; Tsui *et al.,* 2014). Bacteria were inoculated into BHI broth from frozen cultures or colonies, serially diluted into the same medium, and propagated for 12-16 h. For growth experiments (non-depletion strains), overnight cultures that were still in exponential phase (OD_{620} = 0.1–0.4) were diluted back to $OD_{620} \approx$ 0.001-0.012 to start final cultures, which lacked antibiotics in BHI broth at 37°C in an atmosphere of 5% CO_2. Cells were grown in C+Y pH 6.9-7.1 in an atmosphere of 5% CO_2 only when indicated in specific figure legends (Supplementary Figure 5, 14, and 27A).

Growth Merodiploid strains and Zn-Dependent Depletion

In all experiment that utilize $ZnCl_2$ for Zn-dependent ectopic gene expression (including EzrA and FtsZ depletion and EzrA overexpression strains), indicated amounts of $ZnCl_2$ were used alongside 1/10 $MnSO_4$ to prevent $ZnCl_2$ toxicity in growth media and on TSAII-BA plates (Jacobsen *et al.*, 2011; Tsui *et al.*, 2014; Tsui *et al.*, 2016). Depletion strains requiring $ZnCl_2$ for growth were grown overnight in BHI broth in the presence of 0.5 mM $ZnCl_2$ and 0.05 mM $MnSO_4$ for EzrA depletion strains or 0.3 mM $ZnCl_2$ and 0.03 mM $MnSO_4$ for FtsZ depletion strains. To deplete EzrA or FtsZ, cells grown to $OD_{620} \approx$ 0.1-0.25 in the presence of $ZnCl_2$ and $MnSO_4$, were collected by centrifugation (5 min at 16,000 × *g* at 25°C), and re-suspended to appropriate OD_{620} in BHI with or without $ZnCl_2$ and $MnSO_4$, such that cell density at the indicated collection time point(s) was between $OD_{620} \approx$ 0.075-0.25. The resuspension OD_{620} was 0.036 or 0.012, for 1 h or 2-4 h collection time points, respectively. Cells were collected at appropriate time intervals and cell density for live

cell imaging, fixation for immunofluorescence microscopy (IFM), phase contrast microscopy, FDAA labelling, LIVE/DEAD staining, or western blotting experiments.

Cell Fixation and Adherence to Coverslips for Fluorescence Microscopy (Fm)

Cell fixation and adherence to coverslips was performed as described previously (Tsui *et al.,* 2016). Briefly, after exponentially growing cells (OD_{620}≈0.075-0.25) had been washed in cold (4 ºC) PBS and pelleted, supernatant was removed and pellet was re-suspended in 1mL of 4% paraformaldehyde (EMS; 157-4) and incubated for 15 min at room temperature, followed by 45 min on ice. Fixed cells were centrifuged (5 min at 16,000 × *g* at 4°C), and pellets were washed three times with cold (4°C) PBS at 4°C as described above. After the third wash and centrifugation, cells were resuspended in 0.1-0.3 mL of cold (4°C) GTE buffer (50 mM glucose, 1 mM EDTA, 20 mM Tris-HCl, pH 7.5), and stored in the dark at 4° C for up to 16 h. All samples that were fixed, with the exception of vertically oriented cells, were prepared on coverslips and were treated with methanol as described previously for direct imaging or processing for IFM (Tsui *et al.,* 2016).

Characterization of Antibodies for IFM

Antibodies used and incubations used varied per experiment and have shown to be optimal for antibody labeling (Land *et al.,* 2013; Tsui *et al.,* 2014; Tsui *et al.,* 2016) and are listed in Supplementary Table 5. DNA in nucleoids was stained using mounting media SlowFade gold anti-fade reagent with DAPI (Life Technologies, S36936). Control experiments showed no detectable antibody labelling in cells not expressing tagged proteins (IU1824 or IU1945) with the combinations of antibodies used, with exception of anti-FtsZ (data not shown). Labelling of strains containing single tagged-proteins were tested with the double labelling procedure (that contained both sets of primary and secondary antibodies) and produced signal in the expected fluorescence channel only (data not shown).

Analysis of 2D-Epifluorescence Microscopy (EFm) Images

Localization of FLAG-, Myc-, and HA-tagged proteins by IFM (Tsui *et al*., 2014) or localization of sfGFP, GFP, HT by single frame imaging of live cells was performed for exponentially growing cells as described before (Perez *et al*., 2019).

Following 2D-image acquisition of pneumococcal cells, images were aligned and cells were individually picked and were manually binned into four division stages (pre-, early-, mid-, and late-divisional) using a point-and-click IMA-GUI organized in MATLAB as reported previously (Land *et al.,* 2013; Tsui *et al.,* 2014).

Demographs showing protein fluorescence intensity as a function of cell length were generated by using Microbe J (version 5.11s) as described using previous parameters that allow for inclusion of stage 4 cells in the analysis (Perez *et al.,* 2019). For these experiments only live cells were visualized that had been acquired by single frame imaging.

3D-SIM IFM

Samples were prepared as described previously (Land *et al.,* 2013; Tsui *et al.,* 2014) and 3D-SIM was performed using the OMX 3D-SIM super resolution system located in the Indiana University Bloomington Light Microscopy Center (LMIC). Exposure times and %T settings for DAPI, Alexa-

488, and Alexa 568 images were 10-100 ms and 50%, 50 ms and 1-10%, and 50 ms and 10-50% respectively.

TIRF Microscopy (TIRFm)

TIRFm was performed on cells grown in C+Y pH 6.9-7.1 at 37°C and imaged on C+Y agarose pads as described previously (Perez *et al.,* 2019) with 1 frame aquired per second and with 45 ms exposure times. Strain numbers, relevant strain features, and laser power used (% Transmission or %T) for the indicated excitation laser wavelength are indicated; IU15768 (FtsZ-sfGFP EzrA-HT^{JF549}) 10% T at 488 nm and 50% T at 561 nm; IU15699 (GFP-FtsA EzrA-HT^{JF549}) 100% T at 488 nm and 50% T at 561 nm; IU9985 (FtsZ-sfGFP) 10% T at 488 nm; IU14131 (Δ*zapA* FtsZ-sfGFP) 10% T at 488 nm. HaloTag proteins were labelled to saturation with 500 nM HT-JF549 ligand (Grimm *et al.,* 2015).

3D-SIM of FDAA-Labeled Cells Expressing EzrA-sfGFP

To image vertically oriented cells of strain IU10254 (EzrA-sfGFP) we performed 3D-SIM on samples prepared similarly described in (Perez *et al.,* 2021). Cells from overnight cultures were diluted to OD_{620}≈0.02 in 2 mL of fresh BHI broth. At OD_{620}≈0.22, 575 µL of cultures was centrifuged at 16,100 × *g* for 5 min at room temperature, and pellets were resuspended in 250 µL of BHI broth containing TADA (125 µM final). Cells were incubated at 37°C for 5 min, chilled on dry ice for 20 s, and centrifuged for 2.5 min at 16,100 × *g* at 4°C. Cell pellets were centrifuged and washed twice with 500 µL ice-cold PBS, then centrifuged a third time and resuspended in 500 µL of 4% (v/v) paraformaldehyde and incubated in the dark for 15 min at room temperature, followed by 45 min on ice in the dark. Fixed cells were centrifuged, and pellets were resuspended in 100 µL of ice-cold GTE buffer (50 mM glucose, 20 mM Tris-HCl, pH 7.5, 1 mM EDTA). For imaging, cells were centrifuged for 5 min at 16,100 × *g* at 4°C to remove the GTE buffer and centrifuged once more to remove residual GTE buffer with a P20 pipette. Excess liquid was allowed to evaporate for approximately 1 min before pellets were resuspended in 3.0 µL of Vectashield Hardset Antifade (Vector Laboratories, H-1400) with vortexing 1.2 µL of resuspended cells was pipetted onto a 12 mm/1.5 round coverslip (EMS, 72230-01), and a microscope slide was carefully placed on top. The slide was incubated coverslip side down at room temperature in the dark for 15 min, and then viewed by 3-Dimensional Structured Illumination Microscopy (3D-SIM). Exposure time and %T settings to acquire images were 5 ms and 50% for TADA and sfGFP.

Measurements of Cell Dimensions by Phase-Contrast Microscopy (PCm)

Live cells were used for PCm and cell length and width measurements. Cells were grown in BHI broth in the presence or absence of $ZnCl_2$ and $MnSO_4$ using Nikon NIS-Element AR software as described before (Land *et al.,* 2013). For all strains, either stage one cells or daughters of stage four cells were analyzed. Length was defined as the long-axis of post divisional (daughters of stage 4 cells) or stage one cells, while width was parallel planes of daughter cells. Aspect ratio was determined by dividing length by width. The relative volume of cells was determined by dividing the width^2 x length of an individual cell by median width^2 x length value of wild-type strain (IU1945).

Western Blotting

Cell were obtained from exponentially growing cultures. Total cell lysates were prepared using SEDS (0.1% deoxycholate, 150 mM NaCl, 0.2% SDS, 15 mM EDTA pH 8.0) lysis buffer as

described previously (Beilharz *et al.,* 2012; Cleverley et al., 2019). FLAG-, HA-, and Myc-tagged proteins were detected by western blot using 1:1000 dilution of anti-FLAG rabbit polyclonal antibody (Sigma, F7425), anti-HA rabbit polyclonal antibody (Invitrogen, 71-5500), anti-GFP rabbit (ThermoFisher #A11122), or anti-Myc rabbit polyclonal antibody (Sigma, C3956) as primary antibodies for 1 hr incubations. Native untagged proteins were detected using anti-FtsZ at 1:10000 (Lara *et al.,* 2005), anti-FtsA at 1:10000 (Lara *et al.,* 2005), or anti-MreC at 1:5000 (Land and Winkler, 2011) as primary antibodies for 1 h incubations. Secondary incubations were performed using HRP Donkey anti-rabbit for 1 h at a 1:10000 ratio. Chemiluminescent signal in protein bands was detected and quantified using an IVIS imaging system as described previously (Wayne *et al.,* 2010). Following imaging and data acquisition of immunoblot, India-ink was used to confirm equal amount of cell lysate loading throughout all lanes of the nitrocellulose membrane, briefly, 10 μL of india ink was added to 10 mL of PBST and allowed to incubate with used-nitrocellulose membrane overnight. The membrane was then washed with 6 mL of PBS for 5 min twice to remove excess india ink.

LIVE/DEAD Staining of *ezrA* and Other Mutants

Viability determinations were done using the LIVE/DEAD *Bac*Light bacterial viability kit (Molecular Probes) as described before in (Sham *et al.,* 2013; Wayne *et al.,* 2012) with slight modifications. With this assay, a mixture of SYTO 9 and propidium iodide stains bacteria with intact cell membranes and bacteria with damaged membrane fluorescent green and red respectively. Briefly, strains (Supplementary Table 3 and Supplementary Figure 29) were grown at 37° C overnight and depletion or complementation occurred as described above in "Growth of Zn-dependent Depletion and Merodiploid strains". At appropriate times indicated in respective figure legends, cells were collected by centrifugation at 16,100 x *g* for 2 min at 25°C. Cell pellets were resuspended in 50 μL of BHI broth by gentle pipetting to which 2 μL of a 1:1 (v/v) mixture of Syto-9 and propidium iodide was added, according to the manufacturer's instructions, by gentle pipetting. The staining mixture was incubated in the dark for 5 min at 25°C, and cells were visualized by PCm and EFm as described previously (Wayne *et al.,* 2012).

DAPI Staining for Nucleoid Content

DNA in nucleoids was stained using mounting media SlowFade gold anti-fade reagent with DAPI (Life Technologies, S36936) (Tsui *et al*., 2014; Perez *et al*., 2019). Briefly, cells were grown in BHI under appropriate conditions. At the indicated times, cells were fixed as described in "Cell fixation and cell adherence to coverslips for fluorescence microscopy." Immediately prior to imaging 3 μL of DAPI (Life Technologies, S36936) was added to the adhered cells, and coverslips were sealed onto glass slides and visualized immediately. Cells were then scored based on the presence or absence of DAPI staining to give values in Supplementary Table 4.

FDAA Pulse-Chase Labeling in Depletion Experiments

FDAAs HADA (7-hydroxycoumarin-3-carboxylic acid 3-amino-D-alanine), and TADA (tetramethylrhodamine 3-amino D-alanine) were synthesized as reported previously (Boersma *et al.,* 2015; Perez *et al*., 2021). Cells from exponentially growing cultures were spun and re-suspended to an OD_{620} between 0.01-0.036 in 2 or 3 mL of warmed BHI broth (± Zn) containing 1.0 μL of 500 mM HADA (in dimethyl sulfoxide [DMSO]) to a final concentration of 250 μM. At appropriate time intervals 500 μL of cell were transferred to a 2.0 mL centrifuge tube, which were placed in an ice bath for 1 min to halt labeling and centrifuged for 5 min at 16,000 x *g* at 4°C. Supernatants were

discarded, and pellets were resuspended in 250 µL of warm BHI (-Zn) which contained pre-warmed TADA in DMSO to a final concentration of 500 µM. Cultures were placed back into an incubator and grown at 37°C for indicated time amounts in Supplementary Figure legends 12A, and 19A. Cultures were then placed in an ice bath for 1 min to halt labeling and centrifuged for 2.5 min at 16,000 × *g* at 4°C. Cultures were then centrifuged at 16,000 × *g* for 2.5 min at 4°C, supernatants discarded, and pellets were re-suspended in 250 µL of cold 1X PBS. After the second wash and centrifugation, pellets were re-suspended in 1 mL of 4% paraformaldehyde (EMS; 157-4) for fixation as described above for "Cell Fixation and Cell Adherence to Coverslips for Fluorescence Microscopy."

Co-Immunoprecipitation (Co-IP) Assays

Co-IP experiments involving crosslinking steps were performed as described previously (Rued *et al.,* 2017). Briefly, washed cell pellets of FLAG-tagged or control non FLAG-tagged strains grown in 400 mL BHI broth were crosslinked in 0.1 % paraformaldehyde for 1 h at 37°C, washed, resuspended in cold lysis buffer (50mM Tris-HCl pH 7.4, 150 mM NaCl, 1 mM EDTA, 1% Triton X100 (w/v)) with protease inhibitor (ThermoFisher Scientific), and shaken in a FastPrep homogenizer (MP Biomedical). 4 mg proteins and anti-FLAG magnetic beads (Sigma) were incubated for 2 h at 4°C, washed, and complexes containing FLAG-tagged proteins were eluted with FLAG elution buffer containing 150 ng FLAG3 peptide/µL. Input sample and eluted sample were mixed with equal volumes of 2× Laemmli sample buffer (Bio-Rad) containing 5% (vol/vol) β-mercaptoethanol (Sigma) and heated at 95 °C for 1 h to break the cross-links. 5 µL or 20 µL of input samples or eluted samples were loaded into each lane of 4-15% precast protein gels (Bio-Rad) in Tris-glycine buffer. Transferred membranes were subjected to Western blotting using anti-FLAG (Sigma), anti-HA (ThermoFisher Scientific), anti-Myc (Sigma), anti-FtsZ, anti-FtsA, and anti-MreC as primary antibody as described above.

Co-IP experiments not involving crosslinking was performed in Figure 1C with strains IU10447 and IU5456 as described above with the omission of paraformaldehyde incubation.

Bacterial Two-Hybrid (B2H) Assays

The hybrid plasmids used in the B2H assays are listed in Supplementary Table 6. For cloning, the target genes were amplified by PCR from *S. pneumoniae* D39 chromosomal DNA using the primers listed in Supplementary Table 7. PCR fragments for *sepF*, *mpgA* (formerly *mgltG*(*Spn*), *rodZ*, *rodA*, *ftsW*, *ftsQ*/*divIB*, *ftsL*, *ftsB/divIC*, *macP*, *ftsK*, *zapA*, *zapJ,* and *mreD* were purified, digested with the appropriate restriction enzymes, and cloned into the corresponding sites of the pKT25/pUT18C or pKNT25/pUT18 vectors to generate plasmids encoding the corresponding hybrid proteins fused at their C-terminal ends of the T25/T18 fragments (*sepF*, *mpgA*, *rodZ*, *rodA*, *ftsW*, *ftsQ*/*divIB*, *ftsL*, *ftsB/divIC*, *macP, ftsK, zapA, zapJ*) or at their N-terminal ends the of the T25/T18 fragments (*mreD*), respectively. *E. coli* DH5α or XL1-blue transformants were selected on LB agar plates containing ampicillin (100 µg/ml) or kanamycin (50 µg/mL) and 0.4% glucose to repress leaky expression (Karimova *et al.,* 2005). The correct sequence of each construct was verified by double-strand sequencing using primers also listed in Supplementary Table 7. The hybrid plasmids pKT25-*ftsA*/pUT18C-*ftsA,* pKNT25-*ftsZ*/pUT18-*ftsZ*, pKNT25-*gpsB*/pUT18-*gpsB*, pKNT25-*stkP*/pUT18-*stkP,* pKNT25-*ezrA*/pUT18-*ezrA,* pKT25-*pbp1a*/pUT18C-*pbp1a*, pKT25-*pbp2a*/pUT18C-*pbp2a,* pKT25-*pbp2x*/pUT18C-*pbp2x,* pKT25-*pbp2b*/pUT18C-*pbp2b* and pKT25-*mreC*/pUT18C-*mreC* were previously constructed and reported (Krupka *et al.,* 2012; Rued *et al.,* 2017; Cleverley *et al.,*

2019). The B2H plasmids pKNT25-*locZ* and pUT18-*locZ* were kindly provided by K. Buriánková and P. Branny. Plasmid pairs pKNT25/pUT18 and pKT25-*zip*/pUT18C-*zip* were used as negative and positive controls, respectively. B2H assays were carried out as previously described (Rued *et al.*, 2017; Cleverley *et al.,* 2019). Briefly, each pair of plasmids was co-transformed into the *E. coli cya-* strain BTH101 and co-transformation mixtures were spotted directly onto LB agar plates supplemented with ampicillin (100 µg/ml), kanamycin (50 µg/mL) and X-Gal (60 µg/ml), followed by incubation at 30°C. Plates were inspected and photographed after 24 h and 40 h. All the B2H experiments were performed at least twice.

Mass Spectrometry to Identify ZapJ (Spd_1350)

Co-IP with crosslinking was performed on strains IU1824 and IU10267 as described above with following changes. 50 µL (instead of 20 µL) of the eluted sample was loaded onto an SDS-PAGE gel. Silver staining was performed as described by manufacturer's instructions (Pierce™ C# 24612). The indicated bands in Figure 11A were cut from gels, destained, and submitted to the IUB Mass Spectrometry facility for Trypsin digests and Mass Spectrometry as described previously (Sham *et al.,* 2011). Results from mass spectrometry indicated that Spd_1350 (ZapJ) peptides were not detected in ZapA$^+$ control sample and were the most enriched peptides in the ZapA-FLAG sample compared to the ZapA$^+$ control.

2. SUPPLEMENTARY TABLES

Supplementary Table 1. *S. pneumoniae* bacterial strains used in this study[a b c e]

Strain number	Genotype (description)[b c]	Antibiotic resistance[d]	Reference or source
IU1824	D39 Δ*cps rpsL1*	Str^R	(Lanie *et al.,* 2007)
IU1945	D39 Δ*cps*	None	(Lanie *et al.,* 2007)
IU3116	D39 *rpsL1* CEP*::*P_c-[*kan-rpsL*$^+$]	Kan^R	(Ramos-Montanez *et al.,* 2010)
IU4352	D39 *rpsL1* CEP::P_{fcsK}-*ackA*$^+$	Str^R	(Ramos-Montanez *et al.,* 2010)
IU4355	*D39 rpsL1* Δ*cps* Δ*bgaA'*::P_c-*kan*t1t2-P_{fcsk}-*secA*-L-FLAG^3	Str^r Kan^r	(Tsui *et al.,* 2011)
IU4368	D39 Δ*cps ftsZ*-FLAG^3-P_c-*erm*	Erm^R	(Tsui *et al.,* 2011)
IU5122	D39 Δ*cps rpsL1* CEP*::*P_c-[*kan-rpsL*$^+$] (IU1824 transformed with CEP*::*P_c-[*kan-rpsL*$^+$] from IU3116)	Kan^R	This Study
IU5456	D39 Δ*cps ezrA*-L_0-FLAG^3-P_c-*erm*	Erm^R	(Rued *et al.,* 2016)

IU5544	D39 Δ*cps pbp1a*-L_0-$FLAG^3$-P_c-*erm*	Erm^R	(Land *et al.,* 2013)
IU5557	D39 Δ*cps bgaA'*::*kan*-t1t2-P_{fcsK}-*ftsZ*$^+$ (IU1945 transformed with fusion amplicon *bgaA'*::*kan*-t1t2-P_{fcsK}-*ftsZ*$^+$)	Kan^R	This Study
IU5653	D39 Δ*cps divIVA*-L_0-$FLAG^3$-P_c-*erm* (IU1945 transformed with fusion amplicon *divIVA*-L_0-$FLAG^3$-P_c-*erm*)	Erm^R	This Study
IU5781	D39 Δ*cps bgaA'*::*kan*-t1t2-P_{fcsK}-*ezrA*$^+$ (IU1945 transformed with fusion amplicon *bgaA'*::*kan*-t1t2-P_{fcsK}-*ezrA*$^+$)	Kan^R	This Study
IU5795	D39 Δ*cps* Δ*ezrA*<>*aad9*//*bgaA'*::*kan*-t1t2-P_{fcsK}-*ezrA*$^+$ (IU5781 transformed with fusion amplicon, Δ*ezrA*<>*aad9*; strains were plated and stored in the presence of 1% w/v fucose)	Kan^R Spc^R	This Study
IU6545	D39 Δ*cps ezrA*-HA-P_c-*erm* (IU1945 transformed with fusion amplicon *ezrA*-HA-P_c-*erm*)	Erm^R	This Study
IU6565	D39 Δ*cps ftsZ*-FLAG-*Pc*-*erm* (IU1945 transformed with fusion amplicon *ftsZ*-FLAG-*Pc*-erm)	Erm^R	This Study
IU6570	D39 Δ*cps ftsZ*-Myc-P_c-*erm*	Erm^R	(Land *et al.,* 2013)
IU6810	D39 Δ*cps ezrA*-HA-P_c-*kan*	Kan^R	(Rued *et al.,* 2016)
IU6929	D39 Δ*cps pbp2x*-HA-P_c-*kan*	Kan^R	(Tsui *et al.,* 2014)
IU6933	D39 Δ*cps pbp2b*-HA-P_c-*kan*	Kan^R	(Tsui *et al.,* 2014)
IU6962	D39 Δ*cps ftsZ*-Myc-P_c-*kan*	Kan^R	(Land *et al.,* 2013)
IU7054	D39 Δ*cps bgaA'*::*kan-t1t2*-P_{ftsA}-RBS^{ftsA}-*ftsZ*$^+$	Kan^R	(Perez *et al.,* 2019)
IU7070	D39 Δ*cps ftsZ*-Myc-P_c-*kan ezrA*-L_0-$FLAG^3$-P_c-*erm* (IU6962 transformed with *ezrA*-L_0-$FLAG^3$-P_c-*erm* amplicon from IU5456)	Erm^R Kan^R	This Study
IU7223	D39 Δ*cps ezrA*-HA-P_c-*kan ftsZ*-Myc-P_c-*erm* (IU6810 transformed with *ftsZ*-Myc-P_c-*erm* amplicon from IU6570)	Erm^R Kan^R	This Study
IU7334	D39 Δ*cps rpsL1* CEP::P_{fcsK}-*ezrA*$^+$ (IU5122 transformed with fusion amplicon CEP::P_{fcsK}-*ezrA*$^+$)	Str^R	This Study
IU7351	D39 Δ*cps sepF*-HA-P_c-*kan* (IU1945 transformed with fusion amplicon *sepF*-HA-P_c-*kan*)	Kan^R	This Study
IU7353	D39 Δ*cps sepF*-FLAG-P_c-*erm* (IU1945 transformed with fusion amplicon *sepF*-FLAG-P_c-*erm*)	Erm^R	This Study
IU7438	D39 Δ*cps stkP*-HA-P_c-*kan*	Kan^R	(Tsui *et al.,* 2016)

IU7614	D39 Δ*cps rpsL1 ftsZ*$^+$-P$_c$-[*kan-rpsL*$^+$]	Kan^R	(Tsui *et al.*, 2016)
IU7654	D39 Δ*cps ftsK*-FLAG^2-P$_c$-*erm* (IU1945 transformed with fusion amplicon *ftsK*-FLAG^2-P$_c$-*erm*)	Erm^R	This Study
IU7655	D39 Δ*cps ftsK*-HA^2-P$_c$-*kan* (IU1945 transformed with fusion amplicon *ftsK*- HA^2-P$_c$-*kan*)	Kan^R	This Study
IU7667	D39 Δ*cps rpsL1 ftsZ*-Myc	Str^R	(Mura *et al.*, 2016)
IU7814[d]	D39 Δ*cps* Δ*ftsZ*::*aad9*//*bgaA*'::*kan*-t1t2-P$_{ftsA}$-RBS^{ftsA}-*ftsZ*$^+$ (IU7054 transformed with fusion amplicon Δ*ftsZ*::*aad9*)	Kan^R Spc^R	This Study
IU7933	D39 Δ*cps rpsL1* Δ*zapA*::P$_c$-[*kan-rpsL*$^+$] (IU1824 transformed with Δ*zapA*::P$_c$-[*kan-rpsL*$^+$] amplicon from K743)	Kan^R	This Study
IU8033	D39 Δ*cps rpsL1* Δ[*zapA-spd_0370*]::P$_c$-[*kan-rpsL*$^+$] *ftsZ*-Myc markerless (IU7667 transformed with Δ[*zapA-spd_0370*::P$_c$-[*kan-rpsL*$^+$] from K747)	Kan^R	This Study
IU8035	D39 Δ*cps rpsL1* Δ*zapA* markerless (IU7933 transformed with fusion amplicon *ΔzapA* markerless)	Str^R	This study
IU8122	D39 Δ*cps bgaA*'::*tet*-P$_{Zn}$-RBS^{ftsA}-*ftsZ*$^+$ (IU1945 transformed with fusion amplicon *bgaA*'::*tet*-P$_{Zn}$-RBS^{ftsA}-*ftsZ*$^+$)	Tet^R	This Study
IU8124[d]	D39 Δ*cps* Δ*ftsZ*::*aad9*//*bgaA*'::*tet*-P$_{Zn}$-RBS^{ftsA}-*ftsZ*$^+$ (IU7814 transformed with amplicon *bgaA*'::*tet*-P$_{Zn}$-RBS^{ftsA}-*ftsZ*$^+$ from IU8122)	Spc^R Tet^R	This Study
IU8191	D39 Δ*cps ezrA*-HA-P$_c$-*kan bgaA*'::*tet*-P$_{Zn}$-RBS^{ftsA}-*ftsZ*-Myc (IU6810 transformed with fusion amplicon *bgaA*'::*tet*-P$_{Zn}$-RBS^{ftsA}-*ftsZ*-Myc)	Kan^R Tet^R	This Study
IU8237[d]	D39 Δ*cps ezrA*-HA-P$_c$-*kan* Δ*ftsZ*::*aad9*//*bgaA*'::*tet*-P$_{Zn}$-RBS^{ftsA}-*ftsZ*-Myc (IU8191 transformed with Δ*ftsZ*::*aad9* from IU7814)	Kan^R Spc^R Tet^R	This Study
IU8596	D39 Δ*cps rpsL1 ftsZ*-Myc *sepF*-HA-P$_c$-*kan* (IU7667 transformed with *sepF*-HA-P$_c$-*kan* from IU7351)	Kan^R Str^R	This Study
IU8681	D39 Δ*cps rpsL1 ftsZ*-Myc *ezrA*-L$_0$-FLAG^3-P$_c$-*erm*	Erm^R Str^R	(Rued *et al.*, 2016)
IU8793	D39 Δ*cps bgaA*'::*tet*-P$_{Zn}$-RBS^{ftsA}-*ezrA*-L$_0$-FLAG^3 (IU1945 was transformed with fusion amplicon *bgaA*'::*tet*-P$_{Zn}$-RBS^{ftsA}-*ezrA*-L$_0$-FLAG^3)	Tet^R	This Study
IU8795	D39 Δ*cps bgaA*'::*tet*-P$_{Zn}$-RBS^{ftsA}-*ezrA* (IU1945 transformed with fusion amplicon *bgaA*'::*tet*-P$_{Zn}$-RBS^{ftsA}-*ezrA*$^+$)	Tet^R	This Study
IU8799[d]	D39 Δ*cps* Δ*ezrA*<>*aad9*//*bgaA*'::*tet*-P$_{Zn}$-RBS^{ftsA}-*ezrA*$^+$ (IU8795 transformed with Δ*ezrA*<>*aad9* amplicon from IU5795)	Spc^R Tet^R	This Study

IU8810[d]	D39 Δ*cps* Δ*lytA*::P_c-*erm* Δ*ftsZ*::*aad9*//*bgaA'*::*tet*-P_{Zn}-RBS^{ftsA}-*ftsZ*$^+$ (IU8124 transformed with Δ*lytA*::P_c-*erm* amplicon from E42)	Erm^R Spc^R Tet^R	This Study
IU8845	D39 Δ*cps rpsL1 ftsZ*-L_2-*gfp* (IU7614 transformed with fusion amplicon *ftsZ*-L_2-*gfp*)	Str^R	This Study
IU8902	D39 Δ*cps rpsL1 ftsZ*-L_2-*gfp bgaA'*::*tet*-P_{Zn}-RBS^{ftsA}-*ezrA*$^+$ (IU8845 transformed with fusion amplicon *bgaA'*::*tet*-P_{Zn}-RBS^{ftsA}-*ezrA*$^+$)	Str^R Tet^R	This Study
IU8906	D39 Δ*cps rpsL1 bgaA'*::*tet*-P_{Zn}-RBS^{ftsA}-*ezrA*$^+$ (IU1824 transformed with fusion amplicon *bgaA'*::*tet*-P_{Zn}-RBS^{ftsA}-*ezrA*$^+$)	Str^R Tet^R	This Study
IU8908[d]	D39 Δ*cps rpsL1 ftsZ*-L_2-*gfp* Δ*ezrA*<>*aad9*//*bgaA'*::*tet*-P_{Zn}-RBS^{ftsA}-*ezrA*$^+$(IU8902 transformed Δ*ezrA*<>*aad9* amplicon from IU5795)	Spc^R Str^R Tet^R	This Study
IU9020	D39 Δcps *rpsL1 gfp*-L_1-*pbp2x*	Str^R	(Perez *et al.*, 2019)
IU9077	D39 Δ*cps rpsL1 ezrA*$^+$-P_c-[*kan-rpsL*$^+$]	Kan^R	(Perez *et al.*, 2019)
IU9085	D39 Δ*cps* Δ*mapZ*::P_c-*erm* (IU1945 transformed with fusion amplicon Δ*mapZ*::P_c-*erm*)	Erm^R	This Study
IU9086	D39 Δ*cps rpsL1* Δ*mapZ*::P_c-[*kan-rpsL*$^+$]	Kan^R	(Perez *et al.*, 2019)
IU9090	D39 Δ*cps rpsL1 ftsZ*-Myc *mapZ*-L_0-FLAG^3-P_c-*erm*	Erm^R Str^R	(Perez *et al.*, 2019)
IU9092	D39 Δ*cps rpsL1 ftsZ*-Myc Δ*mapZ*::P_c-[*kan-rpsL*$^+$] (IU7667 transformed with Δ*mapZ*::P_c-[*kan-rpsL*$^+$] amplicon from IU9086)	Kan^R	This Study
IU9094	D39 Δ*cps rpsL1* P_c-[*kan-rpsL*$^+$]-*mapZ*	Kan^R	(Perez *et al.*, 2019)
IU9097	D39 Δ*cps rpsL1 ftsZ*-L_2-*gfp* Δ*mapZ*::P_c-[*kan-rpsL*$^+$]	Kan^R	(Perez *et al.*, 2019)
IU9175	D39 Δ*cps rpsL1* Δ*mapZ*	Str^R	(Boersma *et al.*, 2015)
IU9182	D39 Δ*cps rpsL1 gfp*-L_1-*mapZ*	Str^R	(Perez *et al.*, 2019)
IU9207	D39 Δ*cps ezrA*-HA-P_c-*kan mapZ*-L_0-FLAG^3-P_c-*erm*	Kan^R Erm^R	(Perez *et al.*, 2019)
IU9548[d]	D39 Δ*cps* Δ*mapZ*::P_c-*erm* Δ*ezrA*<>*aad9*//*bgaA'*::*tet*-P_{Zn}-RBS^{ftsA}-*ezrA*$^+$ (IU8799 transformed with Δ*mapZ*::P_c-*erm* amplicon from IU9085)	Erm^R Spc^R Tet^R	This Study
IU9550[d]	D39 Δ*cps* Δ*sepF*::P_c-*erm* Δ*ezrA*<>*aad9*//*bgaA'*::*tet*-P_{Zn}-RBS^{ftsA}-*ezrA*$^+$ (IU8799 transformed with Δ*sepF*::P_c-*erm* amplicon from E733)	Erm^R Spc^R Tet^R	This Study
IU9552[d]	D39 Δ*cps* Δ[*zapA(spd_0369)-spd_0370*]::P_c-*erm* Δ*ezrA*<>*aad9*//*bgaA'*::*tet*-P_{Zn}-RBS^{ftsA}-*ezrA*$^+$ (IU8799	Erm^R Spc^R Tet^R	This Study

	transformed with Δ[*zapA(spd_0369)-spd_0370*]::P_c-*erm* amplicon from E747)		
IU9572[d]	D39 Δ*cps* Δ*ezrA<>aad9*//*bgaA*'::*tet*-P_{Zn}-RBS^{ftsA}-*ezrA*-L_0-FLAG[3] (IU8793 was transformed with Δ*ezrA<>aad9* amplicon from IU5795)	Spc^R Tet^R	This Study
IU9651	D39 Δ*cps rpsL1 ftsZ*-Myc Δ*mapZ* (IU9092 was transformed with Δ*mapZ* amplicon from IU9175)	Str^R	This Study
IU9683	D39 Δ*cps hlpA*-L_5-*sfgfp*-Cm (IU1945 transformed with *hlpA*-L_5-*sfgfp*-Cm amplicon from JWV500)	Cm^R	(Perez *et al.*, 2019)
IU9713	D39 Δ*cps rpsL1 ftsZ*-Myc *ezrA*-HA-P_c-*kan* (IU7667 transformed with *ezrA*-HA-P_c-*kan* amplicon from IU6810)	Kan^R Str^R	This Study
IU9723	D39 Δ*cps rpsL1 ftsZ*-Myc *ezrA*-HA-P_c-*kan* Δ*mapZ* (IU9651 transformed with *ezrA*-HA-P_c-*kan* amplicon from IU6810)	Kan^R Str^R	This Study
IU9767	D39 Δ*cps rpsL1* P_c-[*kan-rpsL*$^+$]-*ftsA*$^+$	Kan^R	(Mura *et al.*, 2016)
IU9805	D39 Δ*cps bgaA*::*kan*-t1t2-P_{Zn}-*sepF*$^+$	Kan^R	(Perez *et al.*, 2020)
IU9881	D39 Δ*cps rpsL1 ftsZ*-L_2-*gfp* Δ*mapZ*	Str^R	(Perez *et al.*, 2019)
IU9967	D39 Δ*cps rpsL1* HA-*ftsA*	Str^R	(Rued *et al.*, 2016)
IU9969	D39 Δ*cps rpsL1* FLAG-*ftsA*	Str^R	(Mura *et al.*, 2016)
IU9985	D39 Δ*cps rpsL1 ftsZ*-L_2-*sfgfp*	Str^R	(Perez *et al.*, 2019)
IU10035	D39 Δ*cps rpsL1 gfp*-L_2-*ftsA*	Str^R	(Perez *et al.*, 2019)
IU10065	D39 Δ*cps rpsL1 zapA*-L_4-*sfgfp* (Strain IU7933 transformed with fusion amplicon *zapA*-L_4-*sfgfp)*	Str^R	This Study
IU10234	D39 Δ*cps rpsL1* HA-*ftsA*-*ftsZ*-P_c-[*kan-rpsL*$^+$] (IU9967 transformed with *ftsZ*-P_c-[*kan-rpsL*$^+$] amplicon from IU7614).	Kan^R	This Study
IU10236	D39 Δ*cps rpsL1* FLAG-*ftsA*-*ftsZ*-P_c-[*kan-rpsL*$^+$]	Kan^R	(Mura *et al.*, 2016)
IU10254	D39 Δ*cps rpsL1 ezrA*-L_0-*sfgfp*	Str^R	(Perez *et al.*, 2019)
IU10265	D39 Δ*cps zapA*-L_4-FLAG (IU7933 transformed with fusion amplicon *zapA*-L_4-FLAG)	Str^R	This Study
IU10267	D39 Δ*cps zapA*-L_4-HA (IU7933 transformed with fusion amplicon *zapA*-L_4-HA)	Str^R	This Study
IU10302	D39 Δ*cps rpsL1* HA-*ftsA fts*Z-Myc (IU10234 transformed with *fts*Z-Myc amplicon from IU7667)	Str^R	This Study
IU10304	D39 Δ*cps rpsL1* FLAG-*ftsA fts*Z-Myc	Str^R	(Mura *et al.*, 2016)

IU10447	D39 Δ*cps ezrA*$^+$-P_c-*erm* (IU1945 transformed with fusion amplicon *ezrA*$^+$-P_c-*erm*)	Erm^R	This Study
IU10449	D39 Δ*cps rpsL1 ezrA*-L_0-*gfp*	Str^R	(Perez *et al.*, 2019)
IU10526	D39 Δ*cps rpsL1 ezrA*-L_0-*gfp* Δ*mapZ*::P_c-[*kan-rpsL*$^+$] (IU10449 transformed with Δ*mapZ*::P_c-[*kan-rpsL*$^+$] amplicon from IU9086)	Kan^R	This Study
IU10540	D39 Δ*cps rpsL1 ezrA*-L_0-*gfp* Δ*mapZ* (IU10526 transformed with Δ*mapZ* amplicon from IU9175)	Str^R	This Study
IU10752	D39 Δ*cps ftsZ*-Myc *zapA*-L_4-FLAG (IU8033 transformed with amplicon *zapA*-L_4-FLAG amplicon from IU10265)	Str^R	This Study
IU10839[d]	D39 Δ*cps* Δ*zapA*::P_c-*erm* Δ*ezrA*<>*aad9*//*bgaA*'::*tet*-P_{Zn}-RBS^{ftsA}-*ezrA* (IU8799 transformed with Δ*zapA*::P_c-*erm* amplicon from E743)	Erm^R $Spec^R$ Tet^R	This Study
IU10843[d]	D39 Δ*cps* Δ*zapA*::P_c-*erm* Δ*ftsZ*::*aad9*//*bgaA*'::*tet*-P_{Zn}-RBS^{ftsA}-*ftsZ*$^+$ (IU8124 transformed with Δ*zapA*::P_c-*erm* amplicon from E743)	Spc^R Tet^R	This Study
IU10901[d]	D39 Δ*cps ezrA*(QND)-P_c-*erm*//*bgaA*'::*tet*-P_{Zn}-RBS^{ftsA}-*ezrA*$^+$ (IU8799 transformed with fusion amplicon *ezrA*(QND)-P_c-*erm*)	Erm^R Tet^R	This Study
IU10909[d]	D39 Δ*cps ezrA*ΔQNR-P_c-*erm*//*bgaA*'::*tet*-P_{Zn}-RBS^{ftsA}-*ezrA*$^+$ (IU8799 transformed with fusion amplicon *ezrA*ΔQNR-P_c-*erm*)	Erm^R Tet^R	This Study
IU11119	D39 Δ*cps rpsL1 ezrA*-L_0-*sfgfp*-P_c-*cat*	Str^R Cm^R	(Perez *et al.*, 2019)
IU11123[d]	D39 Δ*cps ezrA*ΔTM-P_c-*erm*//*bgaA*'::*tet*-P_{Zn}-RBS^{ftsA}-*ezrA* (IU8799 transformed with fusion amplicon *ezrA*ΔTM-P_c-*erm*)	Erm^R Tet^R	This Study
IU11230	D39 Δ*cps rpsL1* FLAG-*fts*A//*bgaA*'::*tet*-P_{Zn}-*ftsZ*-Myc (IU9969 transformed with amplicon from IU8191)	Str^R Tet^R	This study
IU11322	D39 Δ*cps rpsL1 zapA*-L_4-HA *ftsZ*-FLAG-P_c-*erm* (IU10267 transformed with amplicon *ftsZ*-FLAG-P_c-*erm* from IU6565)	Erm^R Str^R	This Study
IU11356	*D39* Δ*cps rpsL1* FLAG-*ftsA* Δ*ftsZ*::*aad9*//*bgaA*'::*tet*-P_{Zn}-RBS_{ftsA}-*ftsZ*-Myc$^+$ (IU11230 transformed with Δ*ftsZ*::*aad9* amplicon from IU7814)	$Spc^R$$Str^R$ Tet^R	This study
IU11414	D39 Δ*cps rpsL1 ftsZ*-Myc *ezrA*-HA-P_c-*kan divIVA*-L_0-$FLAG^3$-P_c-*erm* (IU9713 transformed with *divIVA*-L_0-$FLAG^3$-P_c-erm amplicon from IU5653)	Erm^R Str^R	This Study
IU11430	D39 Δ*cps rpsL1 ftsZ*-Myc *ezrA*-HA-P_c-*kan mapZ*-L_0-$FLAG^3$-P_c-*erm* (IU9713 transformed with *mapZ*-L_0-$FLAG^3$-P_c-*erm* amplicon from IU9090)	Erm^R Str^R	This Study
IU11476	D39 Δ*cps rpsL1* FLAG-*ftsA ftsZ*-Myc *ezrA*-HA-P_c-*kan* (IU10304 was transformed with *ezrA*-HA-P_c-*kan* amplicon from IU6810)	Kan^R Str^R	This Study

IU11558	D39 Δ*cps divIVA*-Myc-P_c-*kan*	Kan^R	(Rued *et al.*, 2016)
IU11560	D39 Δ*cps pbp2a*-HA^4-P_c-*kan*	Kan^R	(Rued *et al.*, 2016)
IU11610	D39 Δ*cps pbp2a*-HA^4-P_c-*kan ezrA*-L_0-$FLAG^3$-P_c-*erm* (IU11560 transformed with *ezrA*-L_0-$FLAG^3$-P_c-*erm* amplicon from IU5456)	Erm^R Kan^R	This Study
IU11664	D39 Δ*cps rpsL1 ftsZ*-Myc *ezrA*-HA-P_c-*kan ftsK*-$FLAG^2$-P_c-*erm* (IU9713 was transformed with *ftsK*-$FLAG^2$-P_c-*erm* amplicon from IU7654)	Erm^R Kan^R	This Study
IU11734	D39 Δ*cps gpsB*-Myc-P_c-*kan* (IU1945 transformed with fusion amplicon *gpsB*-Myc-P_c-*kan*)	Kan^R	This Study
IU11840	D39 Δ*cps rpsL1 zapA*-L4-FLAG *ezrA*-HA-P_c-*erm* (IU10265 transformed with strain amplicon *ezrA*-HA-P_c-*erm* from IU6545)	Erm^R Str^R	This Study
IU11939	D39 Δ*cps rpsL1 ezrA*-HA-Pc-*kan* (IU1824 transformed with strain amplicon *ezrA*-HA-Pc-*kan* from IU6810)	Kan^R Str^R	This Study
IU11978	D39 Δ*cps gpsB*-Myc-P_c-*kan ezrA*-L_0-$FLAG^3$-P_c-*erm* (IU11734 transformed with *ezrA*-L_0-$FLAG^3$-P_c-*erm* amplicon from IU5456)	Erm^R Kan^R	This Study
IU12069	D39 Δ*cps pbp1a*-L_0-$FLAG^3$-P_c-*erm ezrA*-HA-P_c-*kan* (IU5544 transformed with *ezrA*-HA-P_c-*kan* amplicon from IU6810)	Erm^R Kan^R	This Study
IU12076	D39 Δ*cps sepF*-FLAG-P_c-*erm ezrA*-HA-P_c-*kan* (IU7353 transformed with *ezrA*-HA-P_c-*kan* amplicon from IU6810)	Erm^R Kan^R	This Study
IU12077	D39 Δ*cps stkP*-$FLAG^2$-P_c-*erm ezrA*-HA-P_c-*kan* (IU7434 transformed with *ezrA*-HA-P_c-*kan* amplicon from IU6810)	Erm^R Kan^R	This Study
IU12253	D39 Δ*cps rpsL1 zapA*-L_4-*sfgfp*-P_c-*aad9* (IU1824 transformed with fusion amplicon *zapA*-L_4-*sfgfp*-P_c-*aad9*)	Spc^R Str^R	This Study
IU13123	D39 Δ*cps rpsL1 ezrA*$^+$//CEP::P_{Zn}-*ezrA*$^+$ (IU5122 transformed with fusion amplicon CEP::t1t2::P_{Zn}-*ezrA*$^+$)	Str^R	This Study
IU13189[d]	D39 Δ*cps ezrA(QND)*-L_0-*sfgfp*-P_c-*cat//bgaA'*::*tet*-P_{Zn}-*ezrA*$^+$ (IU8799 transformed with fusion amplicon *ezrA(QND)*-L_0-*sfgfp*-P_c-*cat*)	Cm^R Tet^R	This Study
IU13191[d]	D39 Δ*cps ezrA(*ΔQNR*)*-L_0-*sfgfp*-P_c-*cat//bgaA'*::*tet*-P_{Zn}-*ezrA*$^+$ (IU8799 transformed with fusion amplicon *ezrA(*ΔQNR*)*-L_0-*sfgfp*-P_c-*cat*)	Cm^R Tet^R	This Study
IU13194	D39 Δ*cps ezrA*-L_0-*sfgfp*-P_c-*cat//bgaA'*::*tet-*:P_{Zn}-*ezrA*$^+$ (IU8795 transformed with *ezrA*-L_0-*sfgfp*-P_c-*cat* from IU11119)	Cm^R Tet^R	This Study

IU13269[d]	D39 Δ*cps ezrA(*ΔTM*)*-L$_0$-*sfgfp*-P$_c$-*cat//bgaA'::*P$_{Zn}$-*ezrA*$^+$ (IU8799 transformed with fusion amplicon *ezrA(*ΔTM*)*-L$_0$-*sfgfp*-P$_c$-*cat*)	Cm^R Tet^R	This Study
IU13327	D39 Δ*cps rpsL1 ezrA*$^+$//CEP::P$_{Zn}$-*ezrA*$^+$//*bgaA'*::kan-t1t2-P$_{Zn}$-*ezrA*$^+$ (IU13123 transformed with fusion amplicon *bgaA'*::kan-t1t2-P$_{Zn}$-*ezrA*$^+$)	Kan^R Str^R	This Study
IU13406	D39 Δ*cps rpsL1 ftsZ*-L$_5$-*cfp-erm*	Erm^R Cm^R	(Perez *et al.,* 2019)
IU13700	D39 Δ*cps rpsL1 ftsZ-cfp ezrA*$^+$//CEP::P$_{Zn}$-*ezrA*$^+$//*bgaA'*::P$_{Zn}$-*ezrA*$^+$ (IU13327 transformed with *ftsZ*-L$_5$-*cfp-erm* from IU13406)	Erm^R Kan^R Str^R	This Study
IU13822	D39 Δ*cps rpsL1 zapJ-L$_0$-sfgfp*-P$_c$-*cat* (IU1824 transformed with fusion amplicon *zapJ-L$_0$-sfgfp*-P$_c$-*cat)*	Str^R Cm^R	This Study
IU13922	D39 Δ*cps ΔzapJ(spd_1350)*::P$_c$-[*kan-rpsL*$^+$] (IU1945 transformed with fusion amplicon *ΔzapJ(spd_1350)*::P$_c$-[*kan-rpsL*$^+$]	Kan^R	This Study
IU13924	D39 Δ*cps ΔzapJ(spd_1350)*::P$_c$-*erm* (IU1945 transformed with fusion amplicon *ΔzapJ(spd_1350)*::P$_c$-*erm*)	Erm^R	This Study
IU14109	D39 Δ*cps rpsL1 ΔzapA markerless ftsZ*-P$_c$-[*kan-rpsL*+] (IU8035 transformed with *ftsZ*-P$_c$-[*kan-rpsL*+] from IU7614)	Kan^R	This Study
IU14131	D39 *Δcps ftsZ*-L$_2$-*sfgfp ΔzapA markerless* (IU14109 transformed with amplicon *ftsZ*-L2-*sfgfp* from IU9985)	Str^R	This Study
IU14153	D39 Δ*cps rpsL1 ftsZ*-L$_5$-*cfp-erm ezrA-mNeonGreen*-P$_c$-*cat* (IU13406 transformed with *ezrA-mNeonGreen*-P$_c$-*cat* from IU14117*)*	Erm^R Cm^R	This Study
IU14224	D39 Δ*cps rpsL1 ftsZ-L$_2$-sfgfp bgaA*'::*tet*-P$_{Zn}$-RBS^{ftsA}-*ezrA*$^+$ (IU9985 transformed with *bgaA*'::*tet*-P$_{Zn}$-RBS^{ftsA}-*ezrA*$^+$ from IU8795)	Str^R Tet^R	This Study
IU14404	D39 Δ*cps rpsL1 ezrA*-L$_0$-*ht*-P$_c$-*erm*	Erm^R Str^R	(Perez *et al.,* 2019)
IU15012	D39 Δ*cps rpsL1* Δ*zapJ(spd_1350)*::P$_c$-[*kan-rpsL*$^+$] (IU1824 transformed with Δ*zapJ(spd_1350)*::P$_c$-[*kan-rpsL*$^+$] from IU13922)	Kan^R	This Study
IU15025	D39 Δ*cps rpsL1 zapJ*-L$_0$-*ht*-P$_c$-*erm* (IU1824 transformed with fusion *zapJ*-L$_0$-*ht*-P$_c$-*erm*)	Erm^R Str^R	This Study
IU15029[d]	D39 Δ*cps ΔzapJ*::P$_c$-*erm ΔezrA<>aad9//bgaA*'::*tet*-P$_{Zn}$-*ezrA*$^+$ (IU8799 was transformed with Δ*zapJ*::P$_c$-*erm* from IU13924)	Erm^R Spc^R Tet^R	This Study
IU15100	D39 Δ*cps rpsL1* Δ*mapZ*::P$_c$-*erm* (IU1824 transformed with Δ*mapZ*::P$_c$-*erm* from IU9085)	Erm^R	This Study
IU15107	D39 Δ*cps rpsL1* Δ*zapA* Δ*mapZ*::P$_c$-*erm* (IU8035 transformed with Δ*mapZ*::P$_c$-*erm* from IU9085)	Erm^R	This Study

IU15110	D39 Δ*cps rpsL1* Δ*zapJ(spd_1350)*::P_c-[*kan-rpsL*$^+$] Δ*mapZ*::P_c-*erm* (IU15012 transformed with Δ*mapZ*::P_c-*erm* from IU9085)	Kan^R Erm^R	This Study
IU15116	D39 Δ*cps rpsL1 zapJ*-L_0-*ht*-P_c-*erm zapA*-L_4-*sfgfp*-P_c-*aad9* (IU15025 transformed with *zapJ*-L_0-*ht*-P_c-*erm* from IU12253)	Erm^R Spc^R Str^R	This Study
IU15699	D39 Δ*cps rpsL1 gfp*-L_2-*ftsA ezrA*-L_0-*ht*-P_c-*erm* (IU10035 transformed with *ezrA*-L_0-*ht*-P_c-*erm* from IU14404)	Erm^R Str^R	This Study
IU15768	D39 Δ*cps rpsL1 ftsZ*-L_2-*sfgfp ezrA*-L_0-*ht*-P_c-*erm* (IU9985 transformed with *ezrA*-L_0-*ht*-P_c-*erm* from IU14404)	Str^R Erm^R	This Study
E42	D39 Δ*cps* Δ*lytA*::P_c-*erm* (IU1945 transformed with fusion Δ*lytA*::P_c-*erm*)	Erm^R	This Study
E733	D39 Δ*cps* Δ*sepF(spd_1477)*::P_c-*erm* (IU1945 transformed with fusion amplicon Δ*sepF(spd_1477)*::P_c-*erm*)	Erm^R	This Study
E743	D39 Δ*cps* Δ*zapA*(*spd_0369*)::P_c-*erm* (IU1945 transformed with fusion amplicon Δ*zapA*(*spd_0369*)::P_c-*erm*)	Erm^R	This Study
E745	D39 Δ*cps* Δ*spd_0370*::P_c-*erm* (IU1945 transformed with fusion amplicon Δ*spd_0370*::P_c-*erm*	Erm^R	This Study
E747	D39 Δ*cps* Δ[*zapA(spd_0369)-spd_0370*]::P_c-*erm* (IU1945 transformed with fusion amplicon Δ[*zapA-spd_0370*]::P_c-[*kan-rpsL*$^+$])	Erm^R	This Study
K743	D39 Δ*cps* Δ*zapA*(*spd_0369*)::P_c-[*kan-rpsL*$^+$] (IU1945 transformed with fusion amplicon Δ*zapA*(*spd_0369*)::P_c-[*kan-rpsL*$^+$])	Erm^R	This Study
K747	D39 Δ*cps* Δ[*zapA-spd_0370*]::P_c-[*kan-rpsL*$^+$] (IU1945 transformed with fusion amplicon Δ[*zapA-spd_0370*]::P_c-[*kan-rpsL*$^+$])	Kan^R	This Study
JWV500	*hlpA*-L_5-*sfgfp*-Cm	Cm^R	(Kjos *et al.,* 2015)

[a]Strains were constructed as described in *Materials and Methods* and above.

[b]Primers used to synthesize fusion amplicons are listed in **Supplementary Table 2**.

[c]Linkers and tags are annotated as described below. FLAG-tagged (FLAG), c-Myc-tagged (Myc), and HA-tagged (HA) fusions were made to the carboxyl-end of all tagged proteins. The amino acid sequences of the FLAG, Myc, and HA epitope tags are DYKDDDDK (Hopp *et al.,* 1988, Wayne *et al.,* 2010), EQKLISEEDL (Evan *et al.,* 1985), and YPYDVPDYA (Wilson *et al.,* 1984), respectively. $FLAG^n$ indicates n tandem sequences of the FLAG epitope, DYKDDDDK. L_0 for to a 10-amino-acid spacer linker (GSAGSAAGSG) (Waldo *et al.,* 1999; Wayne *et al.,* 2010)). L_1 linker sequence in *gfp*-L_1-*mapZ* is LEGSG (Fleurie *et al.,* 2014). The DNA template for *gfp* is pUC57-*gfp(Sp)* (Martin *et al.,* 2010), which was codon optimized for *S. pneumoniae* and contains aa substitution (A206K) to prevent GFP dimerization. L_2-linker sequence in *ftsZ*-L_2-*gfp* is KLDIEFLQ (Fleurie *et al.,* 2014). Superfolder GFP (*sfgfp*) is from (Kjos *et al.,* 2015). *rfp* referred to as mKate2 and is a far red

monomeric fluorescent protein with codon optimized for *S. pneumoniae* (Beilharz *et al.,* 2015). L_4 sequence in ZapA tagged proteins is RSIAT (Pazos *et al.,* 2013). L_5 sequence in HlpA tagged proteins is GSGSGGEAAAKGS (Kjos *et al.,* 2015). HaloTag (*ht*) is codon optimized for *S. pneumoniae* (Perez *et al.,* 2019). FtsZ-L_5-CFP-*erm* is from (van Raaphorst *et al.,* 2017).

[d]The indicated strains were constructed and grown in the presence of 0.3 mM $ZnCl_2$ and 0.03 mM $MnSO_4$ (for *ftsZ* conditional mutants) or 0.5 mM $ZnCl_2$ and 0.05 mM $MnSO_4$ (for *ezrA* conditional mutants).

[e]Antibiotic resistance markers: Erm^R, erythromycin; Kan^R, kanamycin; Spc^R, spectinomycin; Str^R, streptomycin; Cm^R, chloramphenicol; Tet^R, tetracycline.

Supplementary Table 2 Oligonucleotide primers used to construct *S. pneumoniae* strains in this study

Primer	Sequence (5' to 3')	Template[a]	Amplicon Product
**For construction of IU5557 (*bgaA'*::*kan*-t1t2-P$_{fcsK}$-*ftsZ*$^{+}$)**			
TT657	CGCCCCAAGTTCATCACCAATGACATCAAC	IU4888[a]	*bgaA'*-P$_{c}$-*kan*-t1t2-P$_{fcsk}$
TT201	CAGCTGTATCAAATGAAAATGTCATTTTTCTT CTCTCTTCGTCCTTGATTAACTT		
TT202	ATCAAGGACGAAGAGAGAAGAAAAATGACA TTTTCATTTGATACAGCTGCTG	D39	*ftsZ*$^{+}$
TT203	ACTGGTTTATGAGAAAGTAAGTTCTTTTATTA ACGATTTTTGAAAAATGGAGGTGTATC		
TT396	CCTCCATTTTTCAAAAATCGTTAGAAGAACTT ACTTTCTCATAAACCAGTTGCTG	D39	*bgaA'*
CS121	GCTTTCTTGAGGCAATTCACTTGGTGC		
**For construction of IU5653 (*divIVA*-L$_{0}$-FLAG^{3}-P$_{c}$-*erm)***			
SC219	TAACCGTCCAGTTATTATTAAGTAAGTGAGG AATAGAATGCCAATTACATCATTAG	D39	*divIVA*
TT244	CGGAGCCAGCGGAACCCTTCTGGTTCTTCAT ACATTGGGCC		
TT245	CCAATGTATGAAGAACCAGAAGGGTTCCGCT GGCTCCGC	IU5456	L$_{0}$-FLAG^{3}-P$_{c}$-*erm*
TT246	TGTCGGATGCACTGGAGCTATTATTTCCTCCC GTTAAATAATAGATAACTATTAAAA		
TT247	TTATCTATTATTTAACGGGAGGAAATAATAG CTCCAGTGCATCCGACAGG	D39	3' flanking downstream of *divIVA*
TT248	TTCAGCAAGGGCTGACTCAGATGACCATGA		
**For construction of IU5781 (*bgaA'*::*kan*-t1t2-P$_{fcsK}$-*ezrA*$^{+}$)**			
TT657	CGCCCCAAGTTCATCACCAATGACATCAAC	IU4888[a]	*bgaA'-kan*-t1t2-P$_{fcsk}$
AL307	CCATTAGACATTTTTCTTCTCTCTTCGT CCTTG		
AL306	GAAGAGAGAAGAAAAATGTCTAATGGACAA C	D39	*ezrA*$^{+}$
AL309b	GAGAAAGTAAGTTCTTTTATTAAAAACGAAT CGTTTCACGTGTTTTCTC		
AL308b	GAAACGATTCGTTTTTAATAAAAGAACTTAC TT TCTCATAAACCAGTTGC	D39	*bgaA'*
CS121	GCTTTCTTGAGGCAATTCACTTGGTGC		
**For construction of IU5795 (Δ*ezrA*<>*aad9*)[h]**			
AL295	CCCAAATCCACAGTTTGAAGGACAAACG	D39	5' upstream of *ezrA*
AL318	CCTCCTCACATCAAACTCCTTTTTACTTGAA AC		
AL319	GGAGTTTGATGTGAGGAGGATATATTTG	IU4888[a]	*aad9* replaces ORF of *ezrA*
AL321	CTTTTTCTTTTATAATTTTTTAATCTG		
AL320	GATTAAAAAATTATAAAAGAAAAAGATTTT ATTG	D39	downstream of *ezrA*

TT330	GAGGAGTTCGGACTCGACTCTCTCCTTCAAG AA		
**For construction of IU6545 (*ezrA*-HA-P$_c$-*erm*)**			
TT192	ATCGTGTTCCAGCCTTGGTTACGACGCTTT	IU1690	5' *ezrA*-HA
SV005	CCCGGTTAAGCATAATCTGGAACATCATATG GATAAAAACGAATCGTTTCACGTGTTTTC		
SV006	GATTCGTTTTTATCCATATGATGTTCCAGATT ATGCTTAACCGGGCCCAAAATTTGTTTG	IU5456	3' HA-P$_c$-*erm* downstream of *ezrA*
AL297	GGACCTACTCCTATTGGAGCCCAAC		
**For construction of IU6565 (*ftsZ*-FLAG-Pc-*erm*)**			
TT165	AGTGGTGCCGATATGGTCTTCATCACTGCT	IU4368[c]	5' fragment containing *ftsZ*-FLAG
TT369	AAATTTTGGGCCCGGTTATTTATCATCATCAT CTTTATAATCACGATTTTTG		
TT370	CACCTCCATTTTTCAAAAATCGTGATTATAAA GATGATGATGATAAATAACCGGG	IU4368[c]	3' fragment FLAG-Pc-*erm* + downstream
TT166	TCATTGGGAGAGCCGGTTCCTGTGAAGAAT		
**For construction of IU7054 (*bgaA'*::*kan*-t1t2-P$_{ftsA}$-RBS^{ftsA}-*ftsZ*$^+$)**			
P146	TGGCCATTCATCGCTGGTCGTGCTGAAAT	IU6397[c]	*bgaA*'::*kan*-t1t2-P$_{ftsA}$
TT393	CAGCTGTATCAAATGAAAATGTCATTACATC GCTTCCTCTCTATCTTCCAAGT		
TT394	GGAAGATAGAGAGGAAGCGATGTAATGACA TTTTCATTTGATACAGCTGCTG	IU5557	3' flanking containing *ftsZ*$^+$-*bgaA*'
CS121	GCTTTCTTGAGGCAATTCACTTGGTGC		
**For construction of IU7334 (CEP::P$_{fcsK}$-*ezrA*$^+$)**			
KW116	CCGGTAGTGGGAAAACAACTATTGGTCGTGC	IU4352	CEP P$_{fcsk}$
TT221	CATTAAATAAATTAGTTGTCCATTAGACATTT TTCTTCTCTCTTCGTCCTTGATTAACTT		
TT222	ATCAAGGACGAAGAGAGAAGAAAAATGTCT AATGGACAACTAATTTATTTAATGGTTG	D39	*ezrA*$^+$
TT450	GAACACCTTCTCAGCGTTCTTTTTAAAAACGA ATCGTTTCACGTGTTTT		
TT451	CACGTGAAACGATTCGTTTTTAAAAAGAACG CTGAGAAGGTGTTCTTTTT	IU4352	CEP downstream
KW123	GGCTTCTTGTTCAAATTTTCCCATTTGATTCT C		
**For construction of IU7351 (*sepF*-HA-P$_c$-*kan*)**			
TT469	GAGAGAGGAACTGCTGGAAATCTTGCCAGA	D39	*ylmE'-sepF*
TT470	GCATAATCTGGAACATCATATGGATATCGTA CTCTATTTCGCTTCATATCAAAACC		
TT471	TGATATGAAGCGAAATAGAGTACGATATCCA TATGATGTTCCAGATTATGCTTAAC	IU6933	HA-P$_c$-*kan*
TT472	ACGAATTAAAAAAATCATTACTAAAACAATT CATCCAGTAAAATATAATATTTTATTTTC		
TT473	ATTTTACTGGATGAATTGTTTTAGTAATGATT TTTTTAATTCGTATGATTTATAATGCAG	D39	3' downstream of *sepF*
P1478	GTTCCTCCAGCGAAACAGGTATACGACC		

**For construction of IU7353 (*sepF*-FLAG-P$_c$-*erm*)**			
TT469	GAGAGAGGAACTGCTGGAAATCTTGCCAGA	D39	*ylmE'-sepF*
TT476	CGGTTATTTATCATCATCATCTTTATAATCTC GTACTCTATTTCGCTTCATATCAAAACC		
TT477	TGATATGAAGCGAAATAGAGTACGAGATTAT AAAGATGATGATGATAAATAACCGGG	IU5544	FLAG-P$_c$-*erm*
TT480	TCATACGAATTAAAAAAATCATTATTATTTCC TCCCGTTAAATAATAGATAACTATTAAA		
TT481	CTATTATTTAACGGGAGGAAATAATAATGAT TTTTTAATTCGTATGATTTATAATGCAG	D39	3' downstream of *sepF*
P1478	GTTCCTCCAGCGAAACAGGTATACGACC		
**For construction of IU7654 (*ftsK*-FLAG^2-P$_c$-*erm*)**			
TT597	GATTCCAGTCGTGACCAATCCACGCAAAG	D39	5' flanking containing *ftsK*-FLAG^2
TT605	TATAATCTTTATCATCATCATCTTTATAATCT TGTTGTAACACTTTTCGAGGTTTGGTAC		
TT606	CTCGAAAAGTGTTACAACAAGATTATAAAGA TGATGATGATAAAGATTATAAAGATGATG	IU5544	Middle FLAG^2-P$_c$-*erm*
TT607	CTTGGAAAGAAGCTATTTTTTTATTTCCTCCC GTTAAATAATAGATAACTATTAAAAATA		
TT608	TTATCTATTATTTAACGGGAGGAAATAAAAA AATAGCTTCTTTCCAAGTTTGGAG	D39	3' flanking downstream of *ftsK*
TT598	CGCCTCAACATCGACCAAGCCTTTCTTATC		
**For construction of IU7655 (*ftsK*-HA^2-P$_c$-*kan*)**			
TT597	GATTCCAGTCGTGACCAATCCACGCAAAG	D39	5' flanking containing with *ftsK*-HA^2
TT603	GCATAATCTGGAACATCATATGGATATTGTT GTAACACTTTTCGAGGTTTGGTAC		
TT604	AAACCTCGAAAAGTGTTACAACAATATCCAT ATGATGTTCCAGATTATGCTTATCCATAT	IU7426[d]	Middle HA^2-P$_c$-*kan*
TT601	AACTTGGAAAGAAGCTATTTTTCTAAAACAA TTCATCCAGTAAAATATAATATTTTATTT		
TT602	AATATTATATTTTACTGGATGAATTGTTTTAG AAAAATAGCTTCTTTCCAAGTTTGGAGG	D39	3' flanking downstream of *ftsK*
TT598	CGCCTCAACATCGACCAAGCCTTTCTTATC		
**For construction of IU7814 (Δ*ftsZ*::*aad9*)[h]**			
AL366	GGCATGATGGGGGTTCGCCTTGAAATGCG	D39	5' upstream of *ftsZ*
TT204	CGTATGTATTCAAATATATCCTCCTCACAATT TATTTTTCCTCTTTATTCGTCAAACATG		
TT205	TTGACGAATAAAGAGGAAAAATAAATTGTGA GGAGGATATATTTGAATACATACGAACA	IU4888[b]	Middle-*aad9* + extra 9 bp of 3' *mreD*
TT206	CTCGACTGGAGAAACGACTGAATGTCGTTCT TATAATTTTTTAATCTGTTATTTAAA		
TT207	ACAGATTAAAAAATTATAAGAACGACATTC AGTCGTTTCTCCAGTCGAGCG	D39	87bp 3' *ftsZ* + stop + downstream
TT166	TCATTGGGAGAGCCGGTTCCTGTGAAGAAT		
**For construction of IU8035 (*ΔzapA markerless)***			
P1488	TGGAAGCTGATAACCCAGTTCTCGTCCCAGA T	D39	

AJP18	TCTGTTCTTGCTTACAAGTCACAAGGGTTAAC GATTTTTCCCGAATGTAAA		Upstream of *zapA* + 5' 60 bp of *zapA*
AJP19	TTCGGGAAAAAATCGTTAACCCTTGTGACTT GTAAGCAAGAACAGAGCAA	D39	3' 45 bp of *zapA* + stop + downstream
P1489	TTATCTGCTTTGGCAGTCGGAGCCAGTTGT		
**For construction of IU8122 (*bgaA*'::*tet*-P_{Zn}-RBS^{ftsA}-*ftsZ*$^{+}$)**			
TT657	CGCCCCAAGTTCATCACCAATGACATCAAC	IU3966[e]	*bgaA*'::*tet*-P_{Zn}-RBS^{ftsA}
AJP32	ACATCGCTTCCTCTCTATCTTCCTTGTTATAA TAGATTTATGAACACCTTGTTCATTATC		
AJP33	AACAAGGTGTTCATAAATCTATTATAACAAG GAAGATAGAGAGGAAGCGATGTAATGA	IU7054	RBS^{ftsA}-*ftsZ*$^{+}$-*bgaA'*
CS121	GCTTTCTTGAGGCAATTCACTTGGTGC		
**For construction of IU8191 (*bgaA*'::*tet*-P_{Zn}-RBS^{ftsA}-*ftsZ*-Myc)**			
TT657	CGCCCCAAGTTCATCACCAATGACATCAAC	IU3966[e]	*bgaA*'::*tet*-P_{Zn}-RBS^{ftsA}
AJP32	ACATCGCTTCCTCTCTATCTTCCTTGTTATAA TAGATTTATGAACACCTTGTTCATTATC		
TT394	GGAAGATAGAGAGGAAGCGATGTAATGACA TTTTCATTTGATACAGCTGCTG	IU7667	RBS^{ftsA}-*ftsZ*-Myc
AJP34	AACTGGTTTATGAGAAAGTAAGTTCTTTTAA AGATCTTCTTCAGAAATAAGTTTTTGTTC		
AJP35	AAAAACTTATTTCTGAAGAAGATCTTTAAAA GAACTTACTTTCTCATAAACCAGTTGCTG	D39	3' fragment containing *bgaA'*
CS121	GCTTTCTTGAGGCAATTCACTTGGTGC		
**For construction of IU8793 (*bgaA*'::*tet*-P_{Zn}-RBS^{ftsA}-*ezrA*-L_0-FLAG^{3})**			
TT657	CGCCCCAAGTTCATCACCAATGACATCAAC	IU3966[e]	*bgaA*'::*tet*-P_{Zn}-RBS^{ftsA}
AJP32	ACATCGCTTCCTCTCTATCTTCCTTGTTATAA TAGATTTATGAACACCTTGTTCATTATC		
AJP37	AAGGAAGATAGAGAGGAAGCGATGTAATGT CTAATGGACAACTAATTTATTTAATGGT	D39	RBS^{ftsA}-*ezrA*-L
AJP08	CGGAGCCAGCGGAACCAAAACGAATCGTTTC ACGTGTTTTCT		
AJP09	ACACGTGAAACGATTCGTTTTGGTTCCGCTG GCTCCGCT	IU4355	L-FLAG^{3} at *bgaA*
CS121	GCTTTCTTGAGGCAATTCACTTGGTGC		
**For construction of IU8795, IU8902 and IU8906 (*bgaA*'::*tet*-P_{Zn}-RBS^{ftsA}-*ezrA*$^{+}$)**			
TT657	CGCCCCAAGTTCATCACCAATGACATCAAC	IU3966[e]	*bgaA*'::*tet*-P_{Zn}-RBS^{ftsA}
AJP32	ACATCGCTTCCTCTCTATCTTCCTTGTTATAA TAGATTTATGAACACCTTGTTCATTATC		
AJP37	AAGGAAGATAGAGAGGAAGCGATGTAATGT CTAATGGACAACTAATTTATTTAATGGT	IU5795	RBS^{ftsA}-*ezrA*$^{+}$-*bgaA*
CS121	GCTTTCTTGAGGCAATTCACTTGGTGC		
**For construction of IU8845 (*ftsZ*-L_2-*gfp* markerless)**			
TT165	AGTGGTGCCGATATGGTCTTCATCACTGCT	D39	3' *ftsZ*
TT695	CATCTGCAGGAACTCGATGTCTAGTTTACGA TTTTTGAAAAATGGAGGTGTATCC		

TT693	AAACTAGACATCGAGTTCCTGCAGATGATTTCTAAAGGTGAAGAATTGTTACAGG	pUC57-*gfp*(*Sp*)[f]	L_2-*gfp*
TT694	TTACTTAACGATTTTTGAAAAATGTTATTTATACAATTCATCCATACCATGTGTAATACC		
TT696	CATGGTATGGATGAATTGTATAAATAACATTTTTCAAAAATCGTTAAGTAAATGAATGTA	D39	3’ downstream of *ftsZ*
TT166	TCATTGGGAGAGCCGGTTCCTGTGAAGAAT		
**For construction of IU9085 (Δ*mapZ*::P_c-*erm*)**			
P1523	GAGGTCTCTATTCTCAAAGATGTGGCAACTGTC	D39	Upstream of *mapZ* and 5’ 57 bp of *mapZ*
P1524	CATTATCCATTAAAAATCAAACGGATCCTAATCAAATTGCGGTTCTTGAGCTTCT		
Kan rpsL forward	TAGGATCCGTTTGATTTTTAATGGATAATG	P_c-*erm*[g]	P_c-*erm*
Kan rpsL reverse	GGGCCCCTTTCCTTATGCTTTTG		
P1525	TCCAAAAGCATAAGGAAAGGGGCCCTGTAAGACAGGCTACTTTGTCGGAAATGGC	D39	3’ 60 bp of *mapZ* and downstream
P1526	AATTGCATATCACCGTACTCAATACCATTGTG		
**For construction of IU10065 (*zapA*-L_4-*sfgfp*)**			
P1488	TGGAAGCTGATAACCCAGTTCTCGTCCCAGAT	D39	5’ fragment of *zapA*
TT812	AACAGCTCTTCTCCTTTTGTAGCAATAGAACGTAAGGAATCCTCAATCTTGCTCTGTTCT		
TT813	CAAGATTGAGGATTCCTTACGTTCTATTGCTACAAAAGGAGAAGAGCTGTTCACAGGTGT	IU9683	*sfgfp* middle fragment
TT799	TTATAAAGCTCATCCATGCCGTGAGTGATA		
TT815	TCACTCACGGCATGGATGAGCTTTATAAATGATTTCATTCCTTCTTCTATTGGTCTTGGT	D39	3’ fragment downstream of *zapA*
**For construction of IU10265 (*zapA*-L_4-FLAG)**			
P1488	TGGAAGCTGATAACCCAGTTCTCGTCCCAGAT	IU10065	5’ upstream of *zapA* including L_4-FLAG
TT840	AAATCATTTATCATCATCATCTTTATAATCTGTAGCAATAGAACGTAAGGAATCCTCAAT		
TT841	TGCTACAGATTATAAAGATGATGATGATAAATGATTTCATTCCTTCTTCTATTGGTCTTG	IU10065	L_4-FLAG + downstream of *zapA*
P1489	TTATCTGCTTTGGCAGTCGGAGCCAGTTGT		
**For construction of IU10267 (*zapA*-L_4-HA)**			
P1488	TGGAAGCTGATAACCCAGTTCTCGTCCCAGAT	IU10065	5’ upstream of *zapA* including L_4-HA
TT842	TTTAAGCATAATCTGGAACATCATATGGATATGTAGCAATAGAACGTAAGGAATCCTCAA		
TT843	TATCCATATGATGTTCCAGATTATGCTTAAATGATTTCATTCCTTCTTCTATTGGTCTTG	IU10065	3’ downstream of *zapA*

P1489	TTATCTGCTTTGGCAGTCGGAGCCAGTTGT		including L_4-HA
**For construction of IU10447 (*ezrA*-P_c-*erm*)**			
TT192	ATCGTGTTCCAGCCTTGGTTACGACGCTTT	D39	3’ *ezrA*$^+$
AJP134	AACAAATTTTGGGCCCGGTTAAAAACGAATCGTTTCACGTGTTTTCT		
AJP135	AACACGTGAAACGATTCGTTTTTAACCGGGCCCAAAATTTGTTTGATTT	IU6545	P_c-*erm* and downstream of *ezrA*
TT330	GAGGAGTTCGGACTCGACTCTCTCCTTCAAGAA		
**For construction of IU10901 (*ezrA*(QND)-P_c-*erm*)**			
AL295	CCCAAATCCACAGTTTGAAGGACAAACG	D39	5’ fragment with *ezrA*(R515D)
AJP142	GTTCATCAAATGAGCGATAATCGTTAGAATATTGCAAGAGTT		
AJP143	TCTTGCAATATTCTAACGATTATCGCTCATTTGATGAACGC	IU10447	*ezrA*(R515D)-P_c-*erm* and downstream
TT330	GAGGAGTTCGGACTCGACTCTCTCCTTCAAGAA		
**For construction of IU10909 (*ezrA*ΔQNR-P_c-*erm*)**			
AL295	CCCAAATCCACAGTTTGAAGGACAAACG	D39	5’ ezrA
AJP112	ATGCGTTCATCAAATGAGCGGAGTTGCTCTGTCAAAGTTGCATATTGTA		
AJP113	TATGCAACTTTGACAGAGCAACTCCGCTCATTTGATGAACGCATTCA	IU10447	*ezrA*ΔQNR-P_c-*erm* + downstream
TT330	GAGGAGTTCGGACTCGACTCTCTCCTTCAAGAA		
**For construction of IU11123 (*ezrA*ΔTM-P_c-*erm*)**			
AL295	CCCAAATCCACAGTTTGAAGGACAAACG	gDNA	Upstream *ezrA*
AJP204	CTCTAATCTCCCCTCGTTTCGCTTCATATCAAACTCCTTTTTTACTTGAAACAATCGTAA		
AJP205	ATTGTTTCAAGTAAAAAAGGAGTTTGATATGAAGCGAAACGAGGGGAGATTAGAGGCGCT	IU10447	*ezrA*ΔTM(Δ2-28 aa)-P_c-*erm* + downstream
TT330	GAGGAGTTCGGACTCGACTCTCTCCTTCAAGAA		
**For construction of IU12253 (*zapA*-L_4-*sfgfp*-P_c-*aad9*)**			
P1488	TGGAAGCTGATAACCCAGTTCTCGTCCCAGAT	IU10065	*zapA*-L_4-*sfgfp* fragment
TT934	ATCACATTATCCATTAAAAATCAAACGGATCCTATCATTTATAAAGCTCATCCATGCCGT		
Kan rpsL forward	TAGGATCCGTTTGATTTTTAATGGATAATG	P_c-*aad9* common cassette	Middle P_c-*aad9* fragment
Kan rpsL reverse	GGGCCCCTTTCCTTATGCTTTTG		
TT935	AAACGTCCAAAAGCATAAGGAAAGGGGCCCATGATTTCATTCCTTCTTCTATTGGTCTTG	IU10065	downstream of *zapA*
P1489	TTATCTGCTTTGGCAGTCGGAGCCAGTTGT		

**For construction of IU13123 (CEP::P_{Zn}-*ezrA*$^+$)**			
KW116	CCGGTAGTGGGAAAACAACTATTGGTCGTG C	IU7334	5’ fragment containing CEP::
JQ145	CCGTATCAGCAAAACCAAAAAAGCCATCTA GTAGAAACGCAAAAAGGCCATCCGTCAGGA		
JQ146	TCCTGACGGATGGCCTTTTTGCGTTTCTACT AGATGGCTTTTTGGTTTTGCTGATACGG	IU9805	P_{Zn}-RBS(*ftsA*)
AJP32	ACATCGCTTCCTCTCTATCTTCCTTGTTATAA TAGATTTATGAACACCTTGTTCATTATC		
AJP37	AAGGAAGATAGAGAGGAAGCGATGTAATGT CTAATGGACAACTAATTTATTTAATGGT	IU7334	RBS(*ftsA*)-*ezrA*$^+$-CEP’
KW123	GGCTTCTTGTTCAAATTTTCCCATTTGATTCT C		
**For construction of IU13189 (*ezrA*(QND)-L_0-*sfgfp*-P_c-*cat*)**			
AJP153	CCCAAATCCACAGTTTGAAGGACAAACG	IU10901	5’ fragment
TT193	CGGAGCCAGCGGAACCAAAACGAATCGTTT CACGTGTTTTC		
AL351	CGATTCGTTTTGGTTCCGCTGGCTCCGCTGC	IU11119	3’ fragment
TT330	GAGGAGTTCGGACTCGACTCTCTCCTTCAAG AA		
**For construction of IU13191 (*ezrA*(ΔQNR)-L_0-*sfgfp*-P_c-*cat*)**			
AJP153	CCCAAATCCACAGTTTGAAGGACAAACG	IU10909	5’ fragment with deletion of nt encoding aa510-516
TT193	CGGAGCCAGCGGAACCAAAACGAATCGTTT CACGTGTTTTC		
AL351	CGATTCGTTTTGGTTCCGCTGGCTCCGCTGC	IU11119	3’ fragment containing L_0-*sfgfp*-P_c-*cat*
TT330	GAGGAGTTCGGACTCGACTCTCTCCTTCAAG AA		
**For construction of IU13269 (*ezrA*(ΔTM)-L_0-*sfgfp*-P_c-*cat*)**			
AJP153	CCCAAATCCACAGTTTGAAGGACAAACG	IU11123	*ezrA*(ΔTM, aa 2-28)
TT193	CGGAGCCAGCGGAACCAAAACGAATCGTTT CACGTGTTTTC		
AL351	CGATTCGTTTTGGTTCCGCTGGCTCCGCTGC	IU11119	3’ fragment containing L_0-*sfgfp*-P_c-*cat*
TT330	GAGGAGTTCGGACTCGACTCTCTCCTTCAAG AA		
**For construction of IU13327 (*bgaA::kanT1T2*-P_{Zn}-*ezrA*$^+$)**			
TT657	CGCCCCAAGTTCATCACCAATGACATCAAC	IU9805	*bgaA::kanT1T2*-P_{Zn}-rbs*ftsA*
AJP32	ACATCGCTTCCTCTCTATCTTCCTTGTTATAA TAGATTTATGAACACCTTGTTCATTATC		
AJP37	AAGGAAGATAGAGAGGAAGCGATGTAATGT CTAATGGACAACTAATTTATTTAATGGT	IU8795	rbs*ftsA*-*ezrA*$^+$-*bgaA*
CS121	GCTTTCTTGAGGCAATTCACTTGGTGC		
**For construction of IU13822 (*zapJ*-L_0-*sfgfp*-P_c-*cat*)**			
AJP329	TGCCCAGTTACAACAGATGCGAGACCAT	D39	5’ *spd_1350*
AJP331	CGGAGCCAGCGGAACCTTCTGTCATTCTGGT CAGATTCAACTCT		

AJP332	TTGAATCTGACCAGAATGACAGAAGGTTCC GCTGGCTCCGCT	IU11121 *ezrA*-L$_0$-*sfGFP*-P$_c$-*cat*	L$_0$-*sfGFP*-P$_c$-*cat*
Kan rpsL rev	GGGCCCCTTTCCTTATGCTTTTG		
AJP333	GCATAAGGAAAGGGGCCCTAGGGGAGAAA ACATGTCAAAGACATATC	D39	3' downstream *spd_1350*
AJP330	GTCCACGGAAATGAACGGTGAAGGTTGAA		
**For construction of IU13922 (Δ*zapJ(spd_1350)*::P$_c$-[*kan-rpsL*$^+$]**			
AJP329	TGCCCAGTTACAACAGATGCGAGACCAT	D39	5' upstream *spd_1350* +60nt
AJP342	CCATTAAAAATCAAACGGATCCTATGGCATT TCAGTCAACATGACCTC		
Kan rpsL for	TAGGATCCGTTTGATTTTTAATGGATAATG	Pc-[*kan-rpsL*$^+$] cassette	Pc--[*kan-rpsL*$^+$]
Kan rpsL rev	GGGCCCCTTTCCTTATGCTTTTG		
AJP343	GCATAAGGAAAGGGGCCCCAAACAGAACAA GAACGTCGGGTT	D39	3' downstream *spd_1350*+60nt
AJP330	GTCCACGGAAATGAACGGTGAAGGTTGAA		
**For construction of IU13924 (Δ*zapJ(spd_1350)*::P$_c$-*erm*)**			
AJP329	TGCCCAGTTACAACAGATGCGAGACCAT	D39	5' upstream *spd_1350* +60 nt
AJP342	CCATTAAAAATCAAACGGATCCTATGGCATT TCAGTCAACATGACCTC		
Kan rpsL for	TAGGATCCGTTTGATTTTTAATGGATAATG	Pc-*erm* cassette	Pc-*erm* middle
Kan rpsL rev	GGGCCCCTTTCCTTATGCTTTTG		
AJP343	GCATAAGGAAAGGGGCCCCAAACAGAACAA GAACGTCGGGTT	D39	3' downstream *spd_1350*+60nt
AJP330	GTCCACGGAAATGAACGGTGAAGGTTGAA		
**For construction of IU15025 (*zapJ*-L$_0$-*ht*-P$_c$-*erm*)**			
AJP329	TGCCCAGTTACAACAGATGCGAGACCAT	D39	5' fragment
AJP331	CGGAGCCAGCGGAACCTTCTGTCATTCTGGT CAGATTCAACTCT		
AJP332	TTGAATCTGACCAGAATGACAGAAGGTTCC GCTGGCTCCGCT	IU14404	Middle containing L$_0$-*ht*-P$_c$-*erm*
AJP344	GTCTTTGACATGTTTTCTCCCCTATTTCCTCC CGTTAAATAATAGATAACTATTAAAAA		
AJP345	AGTTATCTATTATTTAACGGGAGGAAATAGG GGAGAAAACATGTCAAAGACATATC	D39	3' fragment
AJP330	GTCCACGGAAATGAACGGTGAAGGTTGAA		
**For construction of E42 (Δ*lytA*::P$_c$-*erm*)**			
P166	CCTTTGCCCTTCTTCCTATGACCGCTAT	D39	Upstream of *lytA* + 60 bp of *lytA*
P168	CATTATCCATTAAAAATCAAACGGATCCTAA TATGGTTGCACGCCGACTTGAGGC		
Kan rpsL forward	TAGGATCCGTTTGATTTTTAATGGATAATG	P$_c$-*erm* cassette[g]	P$_c$-*erm*

Kan rpsL reverse	GGGCCCCTTTCCTTATGCTTTTG		
P169	CAAAAGCATAAGGAAAGGGGCCCCTGGCAGACAGGCCAGAATTCACAGTAGAG	D39	60 bp of 3' *lytA* and downstream
P167	CCTCAACCATCCTATACAGTGAAGATGGGA		
**For construction of E733 (Δ*sepF(spd_1477)*::P$_c$-*erm*)**			
P1477	ACTACCGTGAGACAGTGAAACCAGCTCATTC	D39	Upstream of *sepF* + 60 bp of *sepF*
P1479	CATTATCCATTAAAAATCAAACGGATCCTATGAATCCTCATCCTCCGTAAAATAATCTAT		
Kan rpsL forward	TAGGATCCGTTTGATTTTTAATGGATAATG	P$_c$-*erm* cassette[g]	P$_c$-*erm*
Kan rpsL reverse	GGGCCCCTTTCCTTATGCTTTTG		
P1480	CAAAAGCATAAGGAAAGGGGCCCCAGATGAAGATCAACAGGGTGAGTT	D39	60 bp of 3' *sepF* and downstream
P1478	GTTCCTCCAGCGAAACAGGTATACGACCAA		
**For construction of E743 (Δ*zapA* (*spd_0369*)::P$_c$-*erm*)**			
P1488	TGGAAGCTGATAACCCAGTTCTCGTCCCAGAT	D39	Upstream of *zapA* + 5' 60 bp of *zapA*
P1490	CATTATCCATTAAAAATCAAACGGATCCTAGGTTAACGATTTTTCCCGAATGTAAA		
Kan rpsL forward	TAGGATCCGTTTGATTTTTAATGGATAATG	P$_c$-*erm* cassette[g]	P$_c$-*erm*
Kan rpsL reverse	GGGCCCCTTTCCTTATGCTTTTG		
P1491	CAAAAGCATAAGGAAAGGGGCCCCTTGTGACTTGTAAGCAAGAACAGAGCA	D39	3' 45 bp of *zapA* + downstream
P1489	TTATCTGCTTTGGCAGTCGGAGCCAGTTGT		
**For construction of E745 (Δ*spd_0370*::P$_c$-*erm*)**			
P1492	GTGAGAGAAGGAGTGCCTGGTGCTGGATTT	D39	Upstream of *spd_0370* + 5' 60 bp of *spd_0370*
P1494	CATTATCCATTAAAAATCAAACGGATCCTATCTCCGATAGCCGATATAAAATCCCC		
Kan rpsL forward	TAGGATCCGTTTGATTTTTAATGGATAATG	P$_c$-*erm* cassette[g]	P$_c$-*erm*
Kan rpsL reverse	GGGCCCCTTTCCTTATGCTTTTG		
P1495	CAAAAGCATAAGGAAAGGGGCCCAGCATACCGATAACAACCAGTTGGC	D39	3' 57 bp of *spd_0370* and downstream
P1493	TGCTCGCAGACTAGCAATTTCTTCGCTCAGTT		
**For construction of E747 Δ[*zapA(spd_0369)-spd_0370*]::P$_c$-*erm***			

P1488	TGGAAGCTGATAACCCAGTTCTCGTCCCAGAT	D39	Upstream of *zapA* + 5' 60 bp of *zapA*
P1490	CATTATCCATTAAAAATCAAACGGATCCTAGGTTAACGATTTTTCCCGAATGTAAA		
Kan rpsL forward	TAGGATCCGTTTGATTTTTAATGGATAATG	P_c-*erm* cassette[g]	P_c-*erm*
Kan rpsL reverse	GGGCCCCTTTCCTTATGCTTTTG		
P1495	CAAAAGCATAAGGAAAGGGGCCCAGCATACCGATAACAACCAGTTGGC	D39	3' 57 bp of *spd_0370* and downstream
P1493	TGCTCGCAGACTAGCAATTTCTTCGCTCAGTT		
**For construction of K743 (Δ*zapA* (*spd_0369*)::P_c-[*kan-rpsL*$^+$]**			
P1488	TGGAAGCTGATAACCCAGTTCTCGTCCCAGAT	D39	Upstream of *zapA* + 5' 60 bp of *zapA*
P1490	CATTATCCATTAAAAATCAAACGGATCCTAGGTTAACGATTTTTCCCGAATGTAAA		
Kan rpsL forward	TAGGATCCGTTTGATTTTTAATGGATAATG	P_c-*kan-rpsL*$^+$ *cassette*[g]	P_c-*kan-rpsL*$^+$
Kan rpsL reverse	GGGCCCCTTTCCTTATGCTTTTG		
P1491	CAAAAGCATAAGGAAAGGGGCCCCTTGTGACTTGTAAGCAAGAACAGAGCA	D39	3' 45 bp of *zapA* + downstream
P1489	TTATCTGCTTTGGCAGTCGGAGCCAGTTGT		
**For construction of K747 Δ[*zapA(spd_0369)-spd_0370*]::P_c-[*kan-rpsL*$^+$]**			
P1488	TGGAAGCTGATAACCCAGTTCTCGTCCCAGAT	D39	Upstream of *zapA* + 5' 60 bp of *zapA*
P1490	CATTATCCATTAAAAATCAAACGGATCCTAGGTTAACGATTTTTCCCGAATGTAAA		
Kan rpsL forward	TAGGATCCGTTTGATTTTTAATGGATAATG	P_c-*kan-rpsL+ cassette*[g]	P_c-*kan-rpsL*$^+$
Kan rpsL reverse	GGGCCCCTTTCCTTATGCTTTTG		
P1495	CAAAAGCATAAGGAAAGGGGCCCAGCATACCGATAACAACCAGTTGGC	D39	3' 57 bp of *spd_0370* and downstream
P1493	TGCTCGCAGACTAGCAATTTCTTCGCTCAGTT		

[a]Genomic DNA of indicated *S. pneumoniae* strains was used as templates for PCR reactions. Strain genotypes are listed in **Supplementary Table 1**, unless noted below.

[b]IU4888 (D39 *Δcps ΔgpsB*<>*aad9*//*bgaA*'::P_{fcsK}-*gpsB*$^+$) (Land *et al.,* 2013)

[c]IU6397 (D39 *rpsL1* Δ*phoU2 bgaA'*::*kan*-t1t2-P_{ftsA}-*phoU2*$^+$) (Zheng *et al.,* 2016)

[d]IU7426 (D39 *Δcps pbp2b*-HA^4-P_c-*kan*) (Tsui *et al.,* 2014)

[e]IU3966 (D39 *bgaA*'::*tet*-P_{Zn}-GFP-*divIVA*). Amplicon was templated from pJWV25 (Eberhardt *et al.,* 2009).

[f]pUC57-*gfp*(*Sp*) (Martin *et al.,* 2010)

[g]P_c-*erm* and P_c-*kan-rpsL*$^+$ cassettes are described in (Tsui *et al.,* 2011).

[h]Amplicons from IU7814 or IU5795 containing Δ*ftsZ*::aad9 or Δ*ezrA*<>aad9 were used for transformation experiments to test for essentiality. These alleles were amplified with the respective outside primers.

Supplementary Table 3. Percent live cells during EzrA depletion determined by Live/Dead staining

Strain and condition[a]		Percent live[b]	n[c]
D39 Δ*cps*	-Zn	96.0 ± 0.5%	188
D39 Δ*cps* Δ*ezrA*//P_{Zn}-*ezrA*$^+$	+Zn 2h	92.8 ± 6.2%	189
	-Zn 2h	89.6 ± 9.5%	261
	-Zn 3h	93.0 ± 0.4%	212
	-Zn 7h	96.7 ± 1.4%	210

[a]D39 Δ*cps* (IU1945), D39 Δ*cps* Δ*ezrA*//P_{Zn}-*ezrA*$^+$ (IU8799), were grown in the presence (+Zn), or absence of (-Zn) supplemented $ZnCl_2/MnSO_4$ for the indicated amount of time, as described *in Materials and Methods.* For viewing at 7 h time point, initial OD_{620} was ≈0.002. Live/Dead staining occurred as described in *Materials and Methods*.

[b]Percent survival is determined by total cells stained as "live" divided by "live+dead," averaged from two separate experiments ± SEM.

[c]n= number of cells analyzed. Data is from two biological replicates in which n is between 80-161 cells per replicate. Cells were analyzed from at least 4 separate fields per experiment. Cells which showed no labeling (>2%) were excluded from the analysis.

Supplementary Table 4. Percent anucleate cells determined by DAPI staining

Strain and condition[a]		Percent anucleate[b]	[c]n =
D39 Δ*cps*	-Zn	0	400
D39 Δ*cps* Δ*ezrA*//P_{Zn}-*ezrA*$^+$	+Zn 2hr	0.25 ± 0.25%	400
	-Zn 4hr	3.25 ± 0.75%	400
D39 Δ*cps* Δ*mapZ*::P_c-[*kan-rpsL*$^+$]	-Zn	0.5 ± 0%	400

[a]D39 Δ*cps* (IU1945), D39 Δ*cps* Δ*ezrA*//P_{Zn}-*ezrA*$^+$ (IU8799), D39 Δ*cps* Δ*mapZ*::P_c-[*kan-rpsL*$^+$] (IU9711), were grown in the presence (+Zn) or absence (-Zn) of 0.5 mM $ZnCl_2$ and 0.05 mM $MnSO_4$ for the indicated amount of time. Depletion and fixation for DAPI staining were performed as described in *Materials and Methods*.

[b]Percent anucleate was determined by the presence of DAPI labeling in the cell. ± indicated the SEM.

[c]n= number of cells analyzed. Data were obtained from biological replicates in which n is 200 pre-divisional cells or daughters of post-divisional cells per replicate. Cells were analyzed from at least 2 separate fields per experiment.

308 **Supplementary Table 5**. Antibody labeling conditions used for IFM in this study[a]

Strain No.	Proteins detected	Primary antibody			Secondary antibody		
		Antibody	Temp	Time	Antibody	Temp	Time
IU7223<br>IU8237<br>IU9713<br>IU9723	EzrA-HA<br>FtsZ-Myc	Rabbit anti-HA<br>Mouse anti-Myc	24°C	1 h	488 anti-Rabbit<br>568 anti-Mouse	24°C	1 h
IU8596	SepF-HA<br>FtsZ-Myc	Rabbit anti-HA<br>Mouse anti-Myc	24°C	1 h	488 anti-Rabbit<br>568 anti-Mouse	24°C	1 h
IU8681	EzrA-FLAG[3]<br>FtsZ-Myc	Rabbit anti-FLAG<br>Mouse anti-Myc	24°C	1 h	488 anti-Rabbit<br>568 anti-Mouse	24°C	1 h
IU1945<br>IU8799	FtsZ	Rabbit anti-FtsZ	37°C	1 h	488 anti-Rabbit	24°C	1 h
IU10752	ZapA-FLAG<br>FtsZ-Myc	Rabbit anti-FLAG<br>Mouse anti-Myc	24°C	1 h	488 anti-Rabbit<br>568 anti-Mouse	24°C	1 h

310

311 [a]IFM protocol is described in *Materials and Methods*.

312

Supplementary Table 6. Plasmids expressing *S. pneumoniae* proteins used in B2H assays in this study

**Name**	**Relevant characteristics**	**Two-hybrid construct**	**Source/ reference**
pKT25_*ftsA* (pMKV24)	*kan* P_{lac}-*cya(T25)-ftsA*	FtsA-T25	Krupka *et al.,* 2012
pUT18C_*ftsA* (pMKV19)	*amp* P_{lac}-*cya(T18)-ftsA*	FtsA-T18	Krupka *et al.,* 2012
pKNT25_*ftsZ*	*kan* P_{lac}-*ftsZ-cya(T25)*	FtsZ-T25	Rued *et al.,* 2017
pUT18_*ftsZ*	*amp* P_{lac}-*ftsZ-cya(T18)*	FtsZ-T18	Rued *et al.,* 2017
pKNT25_*ezrA*	*kan* P_{lac}-*ezrA-cya(T25)*	EzrA-T25	Rued *et al.,* 2017
pUT18_*ezrA*	*amp* P_{lac}-*ezrA -cya(T18)*	EzrA-T18	Rued *et al.,* 2017
pKNT25_*stkP*	*kan* P_{lac}-*stkP-cya(T25)*	StkP-T25	Rued *et al.,* 2017
pUT18_*stkP*	*amp* P_{lac}-*stkP-cya(T18)*	StkP-T18	Rued *et al.,* 2017
pKNT25_*divIVA*	*kan* P_{lac}-*divIVA-cya(T25)*	DivIVA-T25	Rued *et al.,* 2017
pUT18_*divIVA*	*amp* P_{lac}-*divIVA-cya(T18)*	DivIVA-T18	Rued *et al.,* 2017
pKNT25_*gpsB*	*kan* P_{lac}-*gpsB-cya(T25)*	GpsB-T25	Rued *et al.,* 2017
pUT18_*gpsB*	*amp* P_{lac}-*gpsB-cya(T18)*	GpsB-T18	Rued *et al.,* 2017
pFC113	*kan* P_{lac}-*cya(T25)-mreC*	T25-MreC	Cleverley *et al.,* 2019
pFC114	*amp* P_{lac}-*cya(T18)-mreC*	T18-MreC	Cleverley *et al.,* 2019
pFC115	*kan* P_{lac}-*cya(T25)-pbp2a*	T25-PBP2a	Cleverley *et al.,* 2019
pFC116	*amp* P_{lac}-*cya(T18)-pbp2a*	T18-PBP2a	Cleverley *et al.,* 2019
pFC123	*kan* P_{lac}-*cya(T25)-pbp1a*	T25-PBP1a	Cleverley *et al.,* 2019
pFC124	*amp* P_{lac}-*cya(T18)-pbp1a*	T18-PBP1a	Cleverley *et al.,* 2019
pFC125	*kan* P_{lac}-*cya(T25)-pbp2b*	T25-PBP2b	Cleverley *et al.,* 2019
pFC126	*amp* P_{lac}-*cya(T18)-pbp2b*	T18-PBP2b	Cleverley *et al.,* 2019
pFC127	*kan* P_{lac}-*cya(T25)-pbp2x*	T25-PBP2x	Cleverley *et al.,* 2019
pFC128	*amp* P_{lac}-*cya(T18)-pbp2x*	T18-PBP2x	Cleverley *et al.,* 2019
pFC141	*kan* P_{lac}-*cya(T25)-rodZ*	T25-RodZ	This work
pFC142	*amp* P_{lac}-*cya(T18)-rodZ*	T18-RodZ	This work

**Name**	**Relevant characteristics**	**Two-hybrid construct**	**Source/ reference**
pMBM147	*kan* P_{lac}-*cya(T25)-mpgA*	T25-MpgA (formerly MltG(*Spn*))	This work
pMBM148	*amp* P_{lac}-*cya(T18)-mpgA*	T18-MpgA	This work
pMBM149	*kan* P_{lac}-*cya(T25)-sepF*	T25-SepF	This work
pMBM150	*amp* P_{lac}-*cya(T18)-sepF*	T18-SepF	This work
pMBM151	*kan* P_{lac}-*cya(T25)-rodA*	T25-RodA	This work
pMBM152	*amp* P_{lac}-*cya(T18)-rodA*	T18-RodA	This work
pMBM153	*kan* P_{lac}-*cya(T25)-ftsW*	T25-FtsW	This work
pMBM154	*amp* P_{lac}-*cya(T18)-ftsW*	T18-FtsW	This work
pMBM155	*kan* P_{lac}-*cya(T25)-ftsL*	T25-FtsL	This work
pMBM156	*amp* P_{lac}-*cya(T18)-ftsL*	T18-FtsL	This work
pMBM157	*kan* P_{lac}-*cya(T25)-ftsQ/divIB*	T25-FtsQ	This work
pMBM158	*amp* P_{lac}-*cya(T18)-ftsQ-divIB*	T18-FtsQ	This work
pMBM159	*kan* P_{lac}-*cya(T25)-ftsB/divIC*	T25-FtsB	This work
pMBM160	*amp* P_{lac}-*cya(T18)-ftsB-divIC*	T18-FtsB	This work
pBKM161	*kan* P_{lac}-*cya(T25)-macP*	T25-MacP	B. Kupeska unpublished
pBKM162	*amp* P_{lac}-*cya(T18)-macP*	T18-MacP	B. Kupeska unpublished
pDDM169	*kan* P_{lac}-*mreD-cya(T25)*	MreD-T25	This work
pDDM170	*amp* P_{lac}-*mreD-cya(T18)*	MreD-T18	This work
pAZM183	*kan* P_{lac}-*cya(T25)-zapA*	T25-ZapA	This work
pAZM184	*kan* P_{lac}-*cya(T18)-zapA*	T18-ZapA	This work
pAZM185	*kan* P_{lac}-*cya(T25)-zapJ*	T25-ZapJ	This work
pAZM186	*kan* P_{lac}-*cya(T18)-zapJ*	T18-ZapJ	This work

**Name**	**Relevant characteristics**	**Two-hybrid construct**	**Source/ reference**
pAZM187	*kan* P_{lac}-*cya(T25)-ftsK*	T25-FtsK	This work
pAZM188	*kan* P_{lac}-*cya(T18)-ftsK*	T18-FtsK	This work
pKNT25_*mapZ/locZ*	*kan* P_{lac}-*mapZ-cya(T25)*	MapZ-T25	K. Buriánková unpublished
pUT18_*mapZ/locZ*	*amp* P_{lac}-*mapZ-cya(T18)*	MapZ-T18	K. Buriánková unpublished

Supplementary Table 7. Oligonucleotide primers used to construct and verify plasmids used for B2H assays in this study

**Primers used for cloning into B2H assay plasmids**		
Primer name	Sequence (5→3')	Template[a]
**Construction of T25/T18-fusions to *S. pneumoniae rodZ***		
pKT25/pUT18C_*rodZ*_BF	CGGGATCCTATGAGAAAAAAACAATTGGAGAGG	D39
pKT25/pUT18C_*rodZ*_ER	CGGAATTCTTAATTTTAGTAAAGGTTACAGTGA	
**Construction of T25/T18-fusions to *S. pneumoniae mpgA***		
pKT25/pUT18C_*mpgA*_XF	GCTCTAGAGATGAGTGAAAAGTCAAGAGAAGAAGAG	D39
pKT25/pUT18C_*mpgA*_BR	CGGGATCCTTAGTTTAATTTGCTGTTGACATGT	
**Construction of T25/T18-fusions to *S. pneumoniae sepF***		
pKT25/pUT18C_*sepF*_XF	GCTCTAGAGATGTCTTTAAAAGATAGATTCGATAG	D39
pKT25/pUT18C_*sepF*_BR	CGGGATCCTTATCGTACTCTATTTCGCTTCAT	
**Construction of T25/T18-fusions to *S. pneumoniae rodA***		
pKT25/pUT18C_*rodA*_BF	GCGGATCCCATGAAACGTTCTCTCGACTCTAGA	D39
pKT25/pUT18C_*rodA*_ER	CGGAATTCTTATTTAATTTGTTTTAATACAACCTTTTTC	
**Construction of T25/T18-fusion to *S. pneumoniae ftsW***		
pKT25/pUT18C_*ftsW*_XF	GCTCTAGAGATGAAGATTAGTAAGAGGCACTTAT	D39
pKT25/pUT18C_*ftsW*_BR	CGGGATCCCTACTTCAACAGAAGGTTCATTG	
**Construction of T25/T18-fusion to *S. pneumoniae ftsQ***		
pKT25/pUT18C_*ftsQ/divIB*_XF	GCTCTAGAGATGTCAAAAGATAAGAAAAATGAGG	D39
pKT25/pUT18C_*ftsQ/divIB*_BR	CGGGATCCCTAGCGACGCGATGAACGCT	

**Construction of T25/T18-fusion to *S. pneumoniae ftsL***		
pKT25/pUT18C_*ftsL*_XF	GCTCTAGAGATGGCAGAAAAAATG GAAAAAACA	D39
pKT25/pUT18C_*ftsL*_BR	CGGGATCCTTACTCCGCTATTCTAA TATTTTCA	
**Construction of T25/T18-fusion to *S. pneumoniae ftsB***		
pKT25/pUT18C_*ftsB/divIC*_XF	GCTCTAGAGATGTCTAAAAATATTG TACAATTGAAT	D39
pKT25/pUT18C_*ftsB/divIC*_BR	CGGGATCCTCACCTTTGAAGCAAGT CAGGA	
**Construction of T25/T18-fusion to *S. pneumoniae macP***		
pKT25/pUT18C_*macP*_XF	CGTCTAGAGATGGGTAAATCTTTAT TAACGGATG	D39
pKT25/pUT18C_*macP*_ER	GCGAATTCTTACAAAGTTTCATTG CTAAAACAAGC	
**Construction of fusion-T25/T18 to *S. pneumoniae mreD***		
pKNT25/pUT18_*mreD*_PF	AACTGCAGGATGAGACAGTTGAAG CGAGTTG	D39
pKNT25/pUT18_*mreD*_BR	CGGGATCCTCTAGATAATATTTTTC AAAAATAAATTGA	
**Construction of fusion-T25/T18 to *S. pneumoniae zapA***		
pKT25/pUT18C_*zapA*_XF	GCTCTAGAGATGGCAAATCTAAATC GATTCAAATTTACATTCG	D39
pKT25/pUT18C_*zapA*_BR	CGGGATCCTCATAAGGAATCCTCAA TCTTGCTCTGTTCTT	
**Construction of fusion-T25/T18 to *S. pneumoniae zapJ***		
pKT25/pUT18C_*zapJ*_XF	GCTCTAGAGATGAAACAAGAACGA TTTCCATTGGTGTCAG	D39
pKT25/pUT18C_*zapJ*_BR	CGGGATCCCTATTCTGTCATTCTGGT CAGATTCAACTC	
**Construction of fusion-T25/T18 to *S. pneumoniae ftsK***		
pKT25/pUT18C_*ftsK*_XF	GCTCTAGAGATGTTGATTTCGTTAG GAATTGCG	D39
pKT25/pUT18C_*ftsK*_BR	CGGGATCCTTATTGTTGTAACACTTT TCGAGG	

**Primers used for verification and sequencing (5'→3')**	
pKT25_579F	GTTCGCCATTATGCCGCATC
pKT25_802R	GGATGTGCTGCAAGGCGATT
pUT18C_484F	GATGTACTGGAAACGGTGC
pUT18C_660R	CTTAACTATGCGGCATCAGAGC
pKNT25/pUT18_49F	CGCAATTAATGTGAGTTAGC
pKNT25_328R	TTGATGCCATCGAGTACG
pUT18_304R	CGAGCGATTTTCCACAACAA
*mpgA*_794F	CCGACTTGAAAGCAGGTTAC
*mpgA*_813R	GTAACCTGCTTTCAAGTCGG
*ftsW*_596F	GGTTTTCAACCATTCTGGCG
*ftsW*_615R	CGCCAGAATGGTTGAAAACC
*rodA*_603F	GACTGCTGTAACAGGAGTTG
*rodA*_622R	CAACTCCTGTTACAGCAGTC
*ftsQ*_585F	GCAGATTAAGTCTAACTATTGG
*ftsQ*_606R	CCAATAGTTAGACTTAATCTGC
*ftsK*_1139F	TATCTTTCCGAGAACTATGG
*ftsK*_1158R	CCATAGTTCTCGGAAAGATA

3. SUPPLEMENTARY REFERENCES

Beilharz, K., Novakova, L., Fadda, D., Branny, P., Massidda, O., & Veening, J. W. (2012). Control of cell division in *Streptococcus pneumoniae* by the conserved Ser/Thr protein kinase StkP. *Proc Natl Acad Sci USA*, *109*(15), E905-913. https://doi.org/10.1073/pnas.1119172109

Boersma, M. J., Kuru, E., Rittichier, J. T., VanNieuwenhze, M. S., Brun, Y. V., & Winkler, M. E. (2015). Minimal Peptidoglycan (PG) Turnover in Wild-Type and PG Hydrolase and Cell Division Mutants of *Streptococcus pneumoniae* D39 Growing Planktonically and in Host-Relevant Biofilms. *J Bacteriol*, *197*(21), 3472-3485. https://doi.org/10.1128/jb.00541-15

Cleverley, R. M., Barrett, J. R., Baslé, A., Bui, N. K., Hewitt, L., Solovyova, A., Xu, Z. Q., Daniel, R. A., Dixon, N. E., Harry, E. J., Oakley, A. J., Vollmer, W., & Lewis, R. J. (2014). Structure and function of a spectrin-like regulator of bacterial cytokinesis. *Nat Comm*,*5*, 5421. https://doi.org/10.1038/ncomms6421

Cleverley, R. M., Rutter, Z. J., Rismondo, J., Corona, F., Tsui, H. T., Alatawi, F. A., Daniel, R. A., Halbedel, S., Massidda, O., Winkler, M. E., & Lewis, R. J. (2019). The cell cycle regulator GpsB functions as cytosolic adaptor for multiple cell wall enzymes. *Nat Comm*, *10*(1), 261. https://doi.org/10.1038/s41467-018-08056-2

Dam, P., Olman, V., Harris, K., Su, Z., & Xu, Y. (2007). Operon prediction using both genome-specific and general genomic information. *Nucleic Acids Res*, *35*(1), 288-298. https://doi.org/10.1093/nar/gkl1018

Eberhardt, A., Wu, L. J., Errington, J., Vollmer, W., & Veening, J. W. (2009). Cellular localization of choline-utilization proteins in *Streptococcus pneumoniae* using novel fluorescent reporter systems. *Mol Microbiol*, *74*(2), 395-408. https://doi.org/10.1111/j.1365-2958.2009.06872.x

Grimm, J. B., English, B. P., Chen, J., Slaughter, J. P., Zhang, Z., Revyakin, A., Patel, R., Macklin, J. J., Normanno, D., Singer, R. H., Lionnet, T., & Lavis, L. D. (2015). A general method to improve fluorophores for live-cell and single-molecule microscopy. *Nat Meth*, *12*(3), 244–250.

Haeusser, D. P., Garza, A. C., Buscher, A. Z., & Levin, P. A. (2007). The division inhibitor EzrA contains a seven-residue patch required for maintaining the dynamic nature of the medial FtsZ ring. *J Bacteriol*, *189*(24), 9001-9010. https://doi.org/10.1128/jb.01172-07

Karp, P. D., Billington, R., Caspi, R., Fulcher, C. A., Latendresse, M., Kothari, A., Keseler, I. M., Krummenacker, M., Midford, P. E., Ong, Q., Ong, W. K., Paley, S. M., & Subhraveti, P. (2019). The BioCyc collection of microbial genomes and metabolic pathways. *Brief bioinform*, *20*(4), 1085–1093. https://doi.org/10.1093/bib/bbx085

Karimova, G., Dautin, N., & Ladant, D. (2005). Interaction network among *Escherichia coli* membrane proteins involved in cell division as revealed by bacterial two-hybrid analysis. *J Bacteriol*, *187*(7), 2233–2243. https://doi.org/10.1128/JB.187.7.2233-2243.2005

Kelley, L. A., Mezulis, S., Yates, C. M., Wass, M. N., & Sternberg, M. J. E. (2015). The Phyre2 web portal for protein modeling, prediction and analysis [Protocol]. *Nat. Protocols*, *10*(6), 845-858. https://doi.org/10.1038/nprot.2015.053

Kjos, M., Aprianto, R., Fernandes, V. E., Andrew, P. W., van Strijp, J. A., Nijland, R., & Veening, J. W. (2015). Bright fluorescent *Streptococcus pneumoniae* for live-cell imaging of host-pathogen interactions. *J Bacteriol*, *197*(5), 807-818. https://doi.org/10.1128/jb.02221-14

Krupka, M., Rivas, G., Rico, A. I., & Vicente, M. (2012). Key role of two terminal domains in the bidirectional polymerization of FtsA protein. *J Biol Chem* , *287*(10), 7756–7765. https://doi.org/10.1074/jbc.M111.311563

Land, A. D., & Winkler, M. E. (2011). The requirement for pneumococcal MreC and MreD is relieved by inactivation of the gene encoding PBP1a. *J Bacteriol*, *193*(16), 4166-4179. https://doi.org/10.1128/jb.05245-11

Land, A. D., Luo, Q., & Levin, P. A. (2014). Functional domain analysis of the cell division inhibitor EzrA. *PLoS One*, *9*(7), e102616. https://doi.org/10.1371/journal.pone.0102616

Land, A. D., Tsui, H. C., Kocaoglu, O., Vella, S. A., Shaw, S. L., Keen, S. K., Sham, L. T., Carlson, E. E., & Winkler, M. E. (2013). Requirement of essential Pbp2x and GpsB for septal ring closure in *Streptococcus pneumoniae* D39. *Mol Microbiol*, *90*(5), 939-955. https://doi.org/10.1111/mmi.12408

Lanie, J. A., Ng, W. L., Kazmierczak, K. M., Andrzejewski, T. M., Davidsen, T. M., Wayne, K. J., Tettelin, H., Glass, J. I., & Winkler, M. E. (2007). Genome sequence of Avery's virulent serotype 2 strain D39 of *Streptococcus pneumoniae* and comparison with that of unencapsulated laboratory strain R6. *J Bacteriol*, *189*(1), 38-51. https://doi.org/10.1128/jb.01148-06

Lara, B., Rico, A. I., Petruzzelli, S., Santona, A., Dumas, J., Biton, J., Vicente, M., Mingorance, J., & Massidda, O. (2005). Cell division in cocci: localization and properties of the *Streptococcus pneumoniae* FtsA protein. *Mol Microbiol*, *55*(3), 699-711. https://doi.org/10.1111/j.1365-2958.2004.04432.x

Letunic, I., Doerks, T., & Bork, P. (2015). SMART: recent updates, new developments and status in 2015. *Nucleic Acids Res*, *43*(Database issue), D257-260. https://doi.org/10.1093/nar/gku949

Mao, F., Dam, P., Chou, J., Olman, V., & Xu, Y. (2009). DOOR: a database for prokaryotic operons. *Nucleic Acids Res*, *37*(Database issue), D459-463. https://doi.org/10.1093/nar/gkn757

Mao, X., Ma, Q., Zhou, C., Chen, X., Zhang, H., Yang, J., Mao, F., Lai, W., & Xu, Y. (2014). DOOR 2.0: presenting operons and their functions through dynamic and integrated views. *Nucleic Acids Res*, *42*(Database issue), D654-659. https://doi.org/10.1093/nar/gkt1048

Martin, B., Granadel, C., Campo, N., Henard, V., Prudhomme, M., & Claverys, J. P. (2010). Expression and maintenance of ComD-ComE, the two-component signal-transduction system that controls competence of *Streptococcus pneumoniae*. *Mol Microbiol*, *75*(6), 1513-1528. https://doi.org/10.1111/j.1365-2958.2010.07071.x

Mura, A., Fadda, D., Perez, A. J., Danforth, M. L., Musu, D., Rico, A. I., Krupka, M., Denapaite, D., Tsui, H. T., Winkler, M. E., Branny, P., Vicente, M., Margolin, W., & Massidda, O. (2016). Roles of the essential protein FtsA in cell growth and division in *Streptococcus pneumoniae*. *J Bacteriol*. https://doi.org/10.1128/jb.00608-16

Pazos, M., Natale, P., Margolin, W., & Vicente, M. (2013). Interactions among the early Escherichia coli divisome proteins revealed by bimolecular fluorescence complementation. *Environ Microbiol*, *15*(12), 3282-3291. https://doi.org/10.1111/1462-2920.12225

Perez, A. J., Boersma, M. J., Bruce, K. E., Lamanna, M. M., Shaw, S. L., Tsui, H. T., Taguchi, A., Carlson, E. E., VanNieuwenhze, M. S., & Winkler, M. E. (2021). Organization of peptidoglycan synthesis in nodes and separate rings at different stages of cell division of *Streptococcus pneumoniae*. *Mol Microbiol*, *115*(6),1152-1169. https://doi: 10.1111/mmi.14659

Perez, A. J., Cesbron, Y., Shaw, S. L., Bazan Villicana, J., Tsui, H. T., Boersma, M. J., Ye, Z. A., Tovpeko, Y., Dekker, C., Holden, S., & Winkler, M. E. (2019). Movement dynamics of divisome proteins and PBP2x:FtsW in cells of Streptococcus pneumoniae. *Proc Natl Acad Sci U S A*, *116*(8), 3211-3220. https://doi.org/10.1073/pnas.1816018116

Ponting, C. P., Schultz, J., Milpetz, F., & Bork, P. (1999). SMART: identification and annotation of domains from signalling and extracellular protein sequences. *Nucleic Acids Res*, *27*(1), 229-232.

Ramos-Montañez, S., Kazmierczak, K. M., Hentchel, K. L., & Winkler, M. E. (2010). Instability of *ackA* (acetate kinase) mutations and their effects on acetyl phosphate and ATP amounts in

Streptococcus pneumoniae D39. *J Bacteriol*, *192*(24), 6390–6400. https://doi.org/10.1128/JB.00995-10

Rued, B. E., Zheng, J. J., Mura, A., Tsui, H. T., Boersma, M. J., Mazny, J. L., Corona, F., Perez, A. J., Fadda, D., Doubravova, L., Buriankova, K., Branny, P., Massidda, O., & Winkler, M. E. (2017). Suppression and Synthetic-Lethal Genetic Relationships of Δ*gpsB* Mutations Indicate That GpsB Mediates Protein Phosphorylation and Penicillin-Binding Protein Interactions in *Streptococcus pneumoniae* D39. *Mol Microbiol*. https://doi.org/10.1111/mmi.13613

Richards, V. P., Palmer, S. R., Pavinski Bitar, P. D., Qin, X., Weinstock, G. M., Highlander, S. K., Town, C. D., Burne, R. A., & Stanhope, M. J. (2014). Phylogenomics and the dynamic genome evolution of the genus *Streptococcus*. *Gen Biol Evol*, *6*(4), 741–753.

Sham, L. T., Barendt, S. M., Kopecky, K. E., & Winkler, M. E. (2011). Essential PcsB putative peptidoglycan hydrolase interacts with the essential FtsXSpn cell division protein in *Streptococcus pneumoniae* D39. *Proc Natl Acad Sci USA*, *108*(45), E1061–E1069. https://doi.org/10.1073/pnas.1108323108

Sham, L. T., Jensen, K. R., Bruce, K. E., & Winkler, M. E. (2013). Involvement of FtsE ATPase and FtsX extracellular loops 1 and 2 in FtsEX-PcsB complex function in cell division of *Streptococcus pneumoniae* D39. *mBio*, *4*(4). https://doi.org/10.1128/mBio.00431-13

Sievers, F., Wilm, A., Dineen, D., Gibson, T. J., Karplus, K., Li, W., Lopez, R., McWilliam, H., Remmert, M., Söding, J., Thompson, J. D., & Higgins, D. G. (2011). Fast, scalable generation of high-quality protein multiple sequence alignments using Clustal Omega. *Mol Syst Biol*, *7*, 539.

Schultz, J., Milpetz, F., Bork, P., & Ponting, C. P. (1998). SMART, a simple modular architecture research tool: identification of signaling domains. *Proc Natl Acad Sci U S A*, *95*(11), 5857-5864.

Tsui, H. C., Boersma, M. J., Vella, S. A., Kocaoglu, O., Kuru, E., Peceny, J. K., Carlson, E. E., VanNieuwenhze, M. S., Brun, Y. V., Shaw, S. L., & Winkler, M. E. (2014). Pbp2x localizes separately from Pbp2b and other peptidoglycan synthesis proteins during later stages of cell division of *Streptococcus pneumoniae* D39. *Mol Microbiol*, *94*(1), 21-40. https://doi.org/10.1111/mmi.12745

Tsui, H. C., Keen, S. K., Sham, L. T., Wayne, K. J., & Winkler, M. E. (2011). Dynamic distribution of the SecA and SecY translocase subunits and septal localization of the HtrA surface chaperone/protease during *Streptococcus pneumoniae* D39 cell division. *mBio*, *2*(5), e00202-11.

Tsui, H. C., Mukherjee, D., Ray, V. A., Sham, L. T., Feig, A. L., & Winkler, M. E. (2010). Identification and characterization of noncoding small RNAs in *Streptococcus pneumoniae* serotype 2 strain D39. *J Bacteriol*, *192*(1), 264-279. https://doi.org/10.1128/jb.01204-09

Tsui, H. T., Zheng, J. J., Magallon, A. N., Ryan, J. D., Yunck, R., Rued, B. E., Bernhardt, T. G., & Winkler, M. E. (2016). Suppression of a Deletion Mutation in the Gene Encoding Essential PBP2b Reveals a New Lytic Transglycosylase Involved in Peripheral Peptidoglycan Synthesis in *Streptococcus pneumoniae* D39. *Mol Microbiol*. https://doi.org/10.1111/mmi.13366

van Raaphorst, R., Kjos, M., & Veening, J. W. (2017). Chromosome segregation drives division site selection in *Streptococcus pneumoniae*. *Proc Natl Acad Sci U S A*, *114*(29), E5959-E5968. https://doi.org/10.1073/pnas.1620608114

Wayne, K. J., Li, S., Kazmierczak, K. M., Tsui, H. C., & Winkler, M. E. (2012). Involvement of WalK (VicK) phosphatase activity in setting WalR (VicR) response regulator phosphorylation level and limiting cross-talk in *Streptococcus pneumoniae* D39 cells. *Mol Microbiol*, *86*(3), 645-660. https://doi.org/10.1111/mmi.12006

Wayne, K. J., Sham, L. T., Tsui, H. C., Gutu, A. D., Barendt, S. M., Keen, S. K., & Winkler, M. E. (2010). Localization and cellular amounts of the WalRKJ (VicRKX) two-component regulatory system proteins in serotype 2 *Streptococcus pneumoniae*. *J Bacteriol*, *192*(17), 4388-4394. https://doi.org/10.1128/jb.00578-10

Zheng, J. J., Sinha, D., Wayne, K. J., & Winkler, M. E. (2016). Physiological Roles of the Dual Phosphate Transporter Systems in Low and High Phosphate Conditions and in Capsule Maintenance of *Streptococcus pneumoniae* D39. *Front Cell Infect Microbiol*, *6*, 63. https://doi.org/10.3389/fcimb.2016.00063

4. SUPPLEMENTARY FIGURES AND LEGENDS

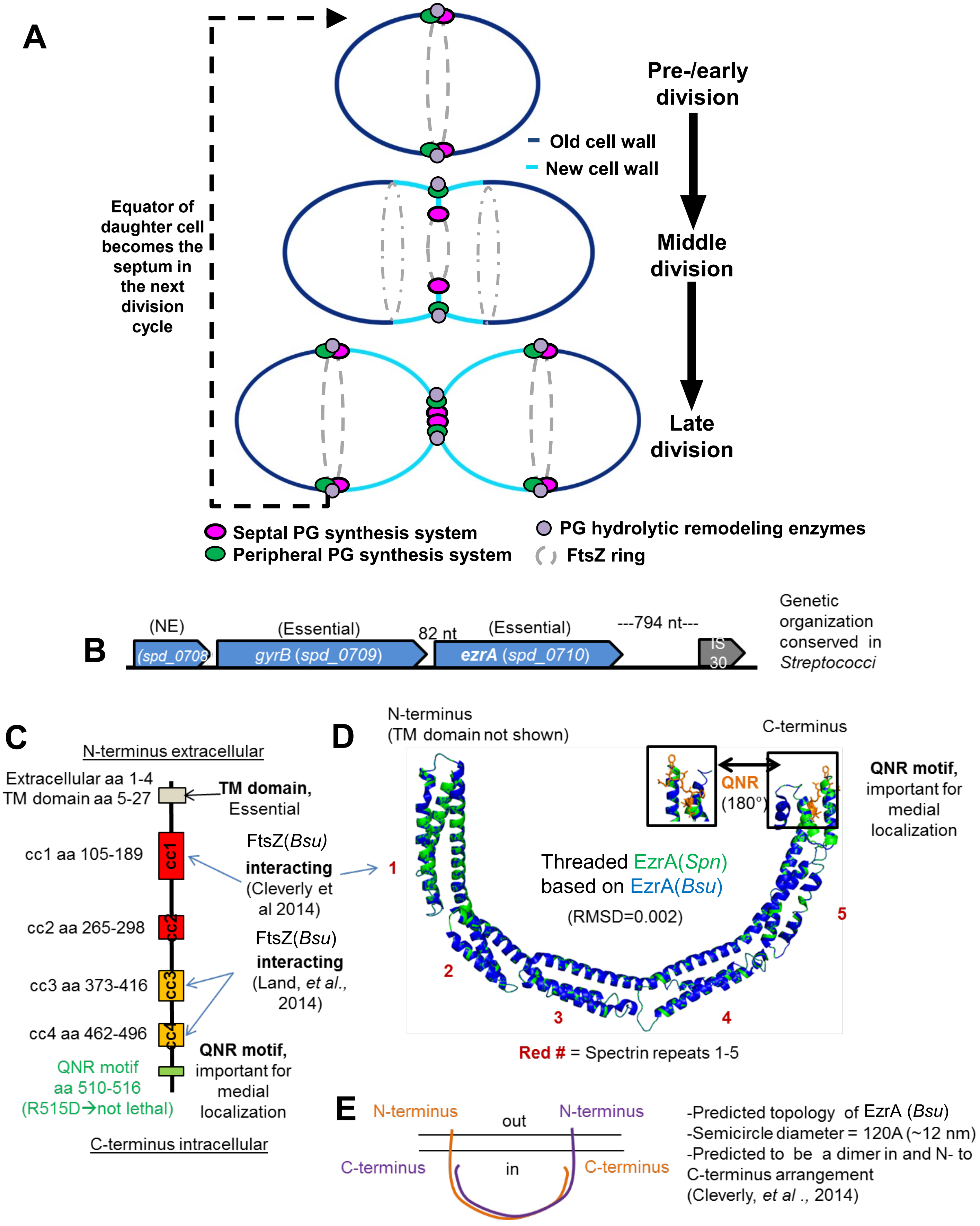

Supplementary Figure 1

Supplementary Figure 1. Schematic summary of cell division and peptidoglycan (PG) synthesis in *S. pneumoniae* (*Spn*) and genetic arrangement, protein topology, and 3D-overlayed structure with EzrA(*Bsu*) for EzrA(*Spn*) (A) Schematic drawing of the cell cycle of *S. pneumoniae* focusing on PG synthesis and FtsZ-ring localization throughout a cell cycle. (B) Schematic diagram of genes surrounding *ezrA* in the *S. pneumoniae* D39 chromosome. The genes encode the following proteins: *spd_0708* (uncharacterized putative protein); *spd_0709, gyrB* (DNA gyrase subunit-B); *spd_0710* (*ezrA*), and *spd_0711* (uncharacterized putative protein IS30 element). Genes of same color are predicted to be in the same operon using DOOR analysis (Dam *et al.,* 2007; Mao *et al.,* 2009; Mao *et al.,* 2014). Genetic arrangement of *spd_0708-0710*, but not necessarily the predicted operons, are conserved in all streptococci species tested (*S. pyogenes* M1 GAS, *S. thermophilus* LMG 18311, *S. parasanguinis* FW213). (C) 2D-analysis of EzrA(*Spn*) protein secondary structure using SMART tool (Letunic *et al.,* 2015; Ponting *et al.,* 1999; Schultz *et al.,* 1998). EzrA(*Spn*) is predicted to have 4 coiled-coiled regions as well as the designated QNR motif at the C-terminus. (D) EzrA(*Spn*) 3D-structure lacking the first 29 (transmembrane) residues was predicted using phyre^2 software then threaded onto the known crystal structure of EzrA(*Bsu*) (Cleverley *et al.,* 2014). EzrA(*Bsu*) is predicted to have 5 spectrin repeats, whose location is surrounding the red numbers (Spectrin repeats 1-5). The essential QNR motif (amino acids 510-516) is shown at the top right corner in orange, then rotated 180° in the box to the left. (E) Model for the intracellular organization of EzrA dimers in *B. subtilis*.

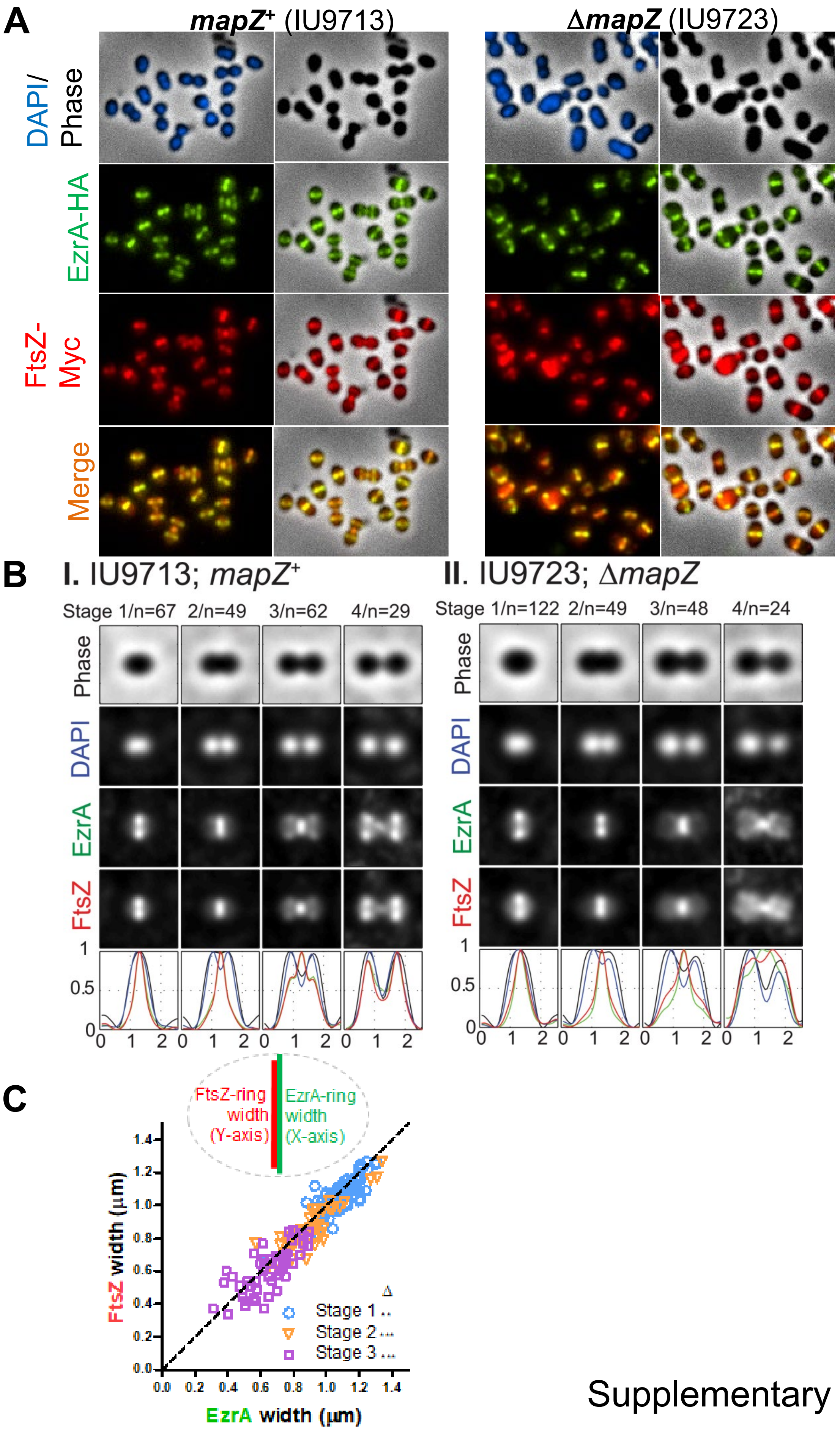

Supplementary Figure 2

Supplementary Figure 2. Co-localization of FtsZ- and EzrA-rings in wild-type or Δ*mapZ Spn* mutants. (A) 2D Representative phase and fluorescence images of strain IU9713 (*ftsZ*-Myc *ezrA*-HA) and IU9723 (Δ*mapZ ftsZ*-Myc *ezrA*-HA) grown in BHI to mid exponential phase (OD_{620}≈0.1-0.2) prepared for IFM as described in *Materials and Methods*. Data were obtained from two independent biological replicates. (B) Averaged images with fluorescence intensity traces showing FtsZ and EzrA localization in wild-type or Δ*mapZ* cells. Cells were binned into division stages 1-4, and images from the indicated number of cells (*n*) from at least two independent biological replicates were averaged using IMA-GUI program as described in *Materials and Methods.* For stage 1-4 cells, the Z- and EzrA-band were placed so that the shorter distance to the pole was on the right half of the chart, resulting in fluorescence intensity distributions being biased toward one cell half. Row 1, cell shapes determined from phase-contrast images; row 2, nucleoid locations from DAPI labeling; row 3, EzrA locations from IFM; row 4, FtsZ locations from IFM; row 5, normalized mean fluorescence intensity distributions along the horizontal cell axis for each channel (black, phase image; blue, DNA; green, EzrA; red, FtsZ). Data were obtained from two independent biological replicates. (C) Scatter plot of the paired widths from the same cells of FtsZ and EzrA fluorescent immunolabeled regions at the actively dividing septa of strain IU7223 at division stages 1-3. Width measurements and plotting were done using IMA-GUI program (see *Materials and Methods*). Statistical analysis was performed as described previously (Tsui *et al.,* 2014) where ** and *** indicate $P<0.01$ and $P<0.001$ respectively. Septal widths of stage 4 cells were not analyzed, because FtsZ or EzrA may have been missing from old sites of septation.

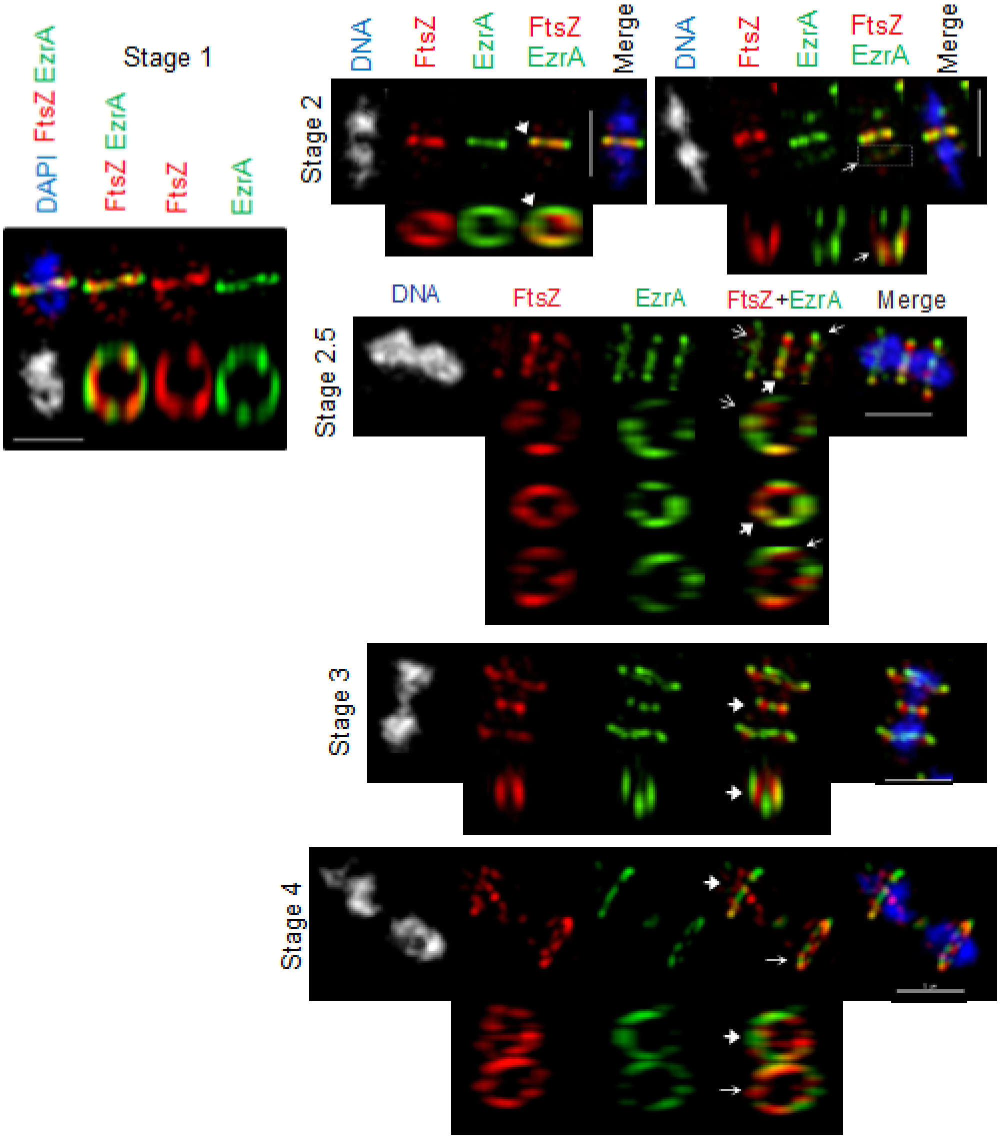

Supplementary Figure 3. Representative 3D-SIM IFM and DAPI images obtained of *Spn* strain IU7223 (FtsZ-Myc EzrA-HA) at different division stages. DNA (DAPI stained image) is false-colored white or blue in columns 1 or 5, respectively. FtsZ and EzrA are pseudo-colored as red and green, respectively. The first row of each panel represents images captured in the XY plane, while second row images were obtained by rotating a section of the cell around the X or Y axis. Individual rotated daughter and/or septal rings are indicated by the corresponding arrows of the non-rotated cells.

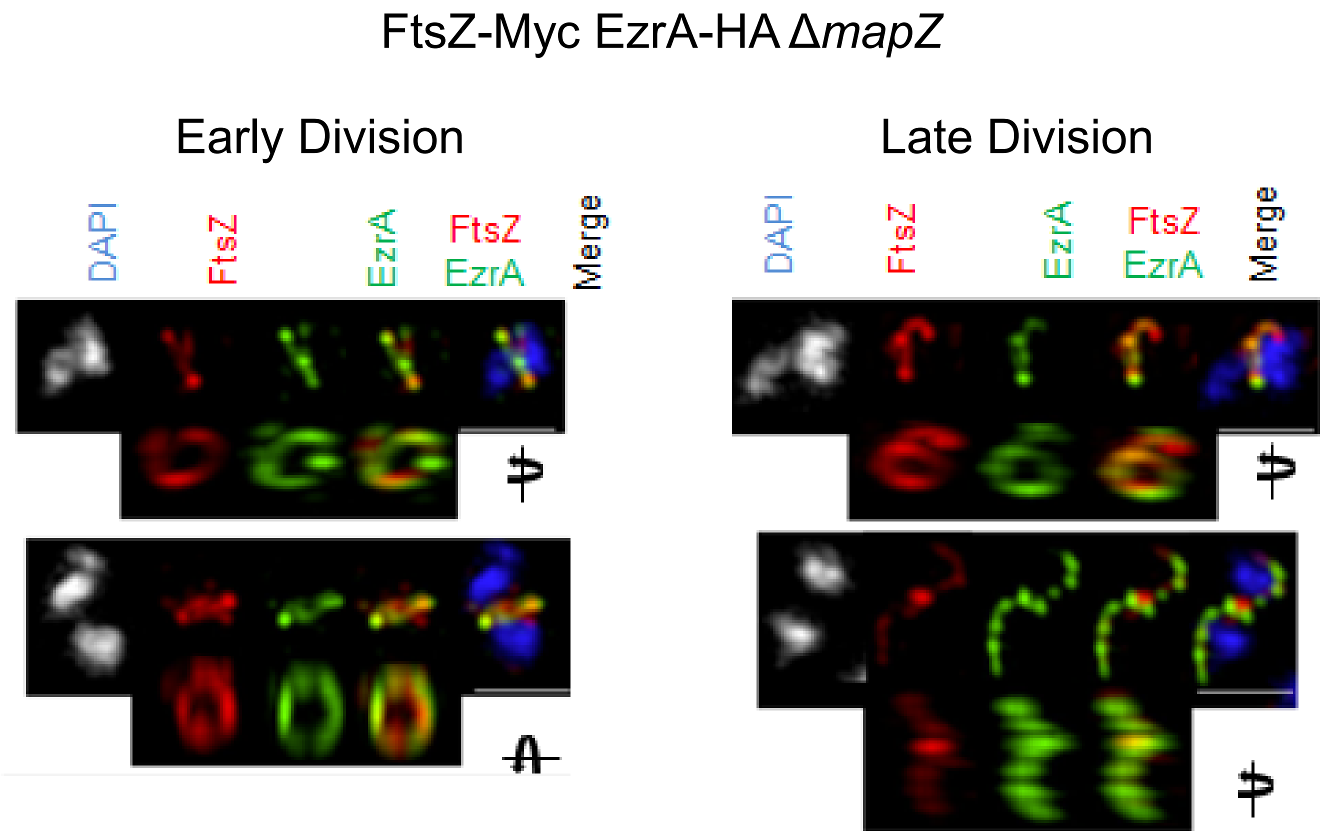

Supplementary Figure 4. Representative 3D-SIM IFM and DAPI images obtained from *Spn* strain IU9723 (Δ*mapZ* FtsZ-Myc EzrA-HA) in early or late divisional cells (panels on left or right, specifically). DNA (DAPI stained image) is false-colored white or blue in columns 1 or 5, respectively. FtsZ and EzrA are pseudo-colored as red and green, respectively. The first row of each panel represents images captured in the XY plane, while second row images were obtained by rotating the cell around the X or Y axis.

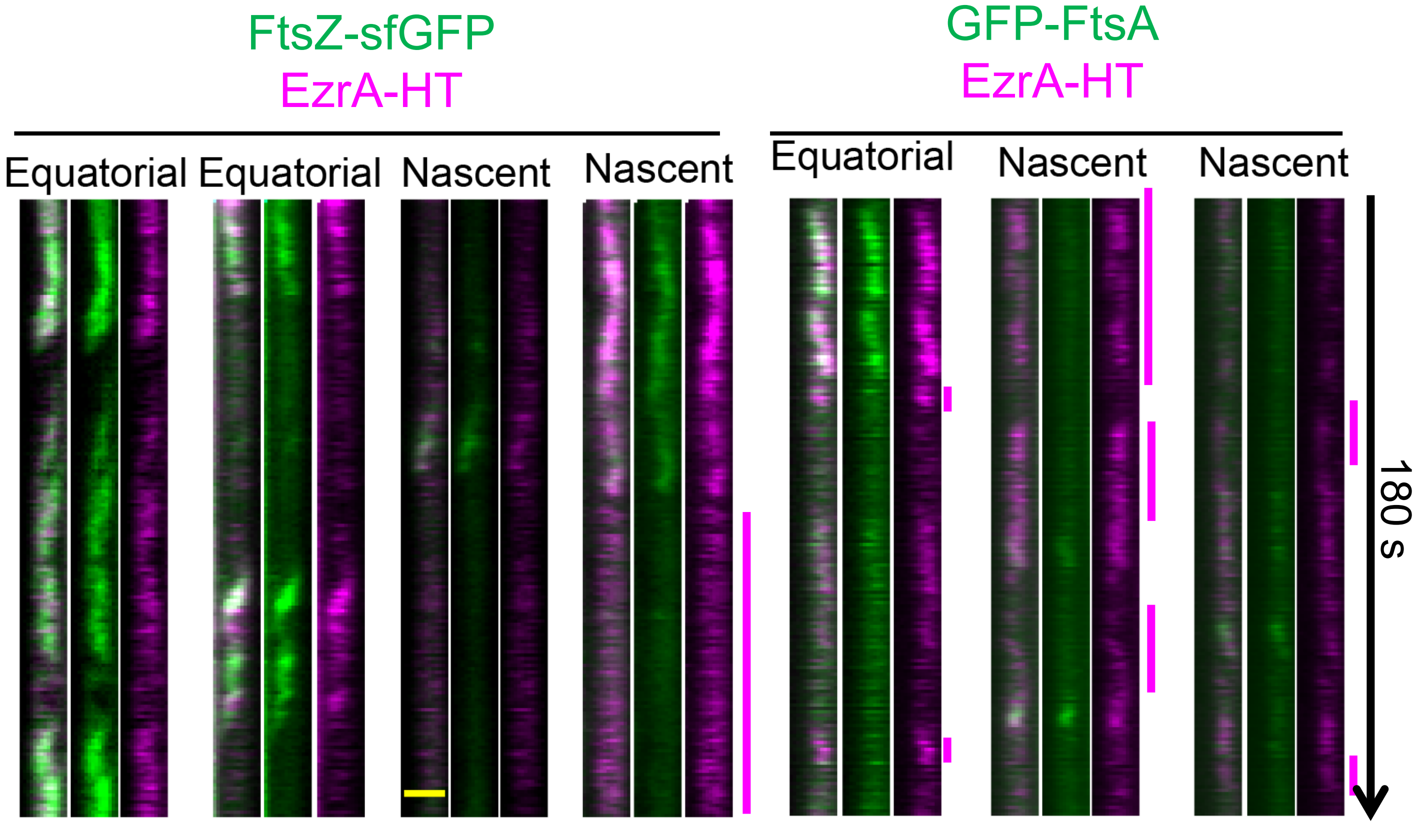

Supplementary Figure 5. Dual-TIRFm showing kymographs of nascent or early equatorial ring planes from time lapse experiments performed to show FtsZ with EzrA or FtsA with EzrA dynamics in *Spn*. Experiments were performed with strains IU15768 or IU15699 (See Supplementary Table 1 for complete genotypes). Kymographs were obtained from 180 frames, acquired at 1 frame/s intervals. Both strains were labeled with 500 nM HT-JF549 to label EzrA-HT. Scale bar is shown as the horizontal yellow bar (3rd set of kymographs from the left) and indicates 1 μm in size. Magenta drawn lines going vertical are regions where EzrA is present with a lack of FtsZ or FtsA observed. Kymographs are representative of 2 biological experiments in which greater than 5 nascent or 5 equatorial ring planes were analyzed.

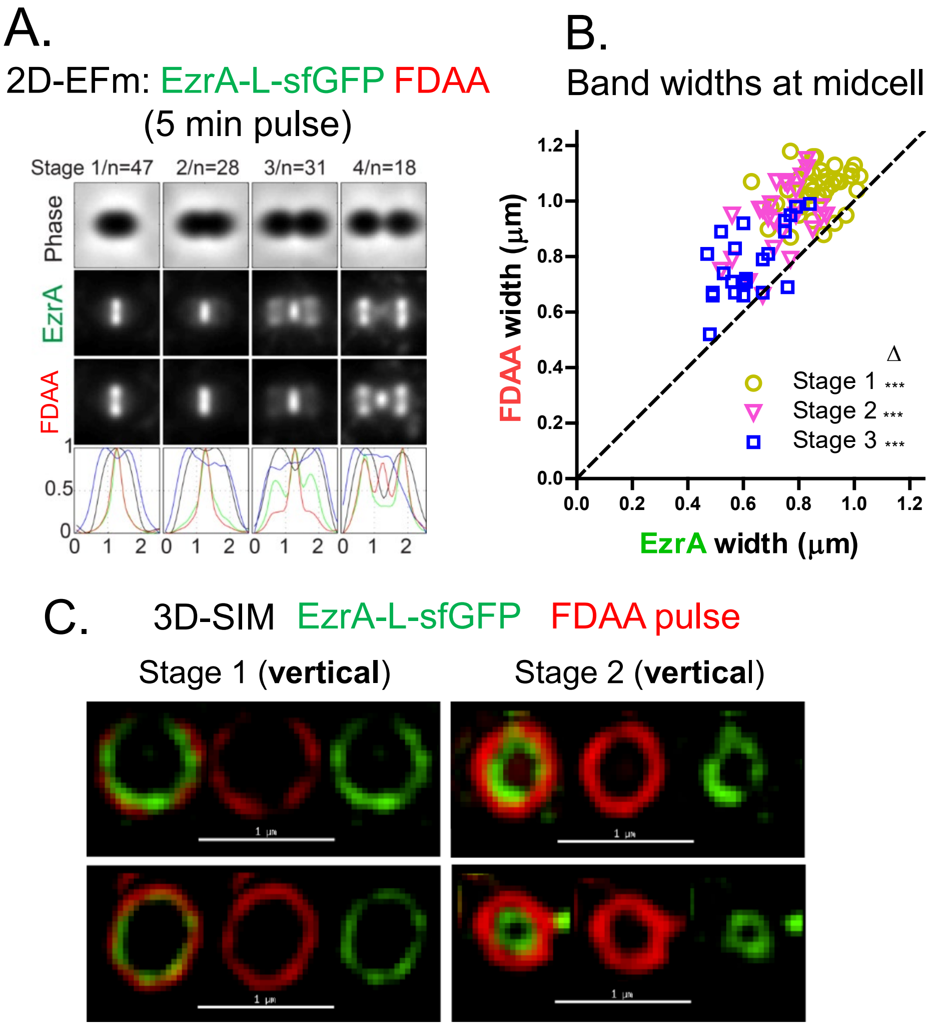

Supplementary Figure 6. Organization of EzrA and PG synthesis during the cell cycle of *Spn*. Pneumococcal strain expressing EzrA-sfGFP as the only source of EzrA in the cell (IU10254; *ezrA-sfgfp*) were grown exponentially and pulse-labeled with TADA for 5 min and as described in *Materials and Methods*. For 3D-data, a total of between 5-10 cells per stage were analyzed. EzrA is green while FDAA labeling (TADA) is pseudo-colored red. (A) 2D-analysis of EzrA-sfGFP and FDAA labeling using IMA-GUI program as described in *Materials and Methods* and previously (Tsui *et al.,* 2014). (B) Width measurement of EzrA-sfGFP rings and FDAA-rings from cells in 2D-fields. Measurements and plotting occurred as described previously (Tsui *et al.,* 2014) (C) 3D-SIM representative images of EzrA-sfGFP rings and FDAA labeling of vertically-oriented cells.

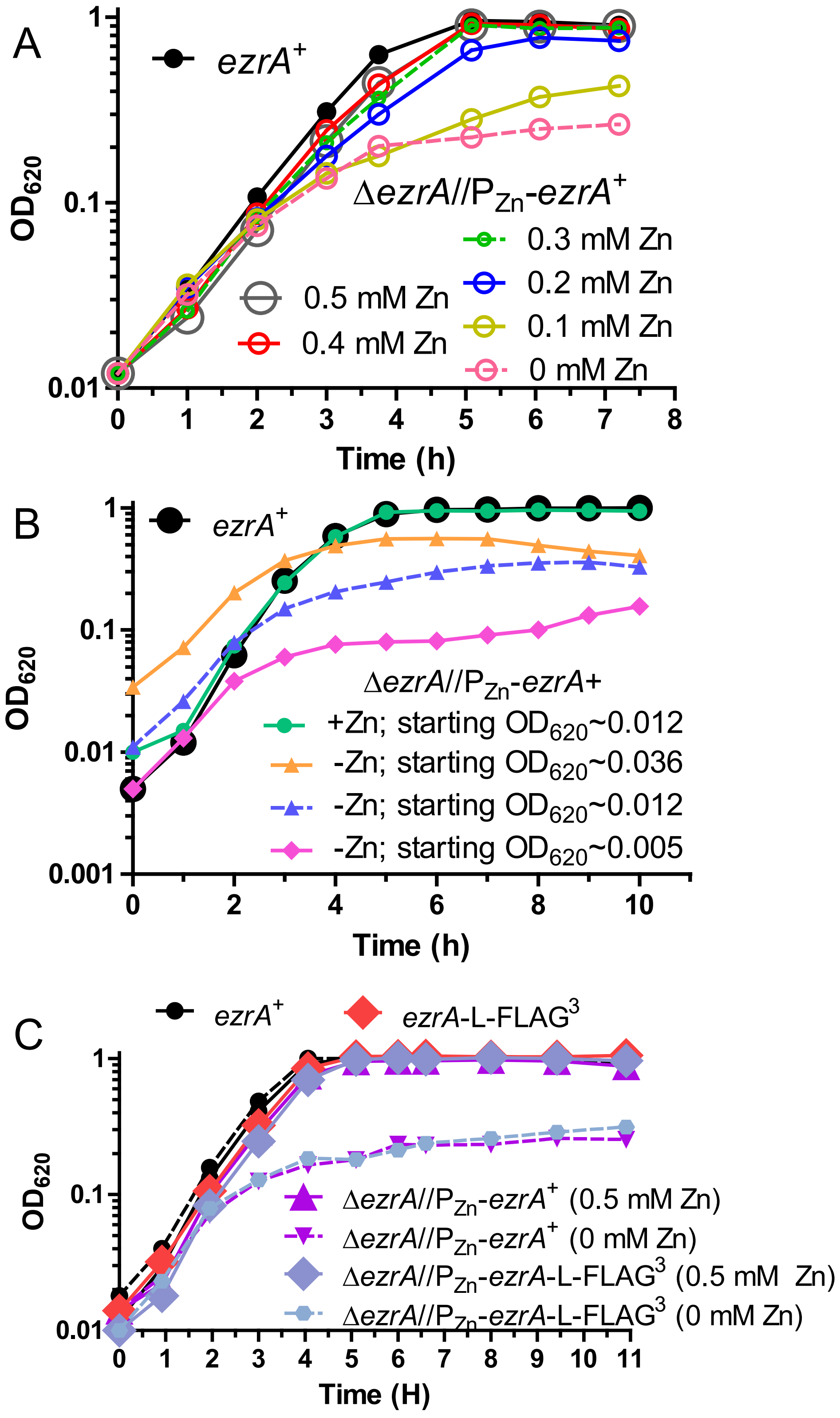

Supplementary Figure 7

Supplementary Figure 7. Depletion of EzrA is different ways shows EzrA is essential for *Spn* cell growth. Pneumococcal cells were depleted of EzrA (IU8799; Δ*ezrA*//*bgaA*::P_{Zn}-*ezrA*$^{+}$) and compared to IU1945 (*ezrA*$^{+}$). Shown are representative experiments from two or more biological replicates. (A) Growth curves showing induction using different amounts of $ZnCl_2$/$MnSO_4$ increases growth rate and final cell density yield. (B) Growth curves showing EzrA is required for wild-type like cell growth and final cell density in BHI broth by changing the cell density at the initiation of depletion. (C) Depletion of EzrA-FLAG^{3} in IU9572 occurs similarly to depletion of EzrA^{+} in IU8799.

Pre-divisional cells

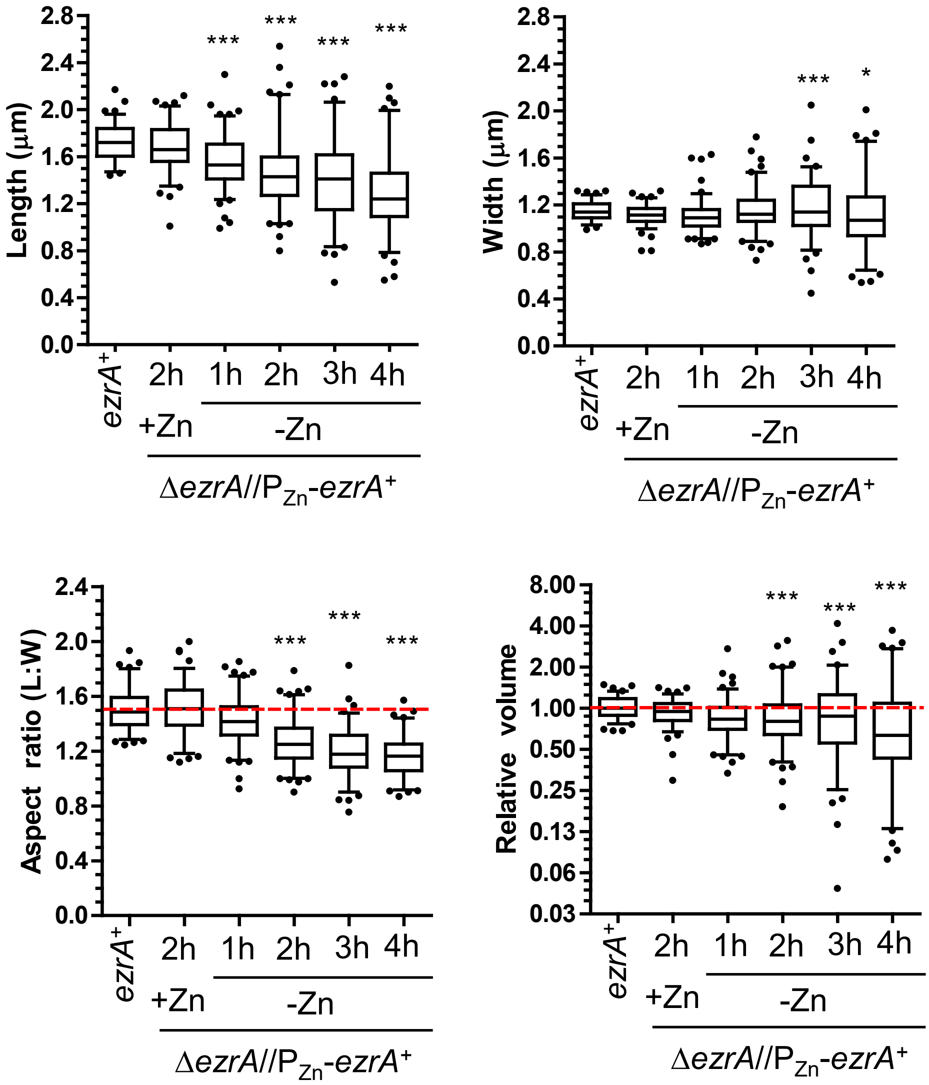

Supplementary Figure 8. EzrA(*Spn*) depletion results in shape and size aberrances. Box-and-whiskers plots (whiskers, 5 and 95 percentile) showing quantification of lengths, widths, aspect ratio (Length/Width), and relative volumes (W^2 x L) of EzrA depletion strain (IU8799; Δ*ezrA*//*bgaA*::P_{Zn}-*ezrA*+) compared to that of wild-type (IU1945; *ezrA*+). Length was defined as the longer side of stage 1 cells or half of the longest axis of stage 4 cells such that the measurement is that of the daughter-cell, individually. The Width was defined as the shorter axis of stage 1 cells or at equatorial-parallel planes of stage 4 daughter cells. Volumes are relative to the median volume of wild-type cells (IU1945). The red dotted line in "Aspect ratio" and "Relative volume" indicated the median of wild-type cells. P values were obtained by one-way ANOVA analysis between WT and other samples (GraphPad Prism, nonparametric Kruskal-Wallis test). (P<0.05 indicated by *, P<0.001 indicated by ***). P values are for comparison against IU1945 (*ezrA*+).

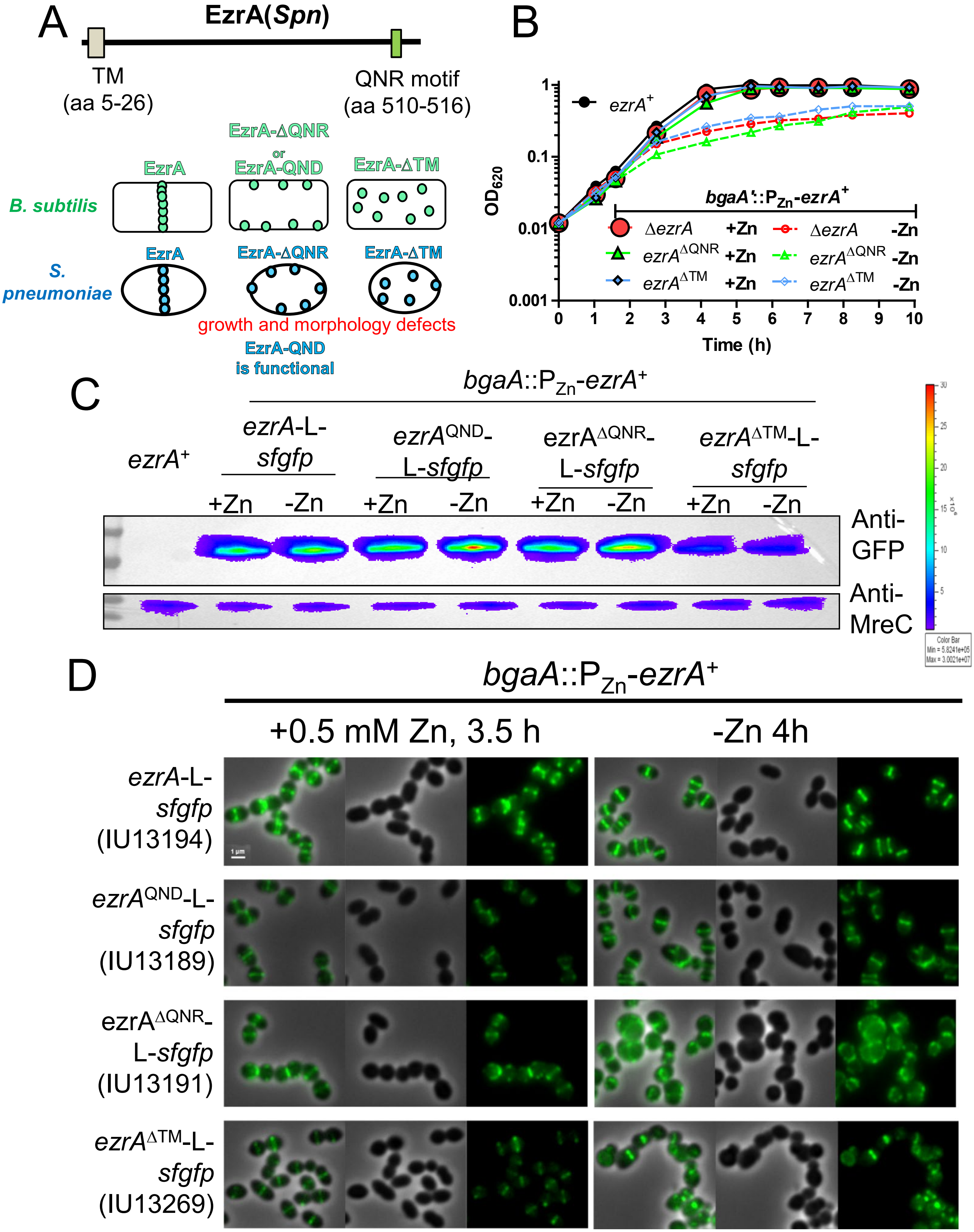

Supplementary Figure 9

Supplementary Figure 9. The transmembrane domain and QNR motif of EzrA(*Spn*) are required for protein function and midcell localization. (A) 2D-structure of EzrA showing the amino acids composing the transmembrane (TM) domain and the QNR motif and schematic showing different effects of EzrA domain mutants as reported in (Haeusser *et al.,* 2007; Land *et al.,* 2014). In *S. pneumoniae*, deletion of the QNR motif or TM domain is lethal. (B) Depletion of ectopic $EzrA^+$ in Δ*ezrA*, *ezrA*ΔQNR, or *ezrA*ΔTM mutant backgrounds. Strains used IU1945, IU8799, IU10909, IU11123. (C) Western blots detecting EzrA-sfGFP variants (using anti-GFP) or MreC loading control (using anti-MreC) as described in *Materials and Methods*. 3 μg of cell lysate was loaded per lane. (D) Localization of EzrA-sfGFP variants in cells grown in the presence of Zn (0.5 mM $ZnCl_2$ and 0.05 mM $MnSO_4$) or depleted of Zn (-Zn). Cells were imaged at T=4 h. The strains used are indicated in the figure. The fluorescence intensity of EzrA(ΔTM)-sfGFP was enhanced 2X to show localization of this protein as it demonstrated less fluorescence intensity in comparison to all other fusions shown here.

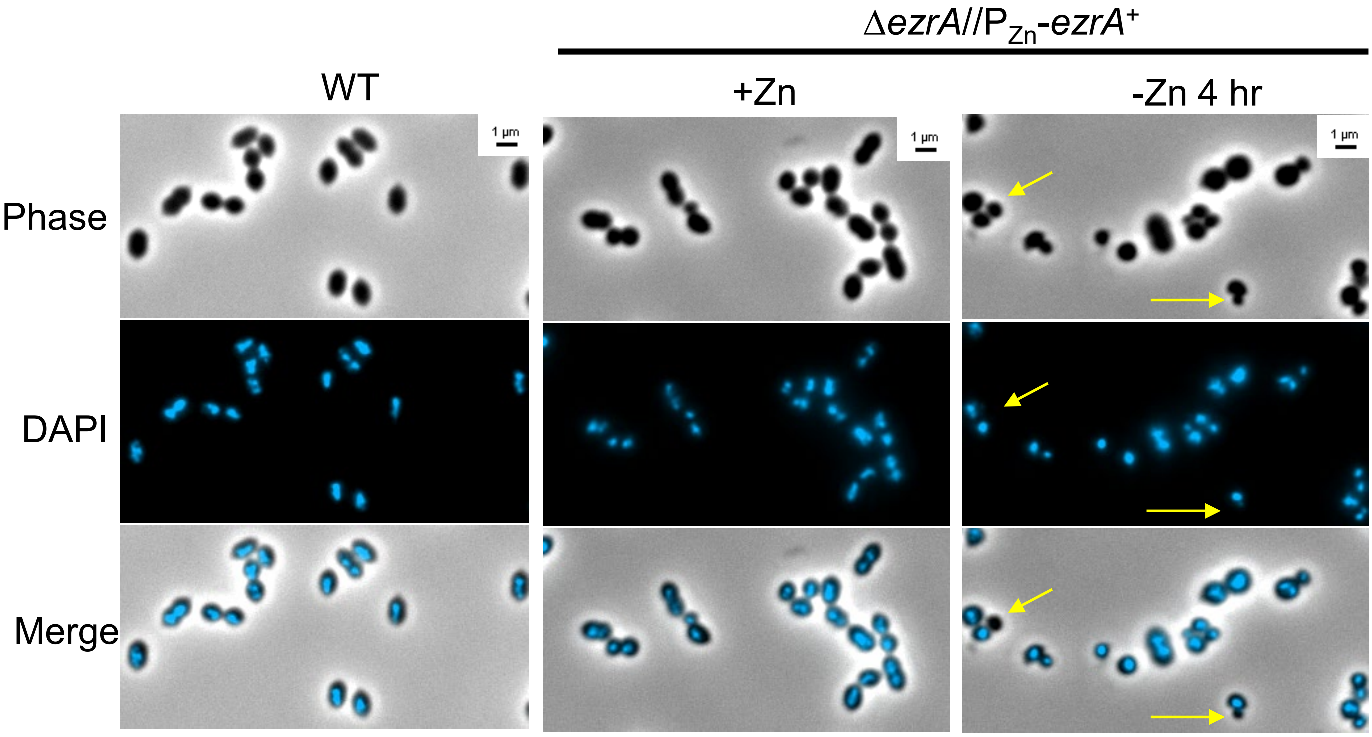

Supplementary Figure 10. Chromosome segregation defects upon EzrA(*Spn*) depletion. Exponentially growing cells (IU1945 or IU8799) were fixed and stained with DAPI as described in *Materials and Methods*. Pre-divisional or post-divisional cells were identified based on phase contrast microscopy then overlaid with DAPI and scored as nucleate (containing DAPI staining) or anucleate (lacking DAPI staining). Arrows point to anucleate cells.

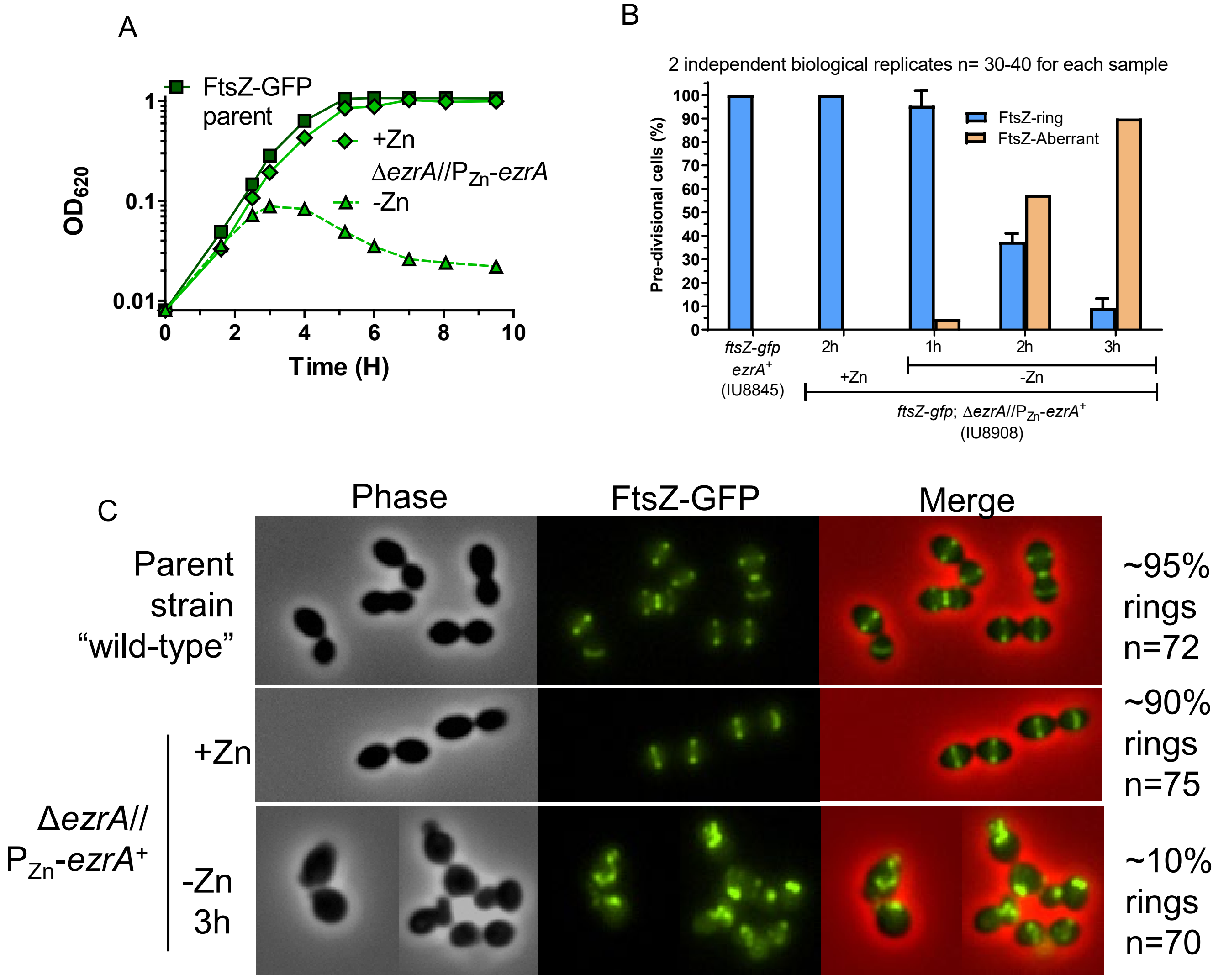

Supplementary Figure 11. Depletion of EzrA(*Spn*) in a strain expressing FtsZ-GFP. Growth and FtsZ-GFP localization was compared in IU8845 (FtsZ-GFP parent) or IU8908 (FtsZ-GFP in EzrA depletion background), in cells grown in BHI broth at 37° C, see Supplementary Table 1 for full genotypes. (A) Growth curve (B) Quantitation of FtsZ-ring or aberrances in pre-divisional cells. (C) Representative images of WT or EzrA depleted cells expressing FtsZ-GFP. Experiment was performed twice with similar results.

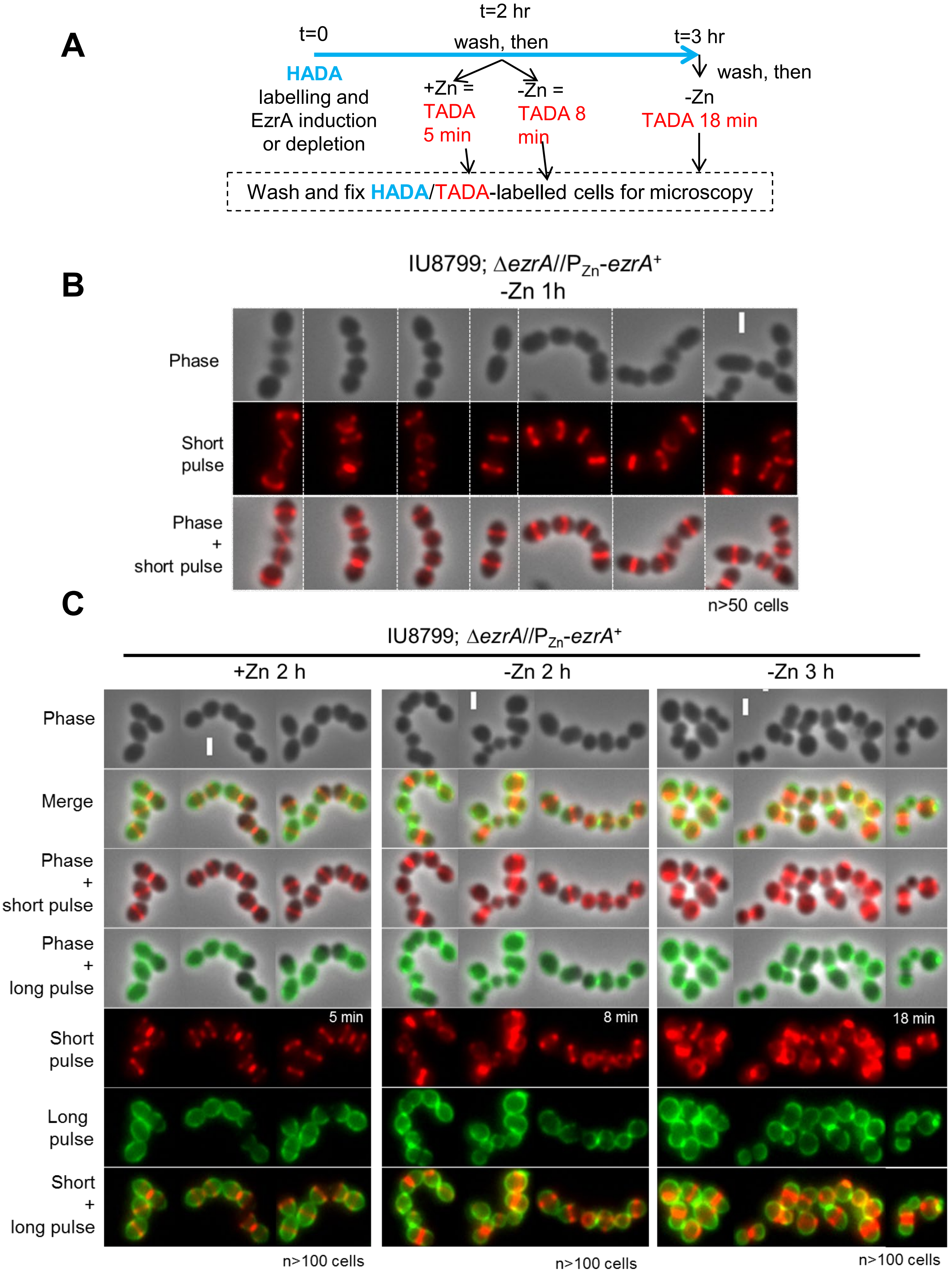

Supplementary Figure 12

Supplementary Figure 12. 2D-EFm of FDAA labeled EzrA-depleted (*Spn*) cells shows aberrant or absence of FDAA-rings in equators of future dividing cells. (A) Schematic of FDAA labeling procedure. IU8799 (Δ*ezrA*//*bgaA*::P_{Zn}-*ezrA*$^{+}$) was grown exponentially, and depleted of EzrA by shifting cells to BHI broth lacking $ZnCl_2$ and $MnSO_4$ as described in *Materials and Methods.* Pre-labeling with FDAA HADA (pseudo-colored blue), pulse labeling with FDAA TADA (pseudo-colored red), fixation, and imaging were performed as described in *Materials and Methods* with the indicated procedures at different time points. (B) EzrA depletion showing 2D representative images of FDAA labeling in EzrA depleted strain (at 1 h) with 5 minute short pulse labeling time. (C) EzrA depletion showing 2D representative images of FDAA-labeled EzrA-complemented or -depleted cells (at 2 or 3 h) with respective short pulse labeling time indicated by values in the fifth row. Long pulse is pseudo colored green to shown better contrast. Scale bars are 1 μm.

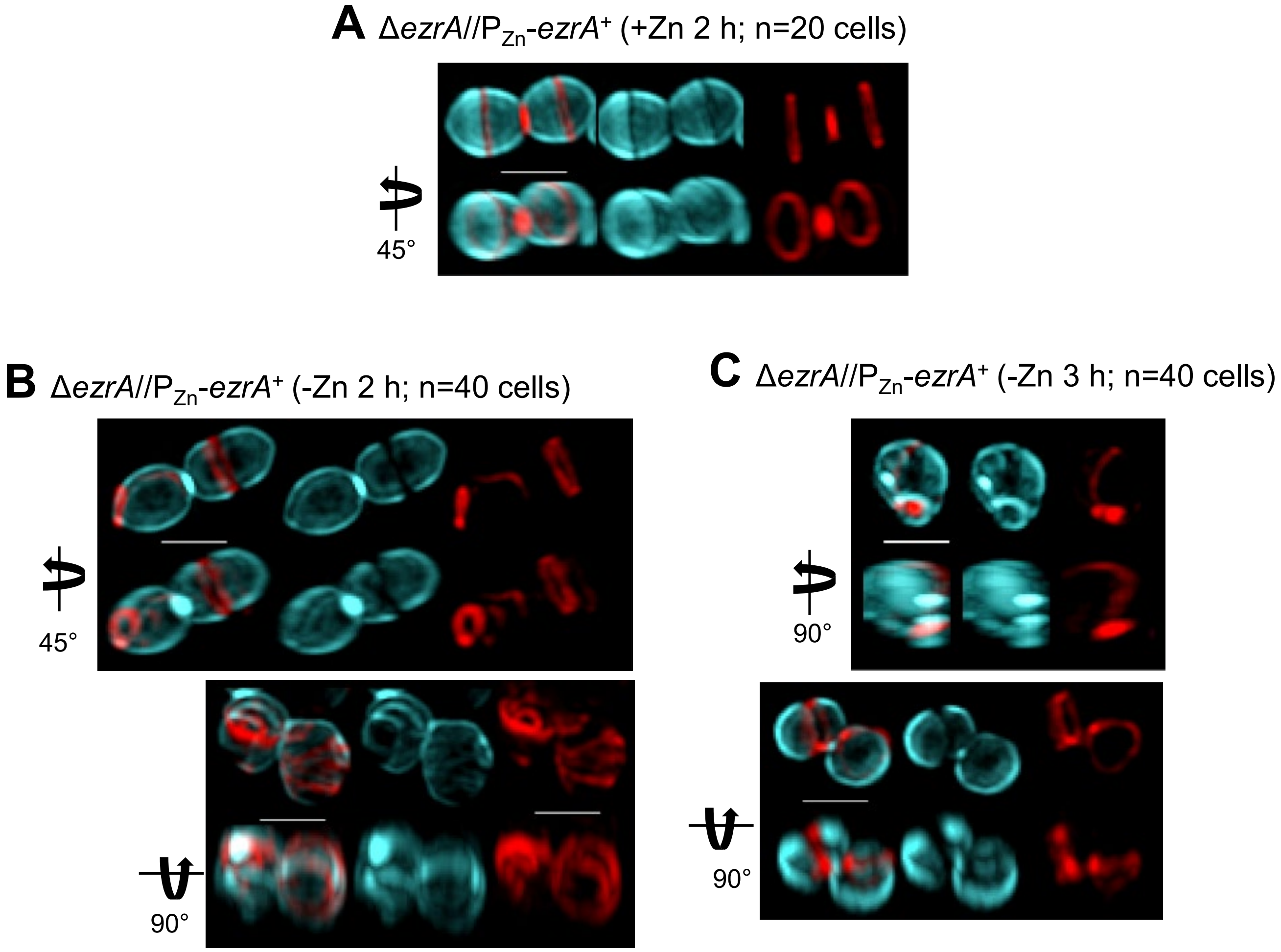

Supplementary Figure 13. Representative 3D-SIM images of FDAA pulse-chase labeled *Spn* cells show EzrA depletion in strain IU8799 (Δ*ezrA*//*bgaA*::P$_{Zn}$-*ezrA*$^{+}$) leads to major aberrances in new FDAA insertion locations. EzrA depleted cells were obtained at appropriate time points and chase labeled with TADA as indicated in Supplementary Figure 12A and in *Materials and Methods*. Red indicates new chase-labeling while cyan indicates pulse cell wall labeling as described in *Materials and Methods*. At least 30 cell were analyzed in each case (A) Complemented EzrA strain shows normal midcell FDAA labeling and FDAA-ring labeling at equators of future dividing daughter cells (bottom rows rotated 45º around the Y-axis). (B) EzrA depletion at 2 h. Top, FDAA-rings are placed at cell pole (left daughter cell) or at midcell (right daughter cell) (bottom row rotated 45º around the Y-axis). Bottom, FDAA labeling displays aberrant ring-like structures (left cell) or dispersed pattern (right cell) (bottom row rotated 90º around the X-axis) (C) EzrA depletion at 3 h. Top, foci of new-red and old-blue labeling (bottom row rotated 90 around the Y-axis). Bottom, FDAA-rings are placed in perpendicular planes of adjacent cells (bottom row rotated 90º around the X-axis).

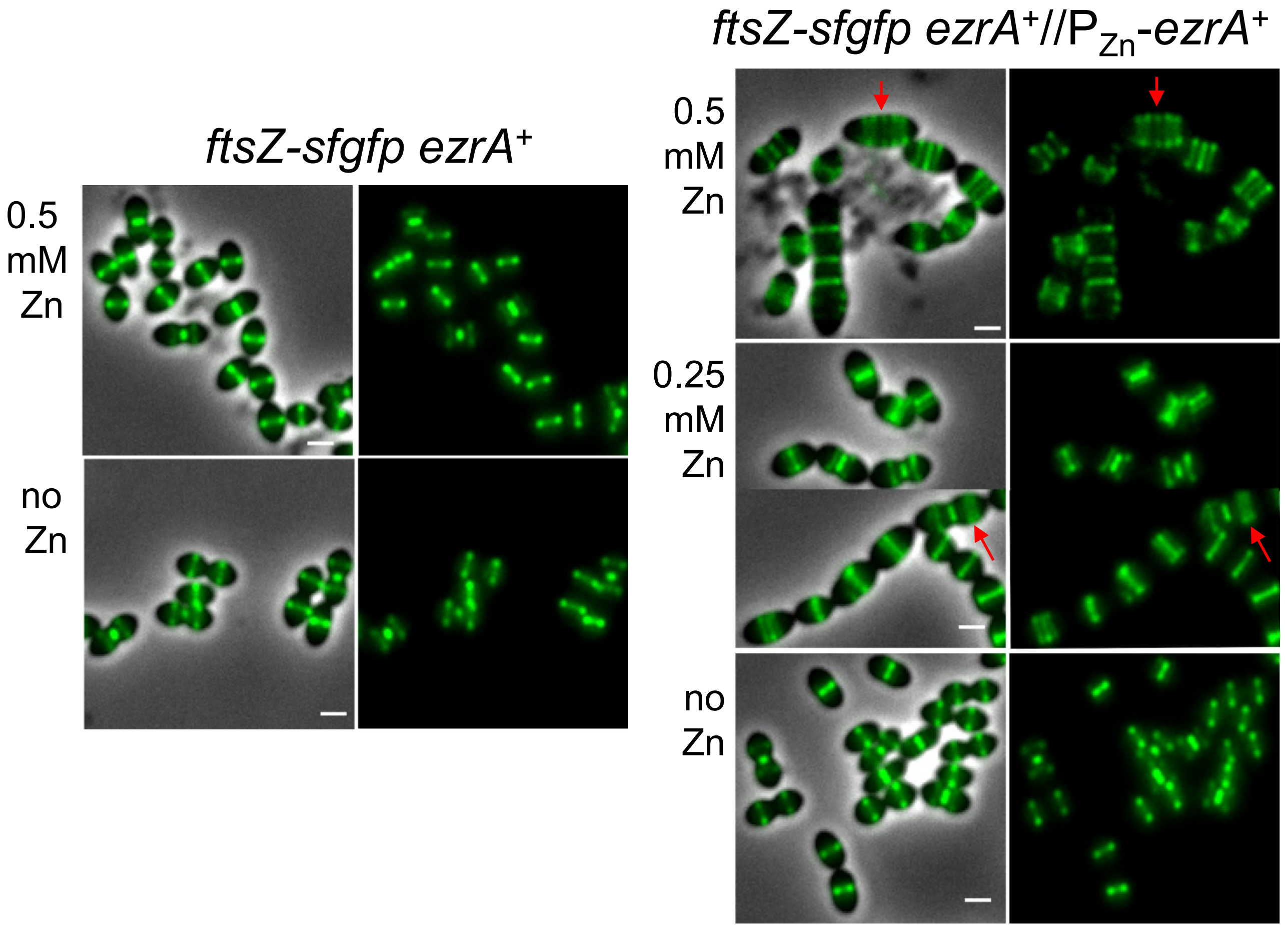

Supplementary Figure 14. Overexpression of EzrA leads to extra Z-rings in *S. pneumoniae*. FtsZ-sfGFP was localized in IU9985 (*ftsZ-sfgfp ezrA*$^+$) or *ezrA* merodiploid strain IU14224 (*ftsZ-sfgfp ezrA*$^+$//P_{Zn}-*ezrA*$^+$) cultured in C+Y (pH 6.9-7.1) media in a 5% CO_2 incubator at 37°C. Cells were grown from OD_{620}≈0.003 without supplemented $ZnCl_2$ (no Zn) or supplemented with $ZnCl_2$ (0.5 mM $ZnCl_2$ or 0.25 mM $ZnCl_2$) and $MnSO_4$ (see *Materials and Methods*) for 4 hours prior to imaging. Images are representative of two independent biological replicates. Arrows point to cells with extra Z-rings.

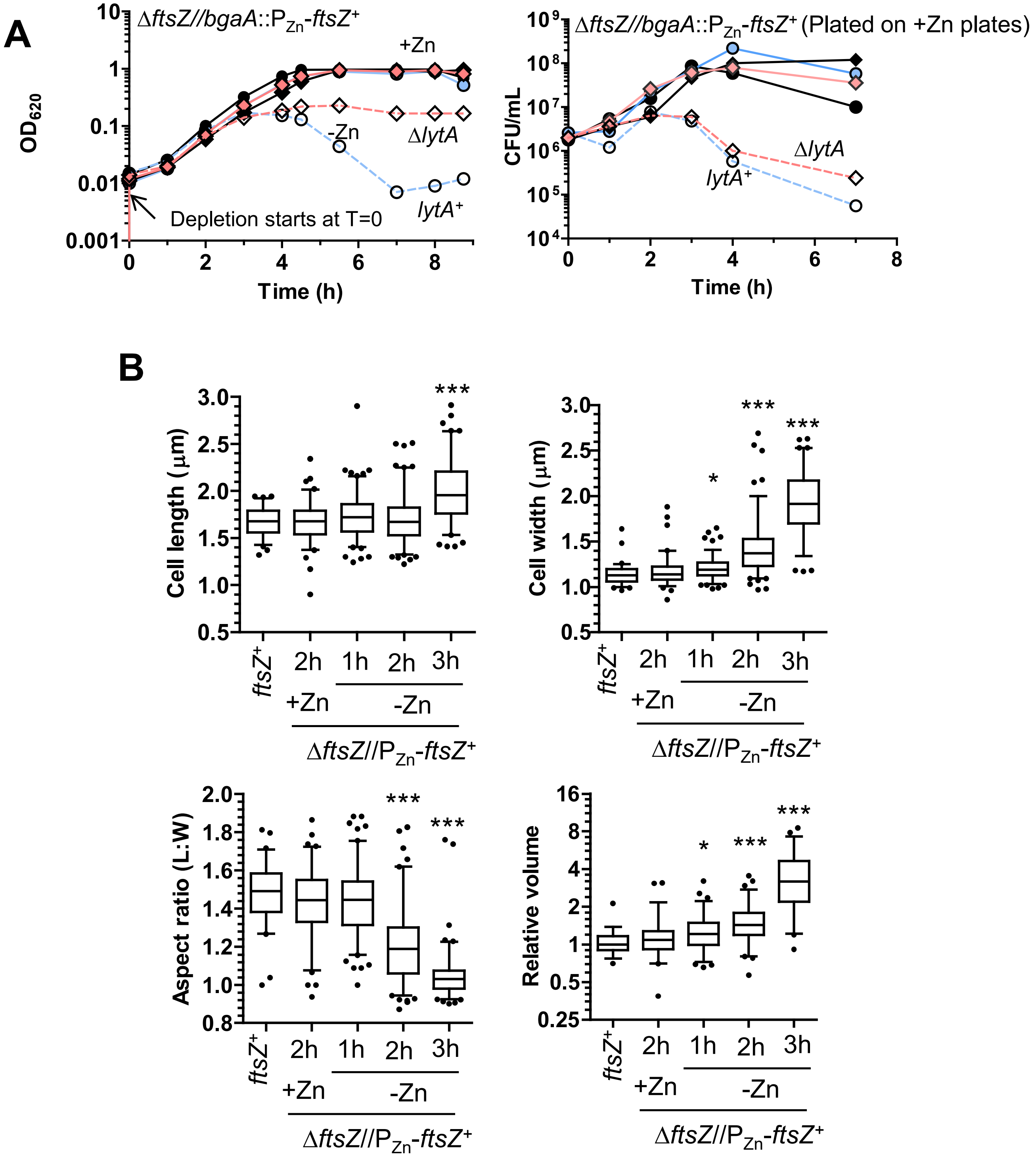

Supplementary Figure 15

Supplementary Figure 15. Bactericidal effect of FtsZ(*Spn*) depletion and enlarged spherical cell morphology due to FtsZ-depletion. (A) Growth curve in BHI broth and corresponding quantification of CFU/mL of FtsZ complemented or depleted cultures. Samples were obtained at T=0, 1, 2, 3, 4, and 7 h from the WT (black line), FtsZ complemented (filled lines) or FtsZ depleted cultures (dotted lines) serially diluted where appropriate, and 5 μL of serial dilutions were spotted on blood-agar plates supplemented with 0.3 mM $ZnCl_2$ and 0.03 mM $MnSO_4$, and analyzed for CFU. Strains used were IU1945 (black circles), E43 (Δ*lytA* control; black diamonds), IU8124 (blue circles), and IU8810 (pink diamonds). Experiment was performed twice with similar results. (B) Box-and-whiskers plots (whiskers, 5 and 95 percentile) of cell lengths, widths, aspect ratio (Length/Width), and relative volumes (W^2 x L) of FtsZ depletion strain (IU8124; Δ*ftsZ*//P_{Zn}-*ftsZ*$^+$) compared to that of wild-type (IU1945; *ftsZ*$^+$). Volumes are relative to the median volume of wild-type cells (IU1945). P values were obtained by one-way ANOVA analysis (GraphPad Prism, nonparametric Kruskal-Wallis test). ($P<0.05$ indicated by *, $P<0.001$ indicated by ***). P values are for comparison against IU1945 (*ftsZ*$^+$).

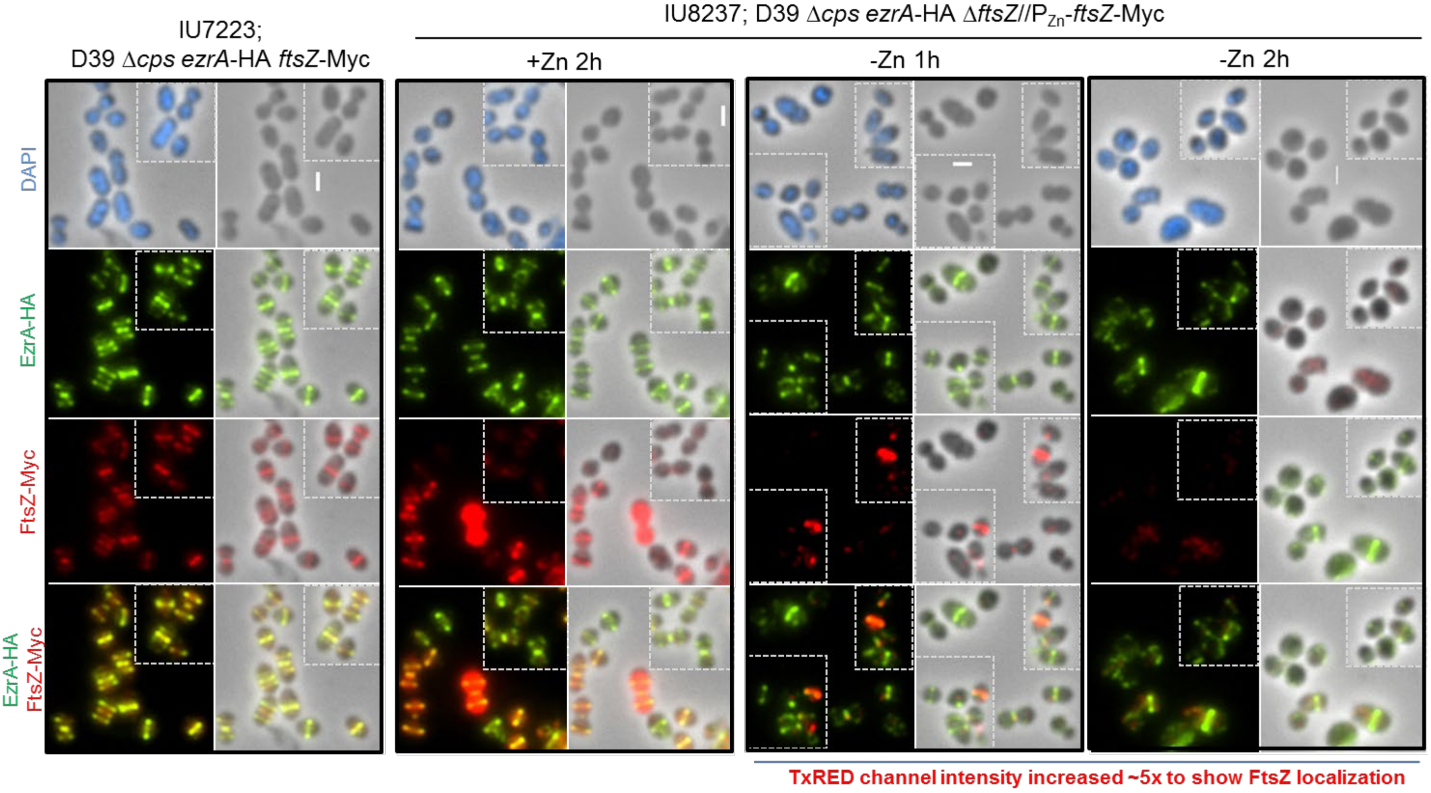

Supplementary Figure 16. Localization of EzrA and FtsZ in FtsZ-depleted *Spn* cells shown by IFM. Strain IU7223 (*ftsZ*-Myc *ezrA*-HA) and IU8237 (*ezrA*-HA Δ*ftsZ*//P_{Zn}-*ftsZ*-Myc) grown in BHI to mid exponential phase (OD_{620}≈0.1-0.2) and depleted of FtsZ-Myc where appropriate as described in *Materials and Methods*. Samples were processed for IFM with DAPI labeling of DNA as described in *Materials and Methods*. Texas red channel was manually increased to show FtsZ-Myc localization during FtsZ-Myc depletion at 1 and 2 h. Dotted boxes are indicative of additional cells that were added to show a greater number of cells in a montage format.

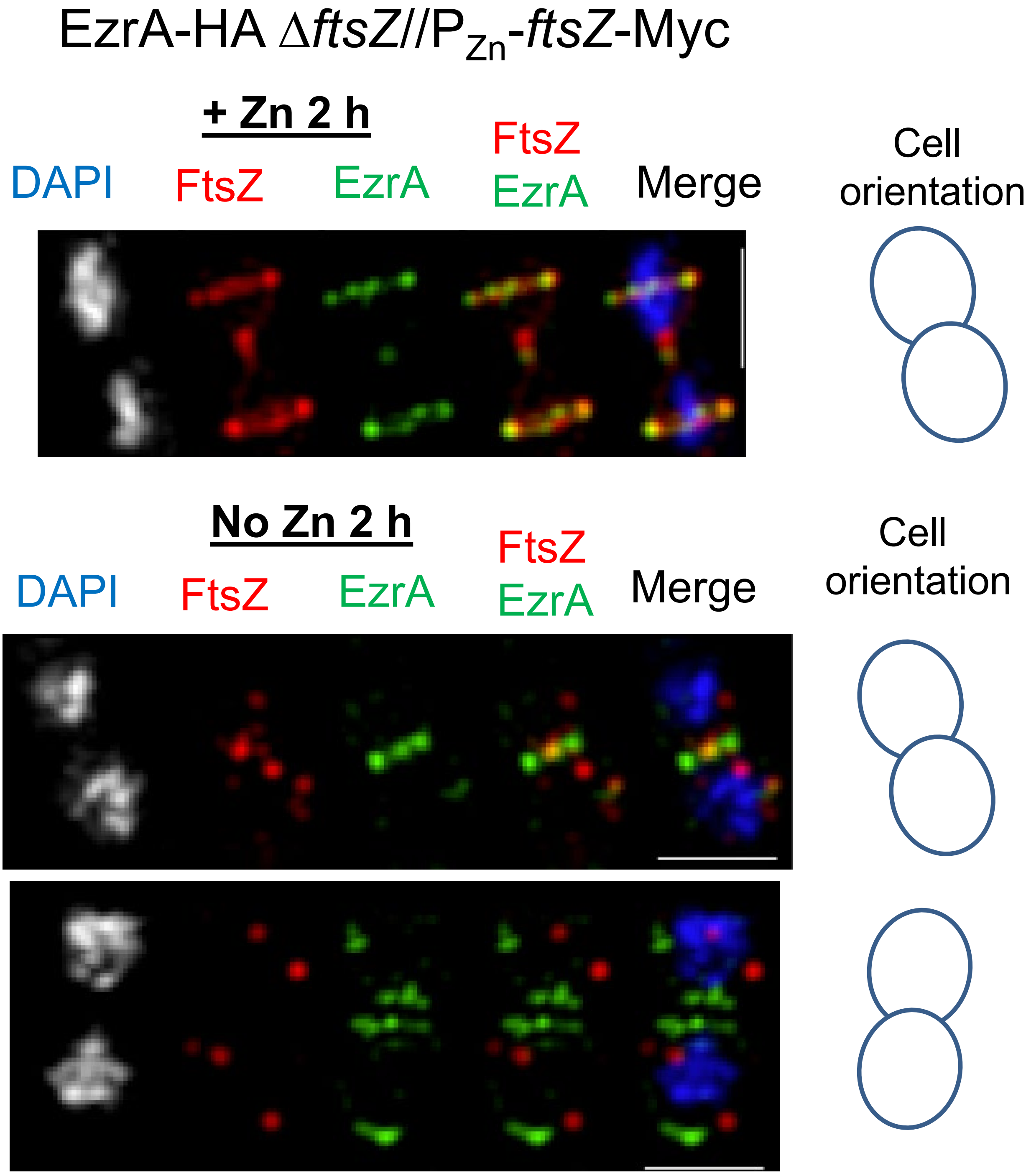

Supplementary Figure 17. 3D-SIM IFM shows EzrA becomes diffuse and aberrant when FtsZ(*Spn*) is depleted. FtsZ depletion strain IU8237 (*ezrA*-HA Δ*ftsZ*//*bgaA*::P_{Zn}-*ftsZ*-Myc), was grown exponentially, and was depleted (or complemented) of FtsZ-Myc by shifting cells to BHI broth not supplemented with additional $ZnCl_2$ and $MnSO_4$ as described in *Materials and Methods.* Cells were obtained at indicated time intervals and prepared for IFM as described in *Materials and Methods.* Experiments were performed twice with similar results. Top panel is representative of strain supplemented with $ZnCl_2$. bottom two panels are FtsZ depleted cells at T=2 h. "Cell orientations" are estimated cell outlines based on DAPI staining.

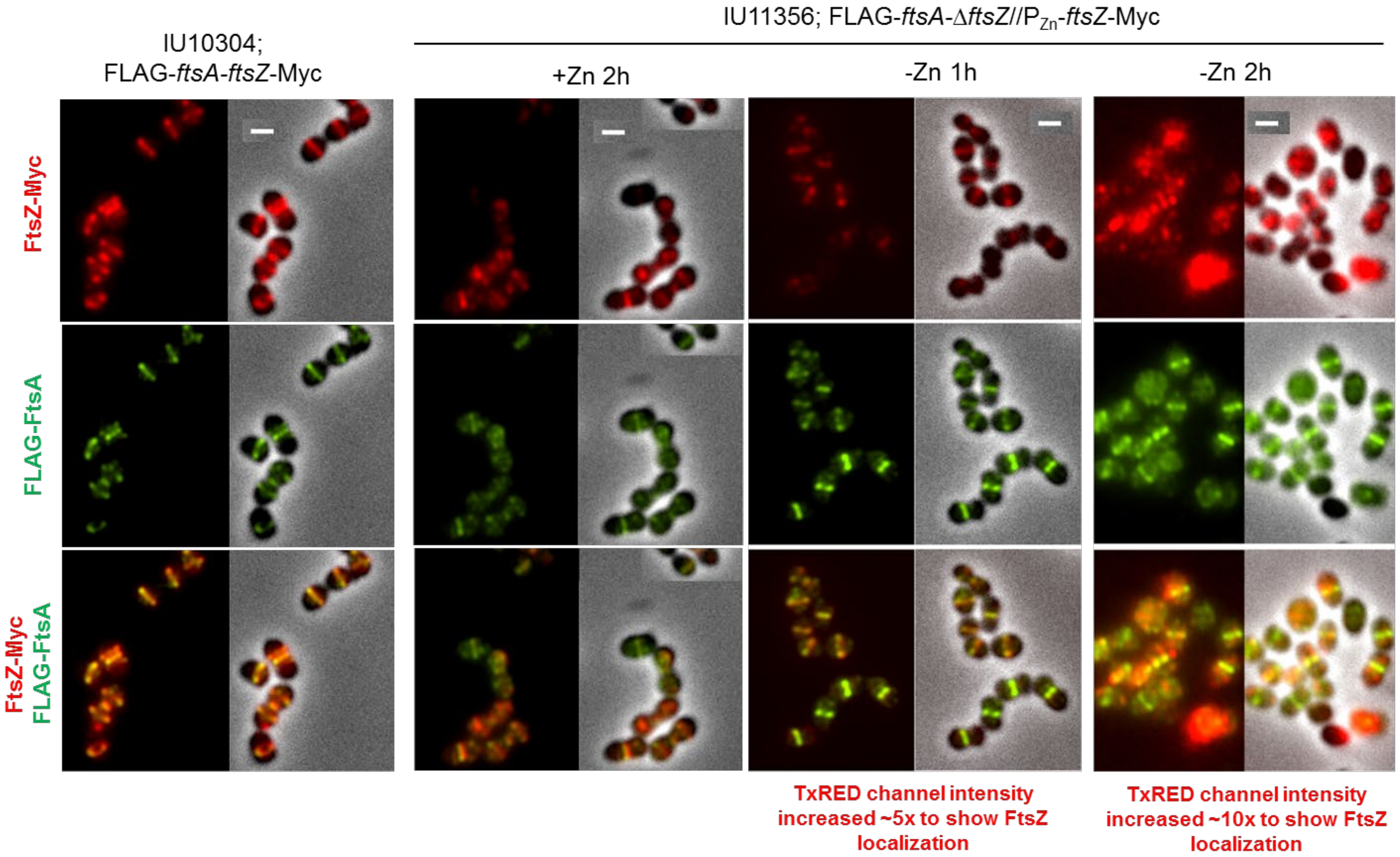

Supplementary Figure 18. Localization of FtsA and FtsZ in FtsZ-depleted *Spn* cells shown by IFM. Phase-contrast and 2D IFM images of representative fields of IU10304 (FLAG-FtsA_FtsZ-Myc) and IU11356 (FLAG-*ftsA ΔftsZ*//P_{Zn}*ftsZ*-Myc) cells grown in the presence of Zn (+Zn; 0.3 mM $ZnCl_2$ + 0.03 mM $MnSO_4$) or depleted of $ZnCl_2$ for the indicated amount of time (at T=1 or T=2 h). Data were representative of two independent biological replicates.

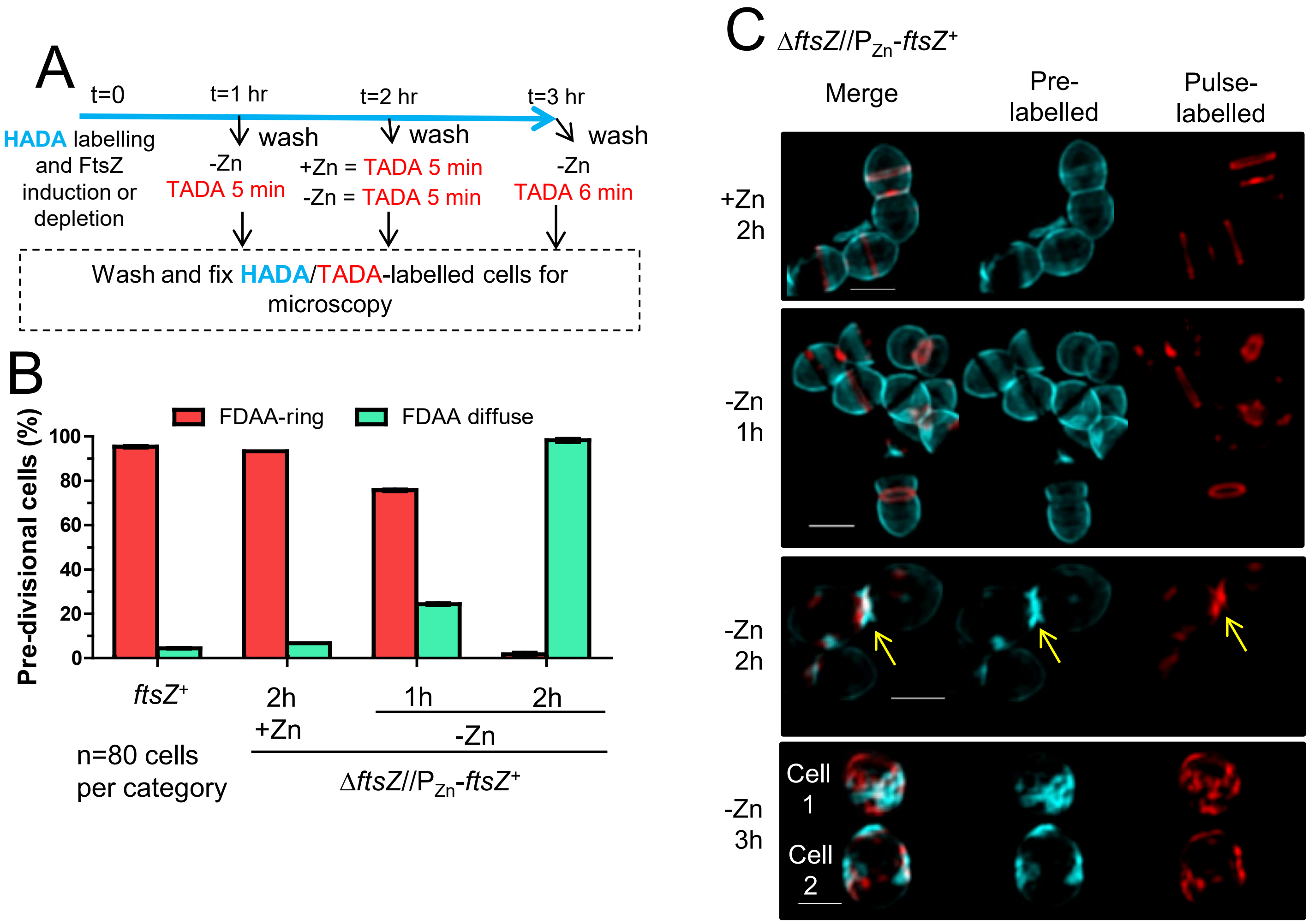

Supplementary Figure 19. FtsZ(*Spn*) is required for recruitment of FDAA labeling to equators of future dividing daughter cells. FDAA labeling in FtsZ complimented at 2 h or depleted strains (at 1, 2, or 3 h). FtsZ depletions and processing by cell fixation for microscopy occurred as described in *Materials and Methods.* (A) Schematic of labeling procedure during FtsZ depletion. IU8124 (Δ*ftsZ*//*bgaA*::P_{Zn}-*ftsZ*$^+$) was grown exponentially, and depleted of FtsZ by shifting cells to BHI broth lacking $ZnCl_2$ and $MnSO_4$ as described in *Materials and Methods.* Pre-labeling with FDAA HADA (pseudo-colored blue), pulse labeling with FDAA TADA (pseudo-colored red), fixation, and 3D-SIM were performed as described in *Materials and Methods* as indicated in the scheme. (B) Quantification of FDAA-ring structures in FtsZ-depleted pre-divisional or post-divisional cells which were processed for FDAA labeling. Cells were classified as containing FDAA-ring or FDAA diffuse. 40 cells were sorted per biological replicate. Error bars are the SEM from two independent biological replicates. (C) Representative 3D-SIM images of FDAA labeled cells described in (B). Each panel represents a different field of cells. Arrow points to old sites of division of stage 4 cells. More than 20 cells were analyzed via 3D-SIM per condition.

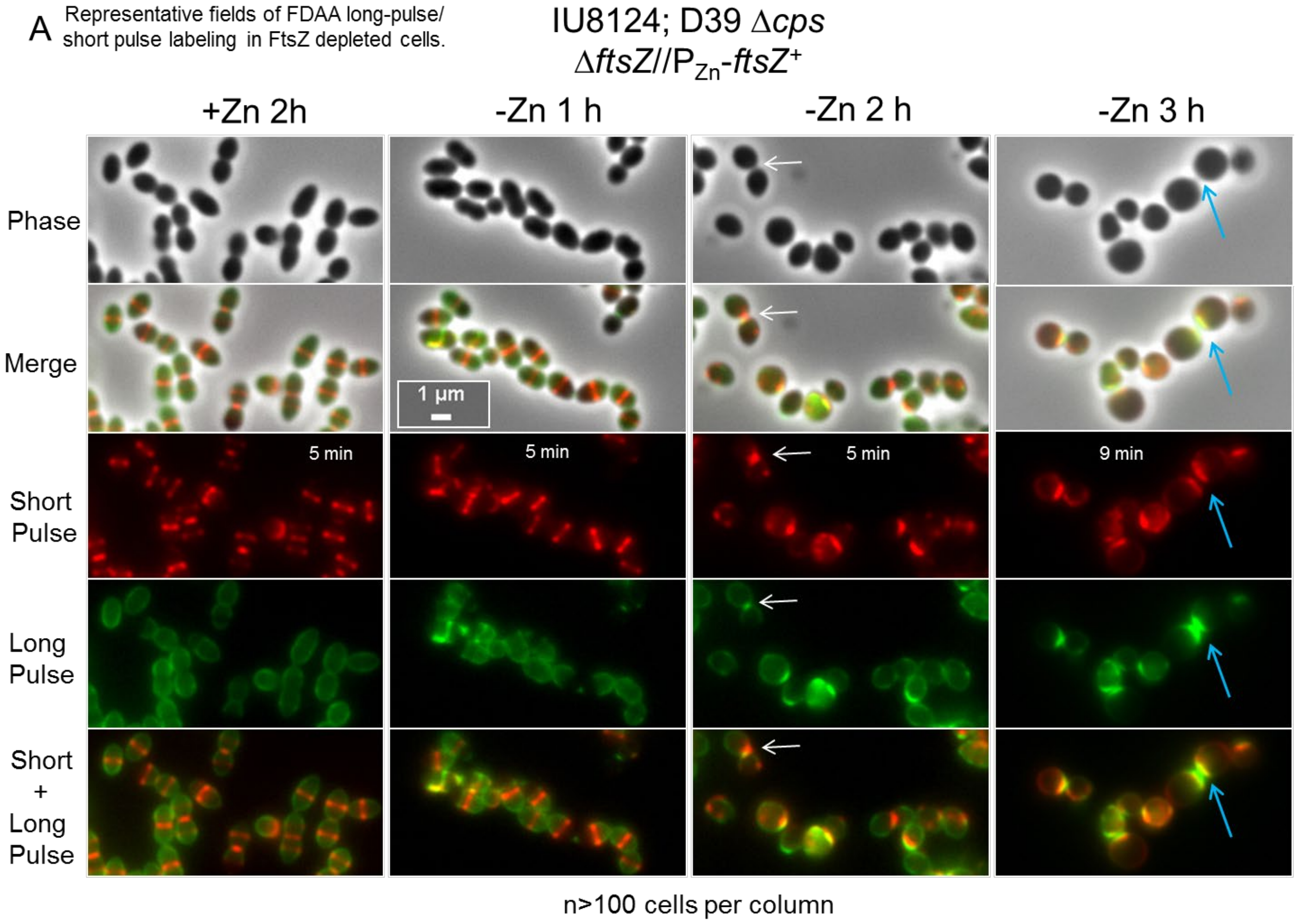

Supplementary Figure 20. 2D representative images of FDAA labeled FtsZ-depleted *Spn* cells. IU8124 (Δ*ftsZ*//*bgaA*::P_{Zn}-*ftsZ*$^{+}$) was grown exponentially in BHI broth in the presence of 0.3 mM $ZnCl_2$/ 0.03 mM $MnSO_4$, and was depleted of FtsZ by shifting cells to BHI broth with FDAA-HADA lacking $ZnCl_2$ and $MnSO_4$ for the indicated amount of time as described in Supplementary Figure 19A. The respective short pulse labeling (FDAA-TADA) times are indicated by values in the third row. Long pulse (FDAA-HADA) is pseudo colored green to shown better contrast. Arrows point to sites of PG syntheses between daughter cells that failed to properly localize to equatorial rings. More than 100 cells were analyzed for each condition (column).

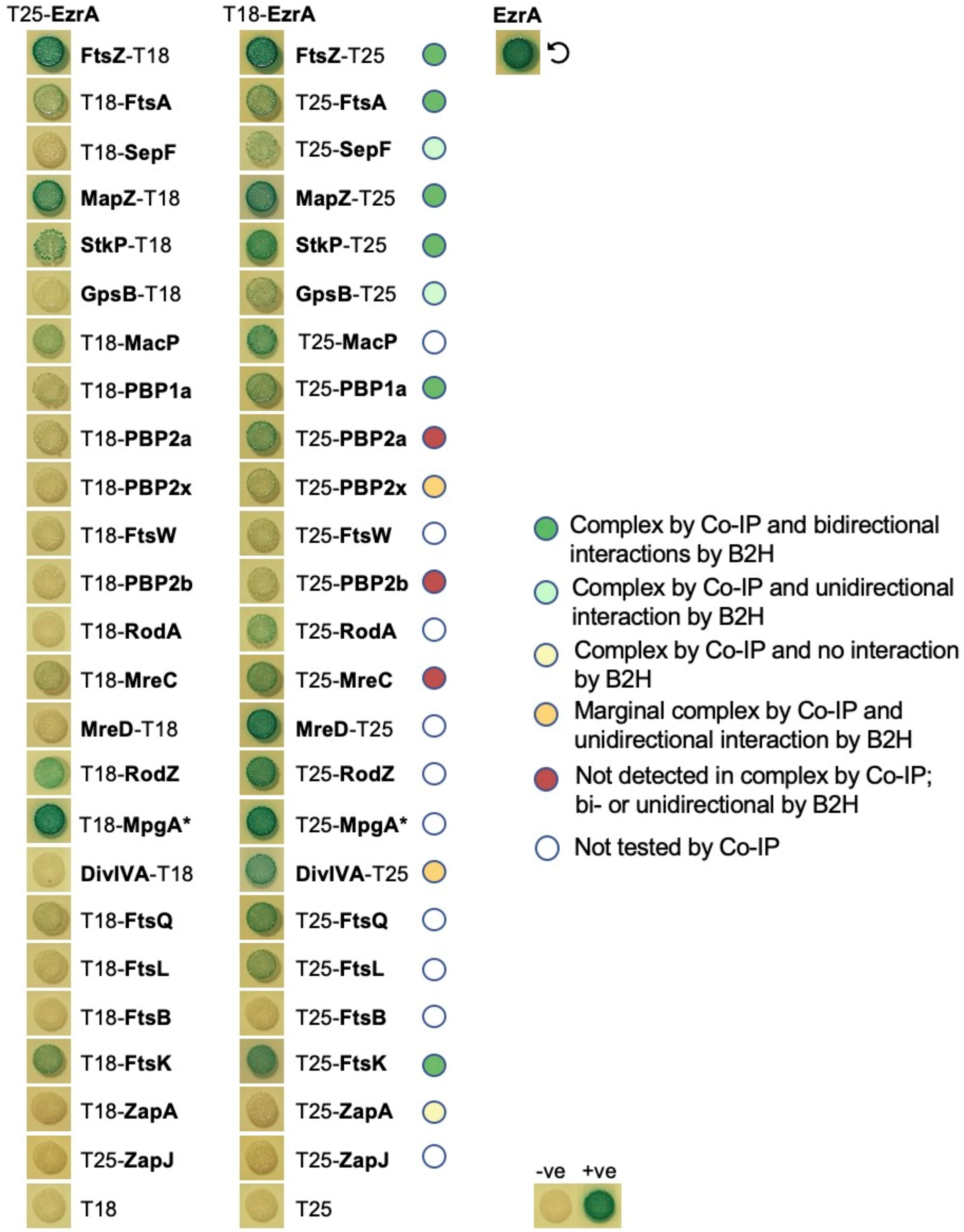

*MpgA was formerly MltG(*Spn*)

Supplementary Figure 21

Supplementary Figure 21. EzrA(*Spn*) interacts with different cell elongation and division proteins and with itself by B2H assays. EzrA interacts with FtsZ, FtsA, SepF, MapZ, StkP, GpsB, MacP, aPBP1a, aPBP2a, RodA, MreC, MreD, RodZ, MpgA (formerly MltG(*Spn*), DivIVA, FtsQ, FtsL, and FtsK, but apparent interactions were not detected between EzrA and FtsB, ZapA, or ZapJ. Weaker signals of interactions are detected between EzrA and bPBP2x, FtsW and bPBP2b. EzrA self-interactions are also shown. T25 or T18 fusions are expressed from low- or high-copy plasmids, respectively. Plasmid pairs pKNT25/pUT18 and pKT25-*zip*/pUT18C-*zip* were used as negative (-ve) and positive (+ve) controls. B2H assays were performed as described in the *Material and Methods*. The agar plates were photographed after 40 h at 30°C. B2H assays were performed at least twice with similar results.

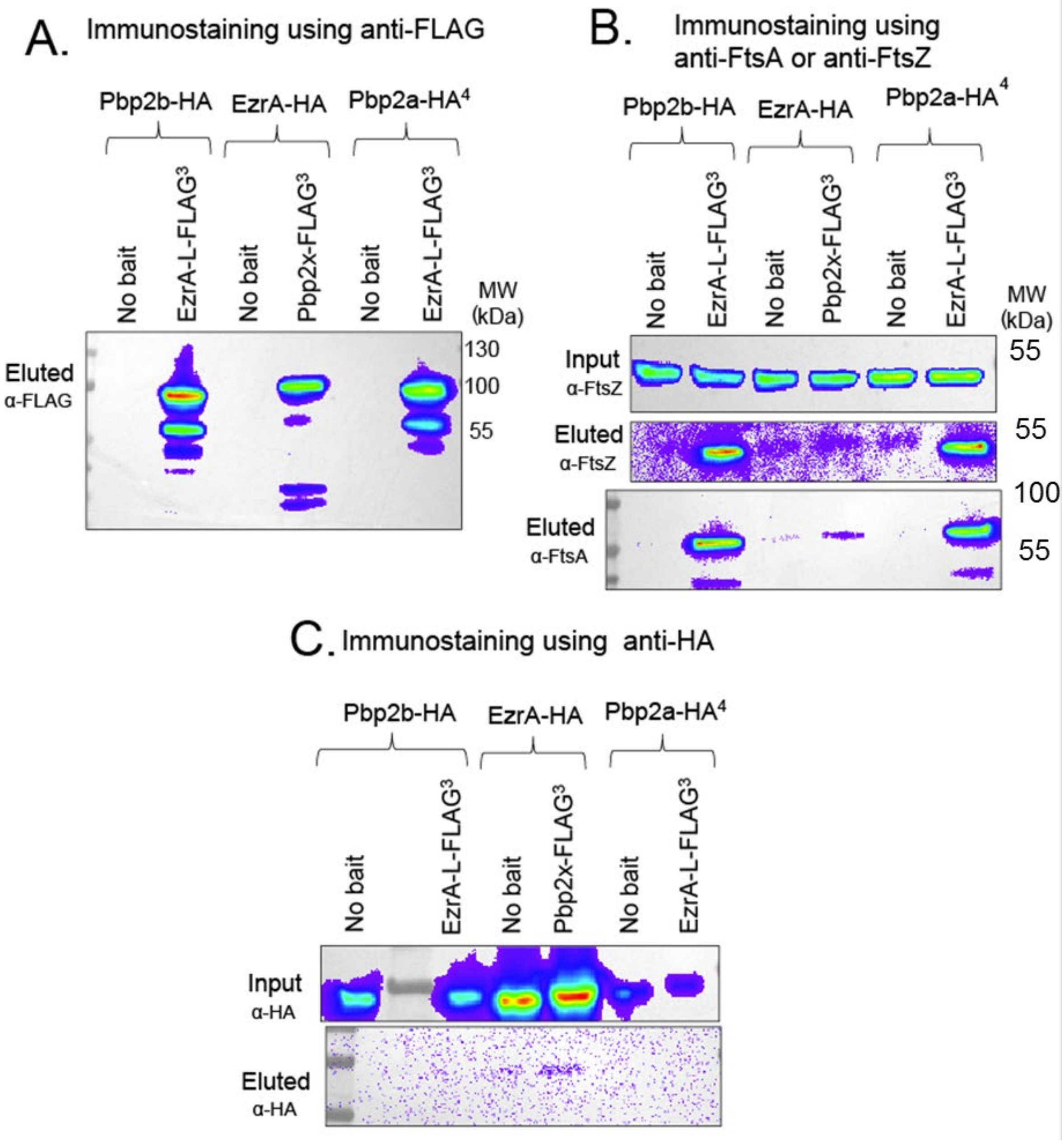

Supplementary Figure 22. Co-IP western blot membranes immunostained for bPBP2B-HA, EzrA-HA, aPBP2a-HA^4, FtsZ, FtsA (prey proteins) and bPBP2x-FLAG^3, EzrA-FLAG^3 (bait proteins) show a lack of association between three PBPs (bPBP2x, bPBP2b, aPBP2a) and FtsZ, FtsA, or EzrA. (A) Immunostaining using anti-FLAG to show the presence of EzrA-FLAG^3 and bPBP2x-FLAG^3. (B) Immunostaining using anti-FtsZ show relatively equal amount of FtsZ in the input fractions, while FtsZ and FtsA are eluted in the presence of EzrA-FLAG^3 but not bPBP2x-FLAG^3. (C) Immunostaining using anti-HA show lack of association detected between EzrA with bPBP2b, bPBP2x with EzrA, and EzrA with aPBP2a. See Table 2 and Table 3 for quantitation and strain numbers.

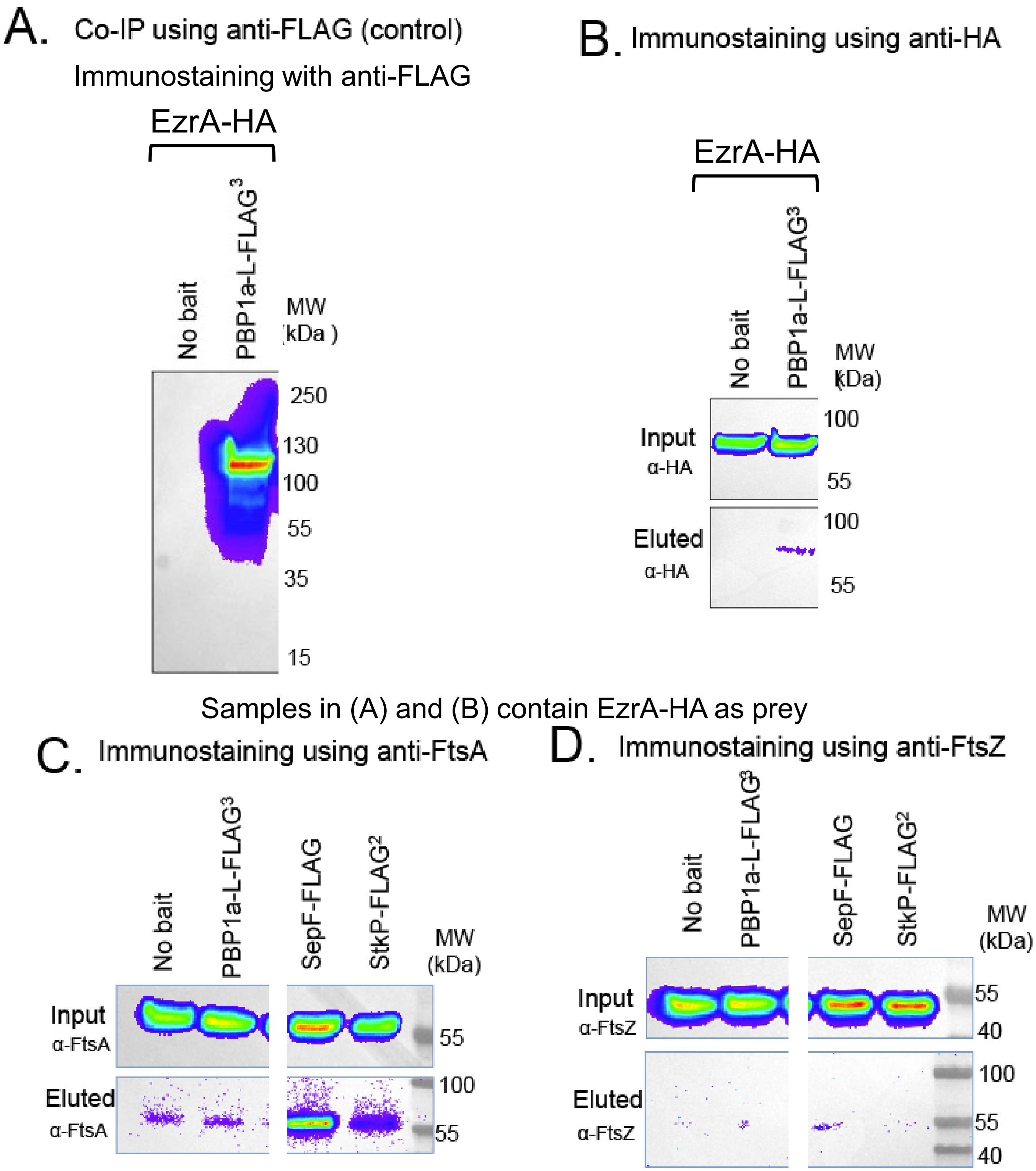

Supplementary Figure 23. Co-IP western blot membranes immunostained for EzrA-HA, FtsZ, FtsA (prey proteins) and aPBP1a-FLAG^3, SepF-FLAG, and StkP-FLAG (bait proteins) show complex associations. (A) Immunostaining using anti-FLAG to show the presence of aPBP1a-FLAG^3. (B) Immunostaining using anti-HA show relatively equal amount of EzrA-HA in the input fractions, while EzrA-HA is eluted in the presence aPBP1a-FLAG^3. (C) Immunostaining using anti-FtsA show relatively equal amount of FtsA in the input fractions, while FtsA is eluted in the presence SepF-FLAG and StkP-FLAG^2, (D) Immunostaining using anti-FtsZ show relatively equal amount of FtsZ in the input fractions, while a lack of FtsZ pulled down in any eluted fraction. See Table 2 and Table 3 for quantitation and strain numbers.

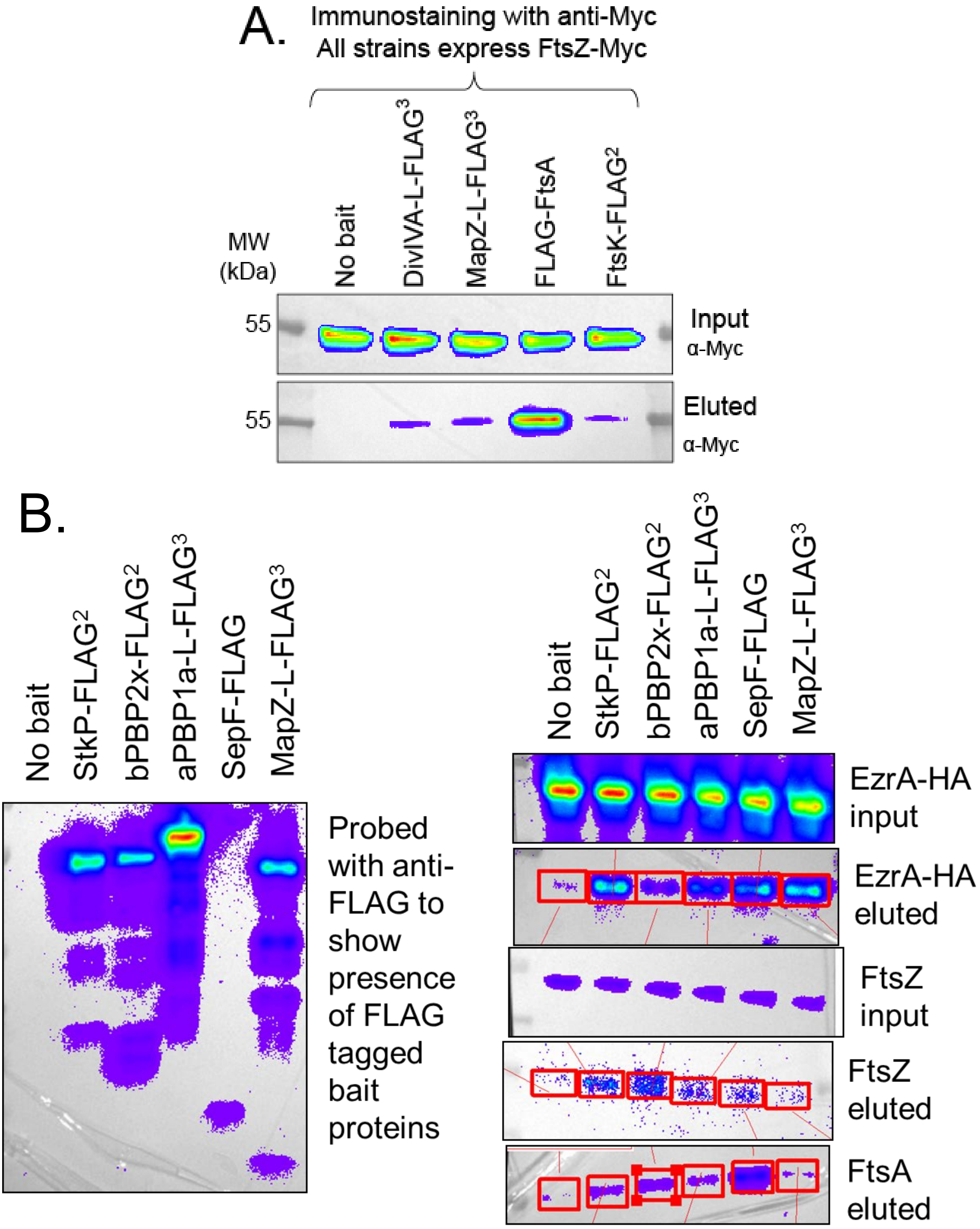

Supplementary Figure 24

Supplementary Figure 24. Co-IP western blot membranes immunostained for FtsZ-Myc, FtsZ, FtsA and EzrA-HA (prey proteins) and StkP-FLAG[2], bPBP2x-FLAG[2], aPBP1a-FLAG[3] SepF-FLAG, and MapZ-FLAG[3] (bait proteins) show complex associations. (A) Immunostaining using anti-Myc show relatively equal amount of FtsZ-Myc in the input fractions, while FtsZ-Myc is eluted in all fractions with the exception of no bait negative control. (B) (Left membrane) Immunostaining using anti-FLAG show the presence of bait proteins. (Right membrane) Immunostaining using anti-HA show relatively equal amount of EzrA-HA in the input fractions, while EzrA-HA is eluted in the presence all proteins although to different extents. Immunostaining with anti-FtsA from eluted fractions shows different amount of FtsA eluted from each fraction. The red boxes are examples of uniform size regions that were chosen to calculate "Mean ratios" in Table 2 and Table 3. See Table 2 and Table 3 for quantitation and strain numbers used in these experiments.

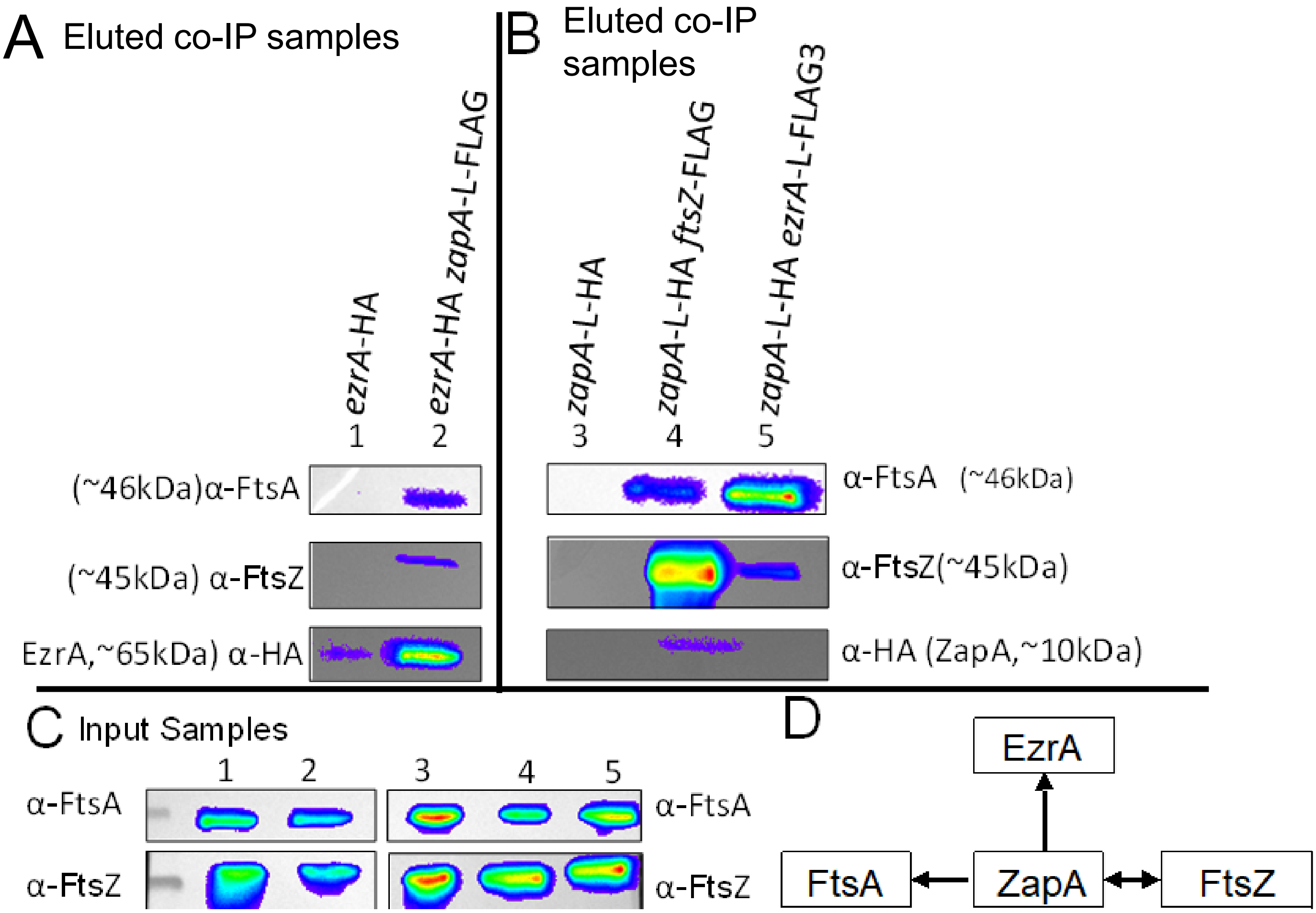

Supplementary Figure 25. Co-Immunoprecipitation experiments reveal ZapA forms complexes with FtsZ, FtsA, and EzrA. Co-IP experiments were performed as described in the *Materials and Methods*. Data is representative of two independent biological replicates. Predicted molecular weights (MW) of proteins are; ZapA-FLAG (10kDa), FtsZ (45kDa), FtsA (46kDa), and EzrA (65kDa). (A) Blots where EzrA-HA is used as the prey. Lane 1 has non FLAG-tagged ZapA^{+} strain as no bait negative control. Lane 2 uses ZapA-FLAG as the bait. Top panel, blot was probed with anti-FtsA primary antibody. Middle panel, blot was probed with anti-FtsZ primary antibody. Bottom panel, blot was probed with anti-HA primary antibody to detect prey EzrA-HA. (B) Blots where ZapA-HA is used as the prey. Lane 1 has untagged FLAG strain as no bait. Lane 2 uses FtsZ-FLAG as the bait. Lane 3 uses EzrA-FLAG^{3} as the bait. Top panel, blot was probed with anti-FtsA primary antibody. Middle panel, blot was probed with anti-FtsZ primary antibody. Bottom panel, blot was probed with anti-HA primary antibody to detect prey ZapA-HA. (C) Western blot results for the inputs for Co-IP experiments demonstrating relatively similar loading of cell lysates. Top panels indicate use of anti-FtsA primary antibody. Bottom panels indicate use of anti-FtsZ primary antibody. (D) Schematic of detected interactions. Direction of arrows indicate the ability of the protein when used as the bait to pulldown the prey protein in a complex (bait→prey). For computed mean ratios of proteins detected see Table 3.

A

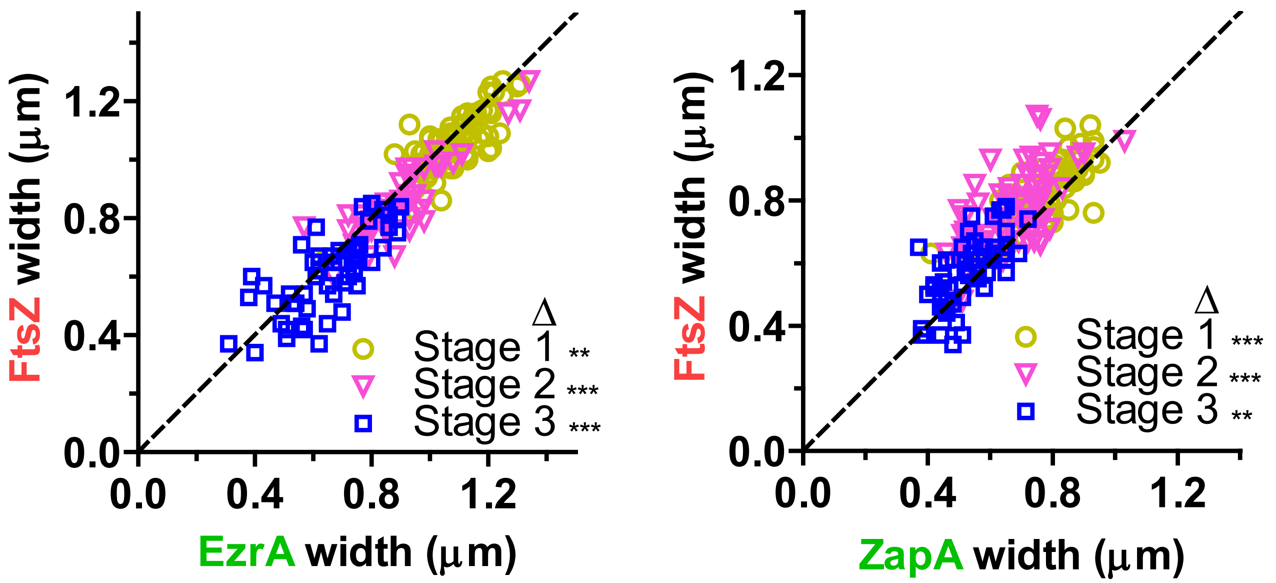

B

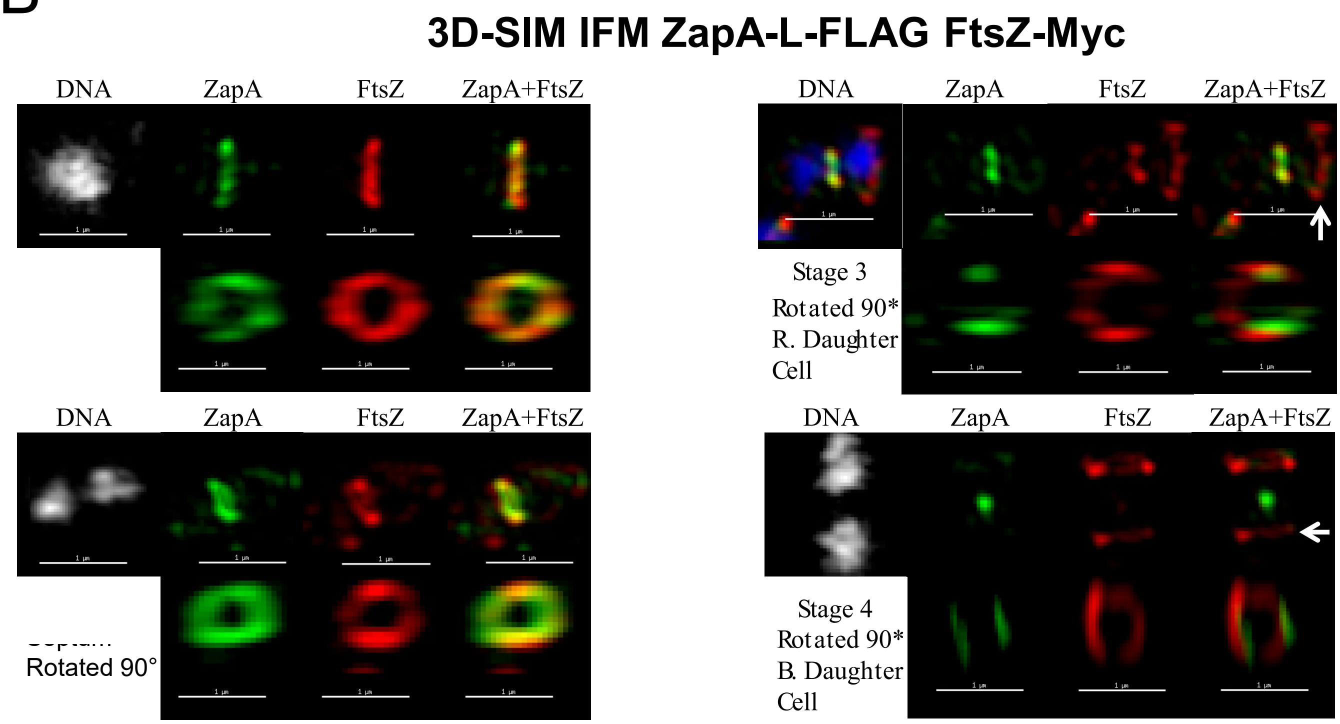

Supplementary Figure 26

Supplementary Figure 26. 3D-Organization of *Spn* ZapA-, FtsZ-, and EzrA-rings assessed by 2D-ring diameter measurements and 3D-SIM IFM. Strains IU8681 (*ftsZ*-Myc *ezrA*-FLAG[3]) and strain IU10752 (*ftsZ*-Myc *zapA*-FLAG) were grown to mid-exponential phase in BHI broth at 37°C and processed for dual-protein IFM with DAPI labeling as described in *Materials and Methods*. (A) Scatter plot of the paired widths from the same cells of FtsZ and EzrA (left plot) or FtsZ and ZapA (right plot) fluorescent immunolabeled regions at the actively dividing septa of strain IU8681 or IU10752 at division stages 1-3 (averaged cells are shown in Figure 10B). Width measurements and plotting were done using IMA-GUI program (see *Materials and Methods*). Statistical analysis was performed as described previously (Tsui *et al.,* 2014) where ** indicates $P<0.01$ and *** indicates $P<0.001$. Septal widths of stage 4 cells were not analyzed, because FtsZ or EzrA may have been missing from old sites of septation. (B) Representative 3D-SIM IFM and DAPI images obtained of strain IU10752 at different division stages (n=5 per stage). Each panel is a different cell in which FtsZ, ZapA, and DAPI are localized. DNA (DAPI-stained image) is pseudocolored white (i, ii, iv) or blue (ii, v). ZapA and FtsZ are pseudocolored green and red, respectively. The first row of each image represents images captured in the *xy* plane, while second-row images were obtained by rotating a section of the cell around the *x* or *y* axis, to illustrate the *z*-plane (i) Stage 1 cell showing ZapA and FtsZ-ring septal colocalization. (ii) Stage 2 cell showing ZapA and FtsZ-rings septal colocalization. (iii) Stage 3 cell showing ZapA concentrated at the septum of the cell while sparse at equators of two future dividing daughter cells. FtsZ is both at the septum and at equators of two future dividing daughter cells. Bottom panels are the right daughter cell rotated 90 degrees along the Y-axis. (iv) Late-divisional cells showing that daughter cells contain concentrated FtsZ-rings at equators of future dividing cells but sparse amounts of ZapA. A concentrated dot of ZapA at the former actively dividing septum can be seen whereas FtsZ shows a sparse dot. Bottom panels show bottom daughter cell rotated 90 degrees along Y-axis. Scale bar = 1µm. Arrows indicate equatorial ring plane that was chosen for rotation, shown in the second row of the corresponding cell.

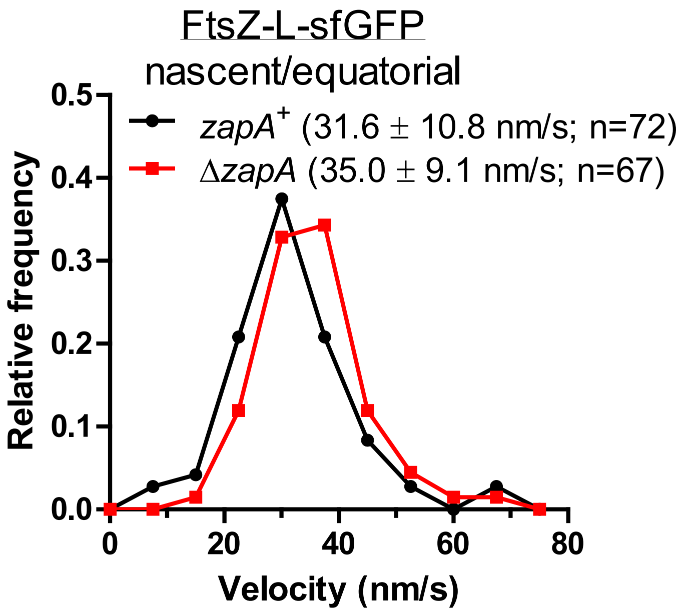

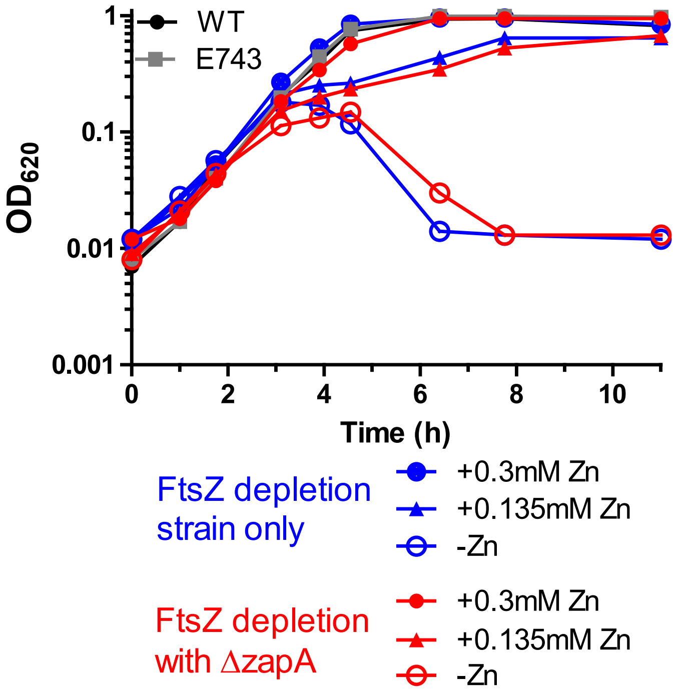

C

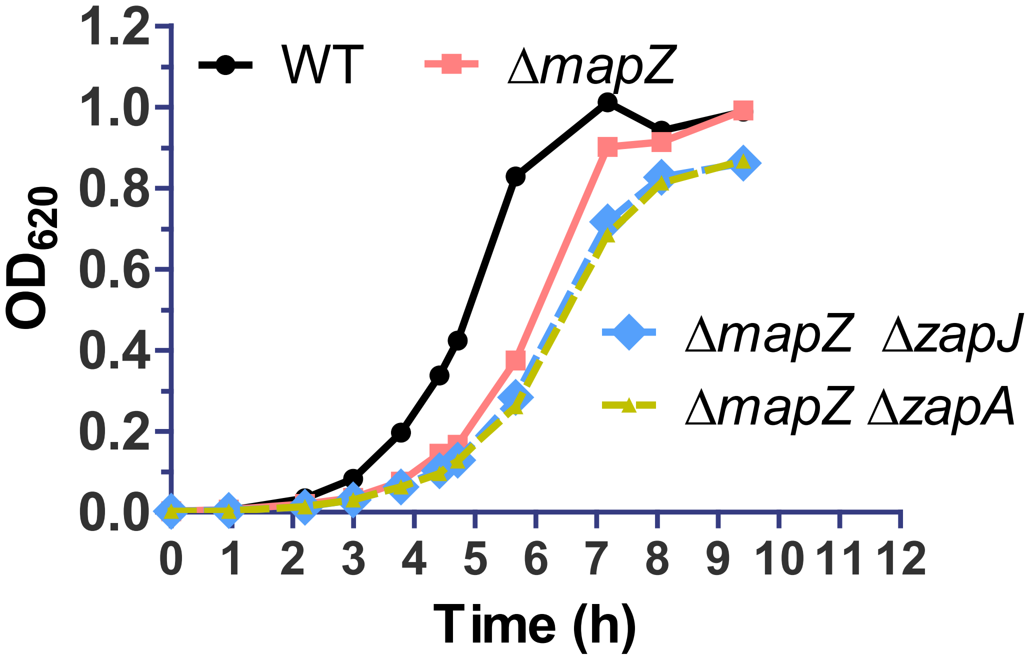

Supplementary Figure 27

Supplementary Figure 27. Deletion of *zapA*(*Spn*) does not affect FtsZ treadmilling velocity or growth curve when combined with FtsZ-depletion, but Δ*zapA* or Δ*zapJ* does show synthetic defects when combined with Δ*mapZ.* (A) Histogram displaying FtsZ-sfGFP treadmilling velocities obtained by TIRFm and kymograph analysis. Black line depicts control strain *zapA*$^{+}$ (IU9985); red line depicts Δ*zapA* markerless strain (IU14131). Values were obtained from two independent biological replicates. (B) Representative growth curve of FtsZ complemented or depleted cells in *zapA*$^{+}$ or Δ*zapA* backgrounds. Black line indicates growth of WT (IU1945). FtsZ depletion was performed in parent cells (IU8124 Δ*ftsZ*//P_{Zn}-*ftsZ*$^{+}$) or Δ*zapA* mutants IU10843 (Δ*zapA* Δ*ftsZ*//P_{Zn}-*ftsZ*$^{+}$) with the indicated amount of $ZnCl_2$. Shown are growth curve of WT cells (IU1945) or Δ*zapA* mutants (E743). Experiment was performed twice with similar results. (C) Representative growth curve of Wild-type cells (IU1824), Δ*mapZ* (IU15100)*,* Δ*mapZ* Δ*zapA* (IU15107)*,* Δ*mapZ* Δ*zapJ* (IU15110). Graph shown is representative from two or more independent biological replicates.

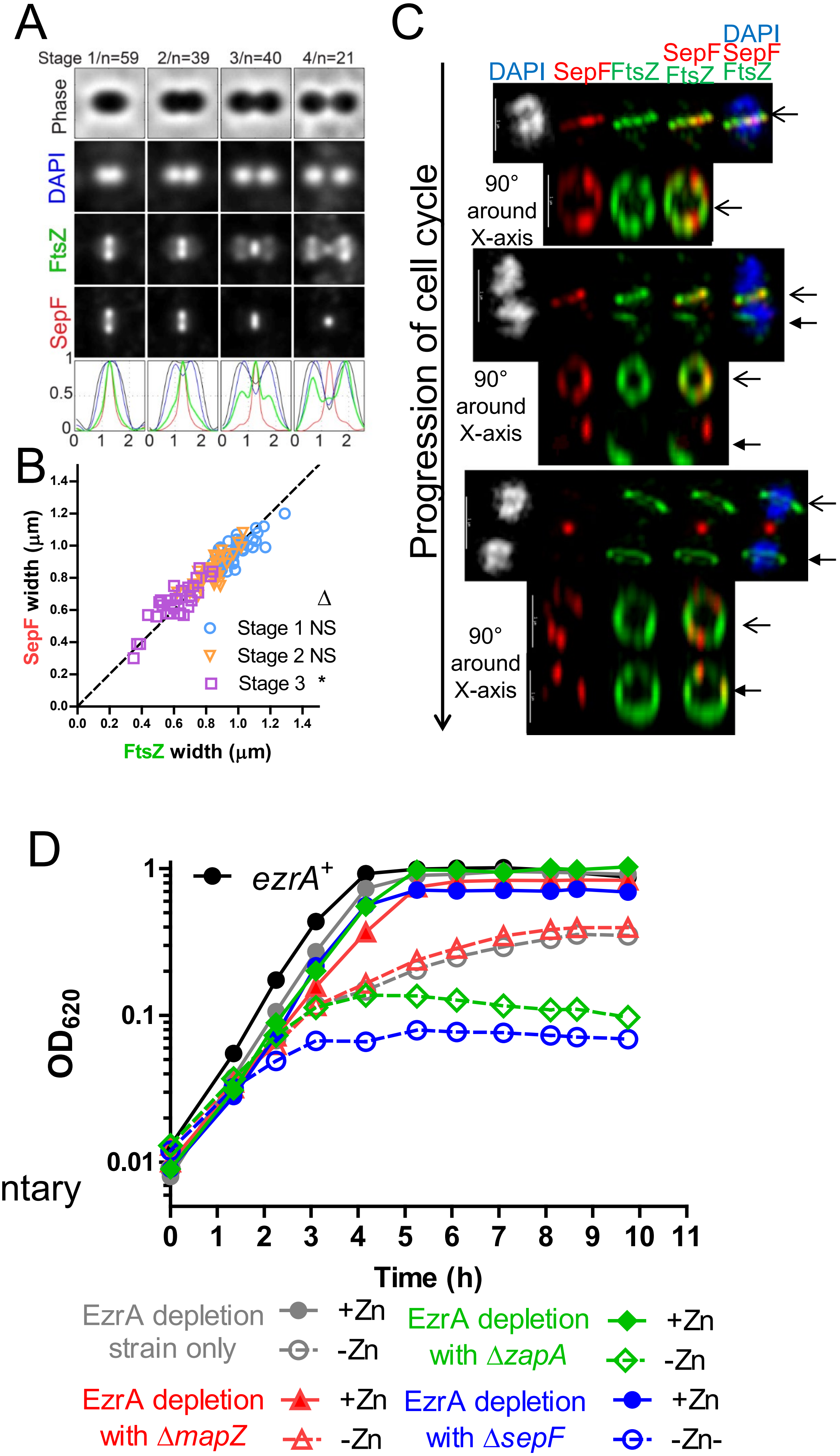

Supplementary Figure 28

Supplementary Figure 28. Late divisome arrival of SepF(*Spn*) relative to FtsZ and synthetic defects of Z-ring regulators when combined with EzrA-depletion. (A-C) IFM was performed with IU8596 (SepF-HA FtsZ-Myc) from cells grown in BHI broth. Data are from two independent biological replicates. (A) 2D-cell averages of IU8596. (B) Paired widths of SepF-HA vs FtsZ-Myc as described in (Tsui *et al.,* 2016). (C) Representative 3D-SIM images of IU8596. Arrows indicate corresponding daughter ring that was analysed by rotation. (D) Combined defects in growth curve of EzrA depletion with putative Z-ring regulator mutants, Δ*zapA* and Δ*sepF,* but not Δ*mapZ*. Growth curve of EzrA complemented or depleted cells in Z-ring regulator backgrounds. EzrA depletion was performed in parent strain (IU8799 Δ*ezrA*//*bgaA*::P_{Zn}-*ezrA*$^{+}$), Δ*mapZ* mutants IU9548 (Δ*mapZ* Δ*ezrA*//*bgaA*::P_{Zn}-*ezrA*$^{+}$), Δ*zapA* mutants IU9550 (Δ*zapA* Δ*ezrA*//*bgaA*::P_{Zn}-*ezrA*$^{+}$), and Δ*sepF* mutants IU9552 (Δ*sepF* Δ*ezrA*//*bgaA*::P_{Zn}-*ezrA*$^{+}$) from starting OD_{620}≈0.01.

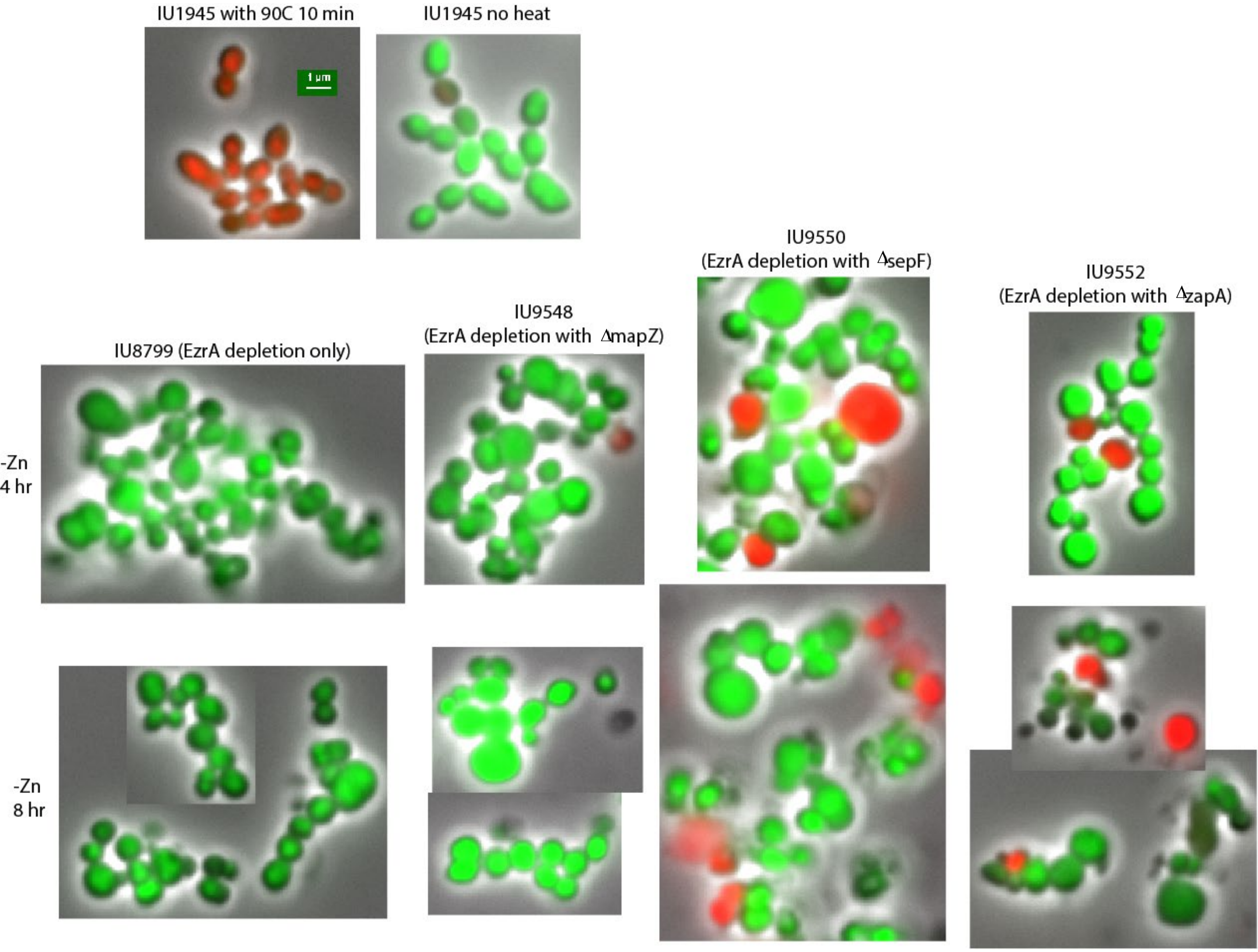

Supplementary Figure 29. Live/Dead staining of EzrA-depleted (*Spn*)cells in different genetic backgrounds. Shown are representative micrographs of cells depleted for EzrA for the indicated amount of time (4 or 8 h) and labeled with live dead staining. Green indicates live cells whereas red indicates dead cells. Cells were labeled as described in *Materials and Methods* and strain numbers correspond to genotypes listed in Supplementary Table 1.

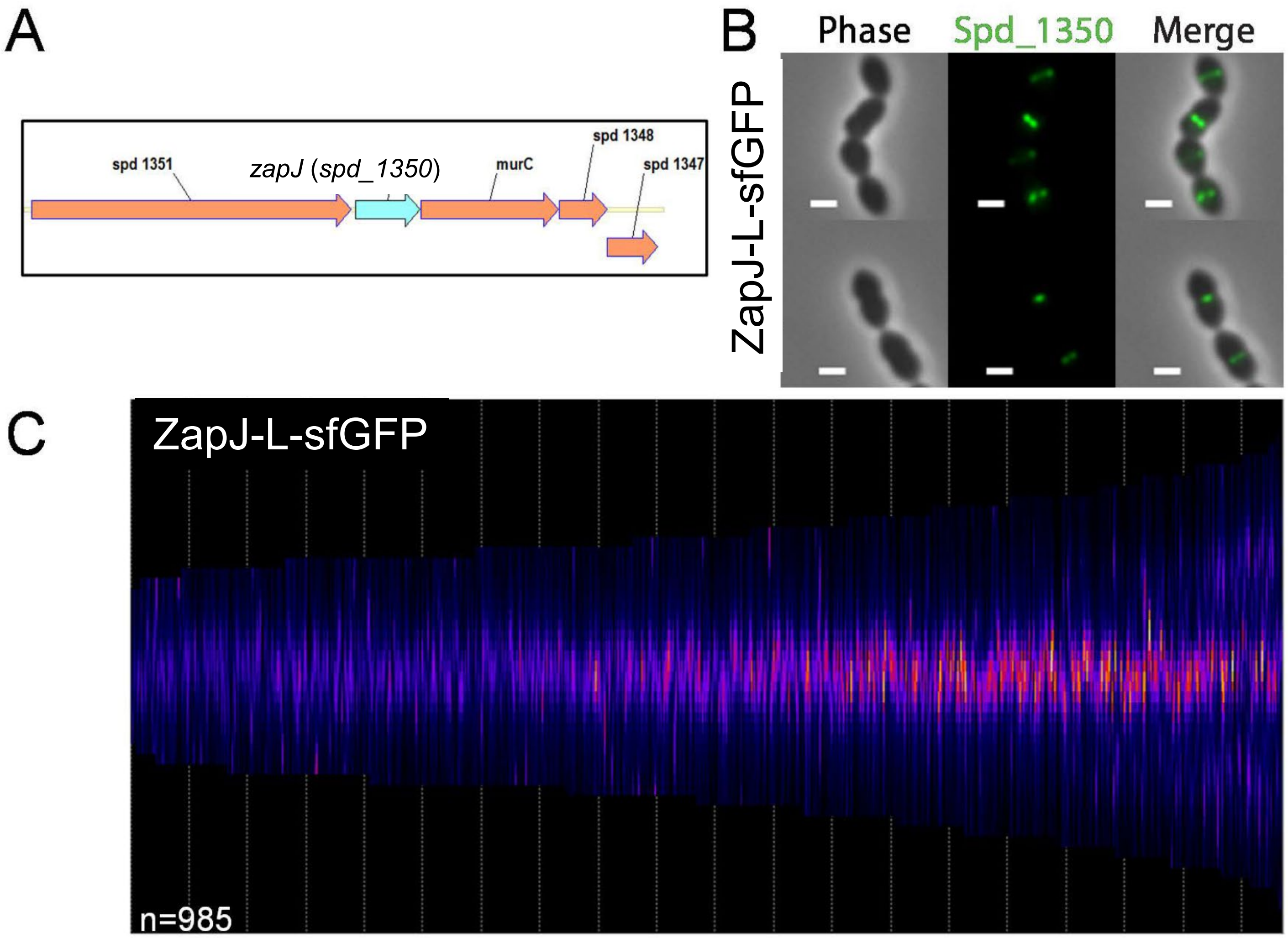

Supplementary Figure 30. Genetic loci of *zapJ*(*Spn*) and cellular localization of ZapJ. (A) Genetic arrangement of *spd_1350* in *S. pneumoniae* D39 chromosome. (B) Representative images of 2D PCm and EFm of strain IU13822 (*zapJ-sfgfp*) grown in BHI broth at 37°C. Scale bars are 1 µm. (C) Demograph of ZapJ-sfGFP localization. Cells were grown in BHI broth at 37°C (n=985). Data were obtained from two or more independent biological replicates.

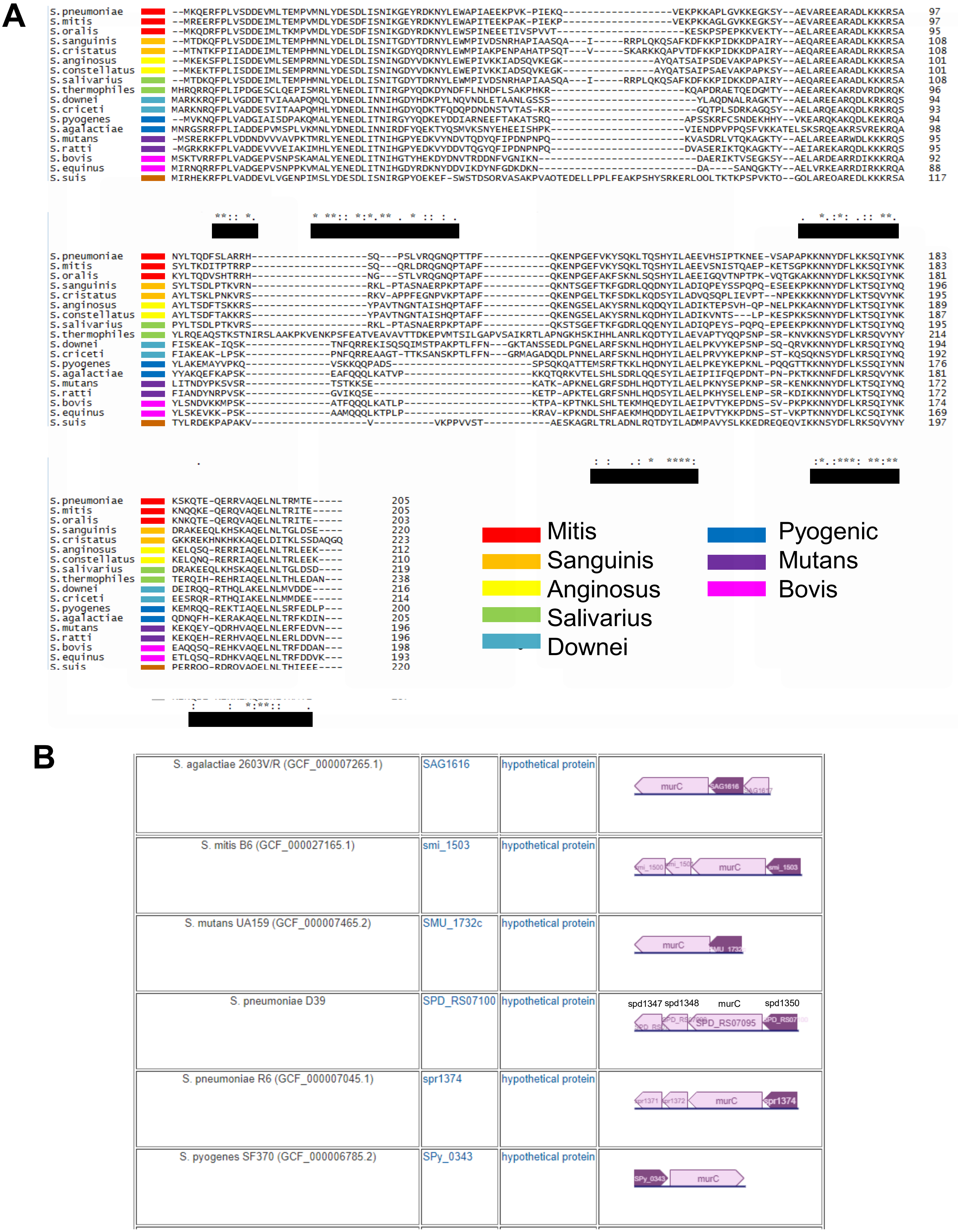

Supplementary Figure 31

Supplementary Figure 31. ZapJ is conserved in *Streptococci*. (A) Alignment of ZapJ homologs from *S. pneumoniae* D39 to other bacterial species. *S.pneumoniae* ZapJ residues (accession number WP_000808215.1) were aligned with corresponding residues of ZapJ homologs from other streptococcal species including *S. mitis* (YP_003446605.1), *S. oralis* (WP_000806743.1), *S. sanguinis* (WP_011837373.1), *S. cristatus* (WP_005591897.1), *S. anginosus* (WP_003023604.1), *S. constellatus* (WP_006268547.1), *S. salivarius* (WP_101772179.1), *S. thermophilus* (WP_011225424.1), *S. downei* (EFQ56587.1), *S. cricetti* (EFQ56587.1), *S. pyogenes* (WP_002985931.1), *S. agalactiae* (WP_001079334.1), *S. mutans* (WP_002262544.1), *S. ratti* (WP_003087037.1), *S. bovis* (WP_003066174.1), *S. equinus* (WP_004233035.1), *S. suis* (ABP90687.1), Species are color-coded depending on group type. Streptococcal species were chosen from each of 8 streptococci groups (Richards *et al*. 2014), one ungrouped streptococcal species (*S. suis*), and three outgroup species. Alignment was made using Clustal Omega with default parameters (Sievers *et al.,* 2011). Species name is on left, amino acid sequence is in middle, protein length on right. Black bars designate tracts of conserved residues that may be regions of conserved function. Asterisks, identical residues; colons, conserved residues; periods, semi-conserved residues. (B) Screenshot of different *zapJ* genes (dark purple) encoding ZapJ orthologs in different streptococci species obtained from BioCyc website (Karp et al 2019; https://biocyc.org/). *zapJ* orthologs were not found in genomes of bacteria other than streptococci. The follow organisms genomes were checked but no orthologue was found: *B. subtilis 168, C. glutamicum* ATCC 13032, *S. aureus* NCTC 8325, *S. coelicolor* A3(2), *T. denticola* ATCC 35405 (GCF_000008185.1), *L. lactis* IL1403, *E. faecalis* OG1RF. *murC* is annotated as SPD_RS07095 in the *Spn* D39 genome under BioCyc.
